# Comparative analysis of mitochondrial genomes of *Epimedium* L. reveals heterogeneity in structure, synteny, intercellular gene transfer, and RNA editing

**DOI:** 10.3389/fpls.2025.1701895

**Published:** 2025-11-24

**Authors:** De Xu, Juan Huang, Qianqian Ma, Tao Wang, Zheming Zhang, Qiang Wang, Zhide Wang, Zhou Xie, Xue Liu, Liang Fu

**Affiliations:** 1 Institute of Chinese Materia Medica, Dazhou Academy of Agricultural Sciences, Dazhou, China; 2 Chongqing Key Laboratory of Traditional Chinese Medicine Resource, Endangered Medicinal Breeding National Engineering Laboratory, Chongqing Academy of Chinese Materia Medica, Chongqing, China

**Keywords:** *Epimedium* L., mitochondrial genome, comparative analysis, gene transfer, RNA editing events

## Abstract

*Epimedium* L. is a taxonomically complex genus comprising 68 species worldwide, yet its mitochondrial genome (mitogenome) remains unexplored. The complete genomes of *Epimedium pubescens*, *Epimedium wushanense*, and *Epimedium sagittatum* were assembled using Illumina and Nanopore sequencing. The *Epimedium* mitogenomes displayed diverse structural variation, including a single circular molecule in *E. sagittatum* (324,345 bp), two circular molecules in *E. wushanense* (281,026 and 72,800 bp), and a multipartite structure with three circular chromosomes (171,784, 76,915, and 71,519 bp) and one linear chromosome (26,149 bp) in *E. pubescens*. Each species contained 58 unique genes, including 36 protein-coding genes (PCGs), 19 transfer RNAs (tRNAs), three ribosomal RNAs (rRNAs), and abundant repetitive elements [77–89 simple sequence repeats (SSRs) and 169–380 dispersed repeats]. A total of 642 cytidine (C)-to-uridine (U) RNA editing sites were predicted across 35–36 PCGs, with experimentally validated edits generating start and stop codons, revealing species-specific editing profiles. Nine mitochondrial plastid DNA (MTPT) fragments (4.4–7.5 kb) were identified per species, containing six to nine complete genes. Three *Epimedium* species and related taxa exhibited conserved mitogenome regions alongside extensive gene rearrangements and inversions. Phylogenetic analysis based on 29 conserved PCGs strongly supported a monophyletic *Epimedium* clade (100% bootstrap), with *E. sagittatum* and *E. pubescens* forming a sister group to *E. wushanense*. This study provides the first comprehensive view of *Epimedium* mitogenome architecture, RNA editing, and evolutionary relationships, enriching our understanding of its mitochondrial evolution and taxonomy.

## 1 Introduction

*Epimedium* L., the largest herbaceous genus within the Berberidaceae, encompasses approximately 68 species distributed mainly across temperate mountainous regions from East Asia to Northwest Africa (Stearn, 2002). China represents the modern center of *Epimedium* diversity, harboring 58 species (57 of which are endemic) (Stearn, 2002; Ying, 2002; Zhang et al., 2007). Additionally, four species are distributed in the Mediterranean and Western Asian regions (*Epimedium alpinum*, *Epimedium pubigerum*, *Epimedium pinnatum*, and *Epimedium perralderianum*), while five other species (*Epimedium koreanum*, *Epimedium grandiflorum*, *Epimedium sempervirens*, *Epimedium trifoliatobinatum*, and *Epimedium diphyllum*) are distributed in Japan. Among them, *E. koreanum* is found in China, Korea, and Japan, reflecting a broader East Asian distribution pattern (Stearn, 2002; Ying, 2002). Furthermore, *Epimedium macrosepalum* and *Epimedium elatum* are distributed in Far Eastern Russia and Kashmir, respectively. China is recognized as the modern center of the distribution and diversity of the genus *Epimedium*. These endemic species hold significant economic value and have been used in traditional Chinese medicine for centuries. Several species, including *Epimedium pubescens*, *Epimedium wushanense*, *Epimedium sagittatum*, *Epimedium brevicornu*, and *E. koreanum*, are officially listed in the Chinese Pharmacopoeia (2025) and have long been used in traditional medicine for their tonic and therapeutic properties (Ma et al., 2011; Guo et al., 2022; Commission, 2025). China is the main supplier of *Epimedium* herbs and extractions, and to date, most *Epimedium* medicinal resources (such as *E. pubescens*, *E. wushanense*, *E. sagittatum*, *E. brevicornu*, and *E. koreanum*) are primarily harvested from the wild, with limited artificial cultivation. However, extensive exploitation of wild resources and the presence of morphologically similar species have led to confusion in species identification, inconsistent medicinal quality, and an urgent need for improved taxonomic resolution and sustainable utilization.

Taxonomic classification within *Epimedium* remains one of the most challenging issues in the Berberidaceae due to extensive morphological convergence and hybridization. Morphological differences among *Epimedium* species are often subtle and overlapping, making accurate species identification a persistent challenge. Since 1975, various taxonomic approaches have been applied, such as classical morphology (Stearn, 2002), chemical classification (Xie et al., 2010), and phylogenetic analysis based on karyotypes and molecular markers (Sun et al., 2005; Sheng et al., 2010; De Smet et al., 2012; Zhang et al., 2014). In China, taxa frequently exhibit complex morphological variation despite minimal detectable genetic divergence, which complicates the application of Stearn’s widely accepted classification system (Guo et al., 2022). Many species are distinguished only by minor differences in floral or leaf characteristics. The Chinese Sect. (*Diphyllon*), which exhibits the highest species diversity within *Epimedium*, has been the source of numerous taxonomic controversies (Xu et al., 2008; Guo et al., 2018; Xu et al., 2019). Although morphological, chemical, and plastid-based phylogenetic studies have provided preliminary insights, discrepancies persist between morphological and molecular evidence, underscoring the need for additional genomic resources to refine species boundaries and evolutionary relationships.

The plant mitochondrial genome (mitogenome), generally ranging from 66 kb to 12 Mb in size (Sloan et al., 2012; Putintseva et al., 2020), is involved in multiple metabolic processes and plays a vital role in energetic metabolism, gene expression, stress response, and plant growth in many seed plants (Mackenzie and Mcintosh, 1999; Best et al., 2020). Moreover, plant mitogenomes exhibit remarkable diversity in genome size, structural organization, mutation rates of protein-coding genes (PCGs), RNA editing capacity, gene content, and recombination mediated by repeat sequences (Allen et al., 2007; Wang et al., 2023). For example, the mitogenome of *Arabidopsis thaliana* (Sloan et al., 2018) and *Chlamydomonas reinhardtii* (Vahrenholz et al., 1993) are typically assembled as a single circular structure and a linear structure, respectively, whereas other species, such as *Stemona tuberosa* (Xu et al., 2025), *Stemona sessilifolia* (Xie et al., 2024), and *Angelica biserrata* (Wang et al., 2024), display complex multi-chromosomal structures. They can exist as circular (Wang et al., 2023), linear (He et al., 2021), or branched forms (Jackman et al., 2020). The synonymous substitution rate within plant mitogenomes is several to dozens of times lower than that of plastomes and nuclear genomes, and even 50 to 100 times lower than that of mammalian mitogenomes (Wolfe et al., 1987).

Functional genes in mitogenomes show considerable variation due to post-transcriptional RNA editing, which can lead to highly divergent gene sequences (Li et al., 2022). In addition, the evolutionary stability of a species is closely related to the ability of its genes to adapt to global environmental changes, a characteristic reflected in the Guanine-Cytosine content of higher plant mitogenomes (Ye, 2018). Currently, it is widely recognized that both mitochondrial and chloroplast genomes can improve phylogenetic resolution at lower taxonomic levels, and their data are frequently used to clarify relationships among plant groups. Although plastid genome studies have provided valuable insights into the phylogeny and evolution of *Epimedium* (*Epimedium acuminatum*, *Epimedium dolichostemon*, *Epimedium lishihchenii*, and *Epimedium pseudowushanense*) (Guo et al., 2022; Zhang et al., 2007), despite their significance, mitochondrial genomic data for *Epimedium* are completely lacking, limiting our understanding of its evolutionary dynamics and taxonomic framework.

Therefore, this study aimed to assemble and characterize the complete mitogenomes of three representative *Epimedium* species (*E. pubescens*, *E. wushanense*, and *E. sagittatum*) using Illumina and Nanopore sequencing technologies. Through comparative genomic and phylogenetic analyses, we sought to elucidate the structural organization, gene content, and evolutionary patterns of *Epimedium* mitogenomes, thereby providing new genomic evidence to support species identification, taxonomy, and evolutionary studies within the Berberidaceae.

## 2 Materials and methods

### 2.1 DNA extraction and sequencing

Three *Epimedium* species (*E. pubescens*, *E. wushanense*, and *E. sagittatum*) were collected from the Dazhou Academy of Agricultural Sciences in Dazhou, China. Genomic DNA was extracted from fresh, healthy leaves following the cetyltrimethylammonium bromide (CTAB) method (Doyle and Doyle, 1987).

For Illumina sequencing, 1 ng of DNA was used to construct a short-read library (average insert size of 350 bp), which was sequenced on the DNBseq platform (California, USA). For Oxford Nanopore sequencing, sequence libraries were generated using the SQK-LSK109 ligation kit following the manufacturer’s protocols. The prepared library was then loaded onto primed R9.4 Spot-on Flow Cells and sequenced using a PromethION sequencer (Oxford Nanopore Technologies, Oxford, UK) over 48-hour runs. Base calling of the raw data was conducted using GuPPy version 1.2.0.

### 2.2 Mitogenome assembly, annotation, and visualization

The GetOrganelle software (version 1.7.5) was used to assemble the draft graphical mitogenome using the following parameters: -R 20 -k 21,45,65,85,105 -P 1000000 -F embplant_mt. The Bandage software (version 0.8.1) (Wick et al., 2015) was used to visualize the graphical mitogenome results, and extended fragments from the plastome and nuclear genomes were manually removed. Subsequently, the Nanopore data were aligned to the circular mitogenome using the BWA software (version 0.7.17) (Li and Durbin, 2009). These data were crucial in resolving repeat regions within the mitogenome, leading to the complete assembly mitogenome. The annotation of PCGs was conducted with Geseq (version 2.03) and IPMGA (http://www.1kmpg.cn/pmga/) (Tillich et al., 2017; Li et al., 2024b) using five mitogenomes as reference, including *Aconitum carmichaelii* (NC_084324.1), *Aquilegia amurensis* (OR818043.1–OR818045.1), *Pulsatilla dahurica* (NC_071219.1), *Coptis omeiensis* (OP466724.1–OP466725.1), and *Corydalis pauciovulata* (NC_081934.1). Transfer RNA (tRNA) and ribosomal RNA (rRNA) within the mitogenome were annotated using the tRNAscan-SE (version 2.0.11) (Lowe and Eddy, 1997) and BLASTN software (version 2.13.0) (Chen et al., 2015), respectively. The annotation results were manually corrected using the Apollo software (version 1.11.8) (Lewis et al., 2002). The final assembly and annotation results were deposited in the National Center for Biotechnology Information (NCBI) database (https://www.ncbi.nlm.nih.gov/) under accession numbers: *E. sagittatum* (PV694670), *E. wushanense* (PV694989–PV694990), and *E. pubescens* (PV695558–PV695561).

### 2.3 PCR validation of graphical assembly results

Based on the graphical mitogenome results of three *Epimedium* mitogenomes, linkages between graphical contigs were designed using Primer BLAST (https://www.ncbi.nlm.nih.gov/tools/primer-blast/) (**Supplementary Table S1**). PCR amplification was carried out in a 50 μL reaction mixture containing 1 μL DNA template, 1 μL each of 10 μM forward and reverse primers, 25 μL of 2× EasyTaq SuperMix, and 22 μL ddH_2_O. The thermal cycling conditions were as follows: initial denaturation at 94°C (2 min); 35 cycles of 94°C (30 s), 58°C (30 s), and 72°C (1 min); with a final extension at 72°C (2 min). Amplification products were visualized on a 1% agarose gel, and fragment sizes were estimated using a 100–2,000-bp DNA marker (Sangon Biotech, Shanghai, China). The target bands were subsequently sequenced via the Sanger method, and chromatograms were analyzed using the SeqMan software.

### 2.4 Codon usage bias and repeat element analysis

PCGs were extracted using the PhyloSuite software (version 1.1.16) (Zhang et al., 2020a) with default parameters. Codon usage bias of PCGs from three *Epimedium* mitogenomes was analyzed using the MEGA software (version 7.0) (Kumar et al., 2016), and relative synonymous codon usage (RSCU) values were calculated. For simple sequence repeat (SSR), tandem repeat, and dispersed repeat analyses, multiple tools were employed: MISA (version 2.1) (https://webblast.ipk-gatersleben.de/misa/) (parameters: 1-10, 2-5, 3-4, 4-3, 5-3, and 6-3) (Beier et al., 2017), the Tandem Repeats Finder (TRF, version 4.09) (https://tandem.bu.edu/trf/trf.unix.help.html) (parameters: default) (Benson, 1999), and the REPuter server (parameters: default) (https://bibiserv.cebitec.uni-bielefeld.de/reputer/) (Kurtz et al., 2001), respectively. Visualization of repeat elements was accomplished using the Circos package (version 0.69.9) (Zhang et al., 2013) and Excel 2021.

### 2.5 Prediction and validation of RNA editing sites

RNA editing events were identified using the online tool PREPACT3 (available at http://www.prepact.de/) (Lenz et al., 2018), applying a threshold of 0.001. To validate RNA editing sites within the three *Epimedium* species mitogenomes, five sites of four PCGs (*nad1*, *rps10*, *atp6*, and *rps11*) were randomly selected for PCR amplification. The PCR primers for all selected PCGs were designed using Primer BLAST (**Supplementary Table S2**). Total RNA was reverse-transcribed into cDNA using the TransScript First-Strand cDNA Synthesis SuperMix kit. Both genomic DNA and cDNA templates were then subjected to PCR amplification (same as Section 2.3). The amplified products were subsequently sequenced using the Sanger method, and chromatograms were analyzed using the SeqMan software.

### 2.6 Homologous sequence between organelles and collinear analysis

The chloroplast genomes of three *Epimedium* species (*E. pubescens*, *E. wushanense*, and *E. sagittatum*) were assembled using GetOrganelle (version 1.7.5) with the following parameters: -R 10; -F embplant_pt. Three chloroplast genomes were annotated using the CPGAVAS2 software (version 2.0) (Shi et al., 2019). Homologous sequences between the mitochondrial and chloroplast genomes were analyzed using the BLASTN software (version 2.13.0) with default settings, and the results were visualized using the Circos package (version 0.69.9).

The mitogenome pairwise comparison was conducted using BLASTN (parameters: -evalue 1e−5, -word_size 9, -gapopen 5, -gapextend 2, -reward 2, -penalty −3). Homologous sequences longer than 0.5 kb were used to construct conserved collinearity blocks in the multiple synteny plot, which was visualized using MCScanX (Wang et al., 2012).

### 2.7 Phylogenetic analysis

Twenty-three complete mitogenomes belonging to three orders (Ranunculales, Proteales, and Magnoliales) were obtained from the NCBI database (**Supplementary Table S3**). A total of 29 conserved PCGs were extracted using the PhyloSuite software. Multiple sequence alignment was performed using the MAFFT software (version 7.505) with parameter -auto (Katoh et al., 2002). Phylogenetic analysis was conducted using the IQ-TREE software (version 1.6.12) with the following parameters: –alrt 1000 -B 1000 (Nguyen et al., 2015). The resulting maximum likelihood tree was visualized using the ITOL software (version 4.0) (Letunic and Bork, 2019).

## 3 Results

### 3.1 Characteristics of the three *Epimedium* mitogenomes

**Figure 1:**
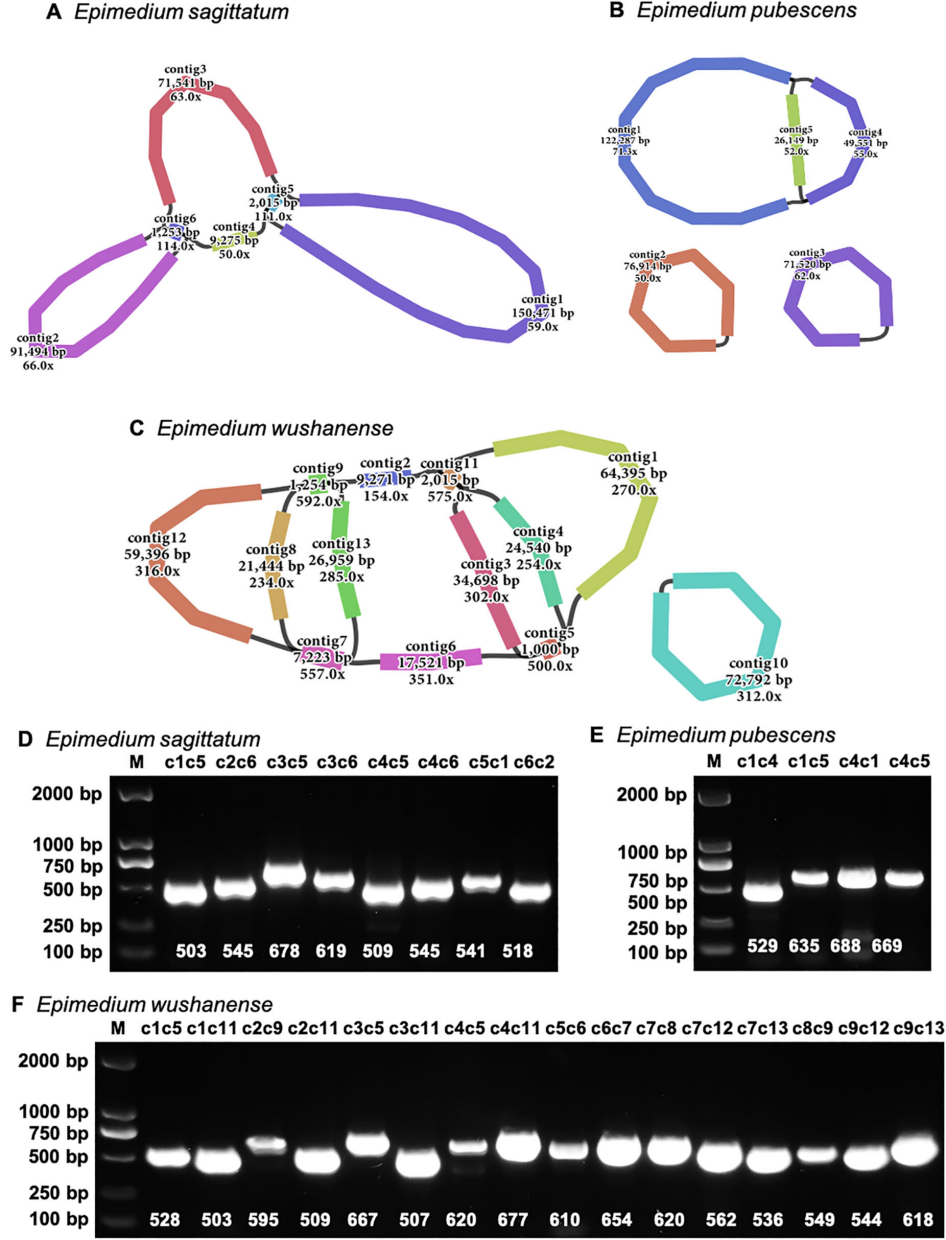
Unitig graphs of the mitogenomes of three *Epimedium* species and PCR validation of their contig linkages. Assembly graphs were shown for **(A)***Epimedium sagittatum*, **(B)***Epimedium pubescens*, and **(C)***Epimedium wushanense*. Corresponding PCR validation results for the contig linkage regions were presented for **(D)***E. sagittatum*, **(E)***E. pubescens*, and **(F)***E. wushanense*. In the electrophoresis gel image, each band represents a junction between two contigs. For example, in panel **(D)**, the bands correspond to the connections between contig1 and contig5 (c1c5), contig2 and contig6 (c2c6), and so on. The numbers below each band indicate the length of the PCR product.

**Figure 2:**
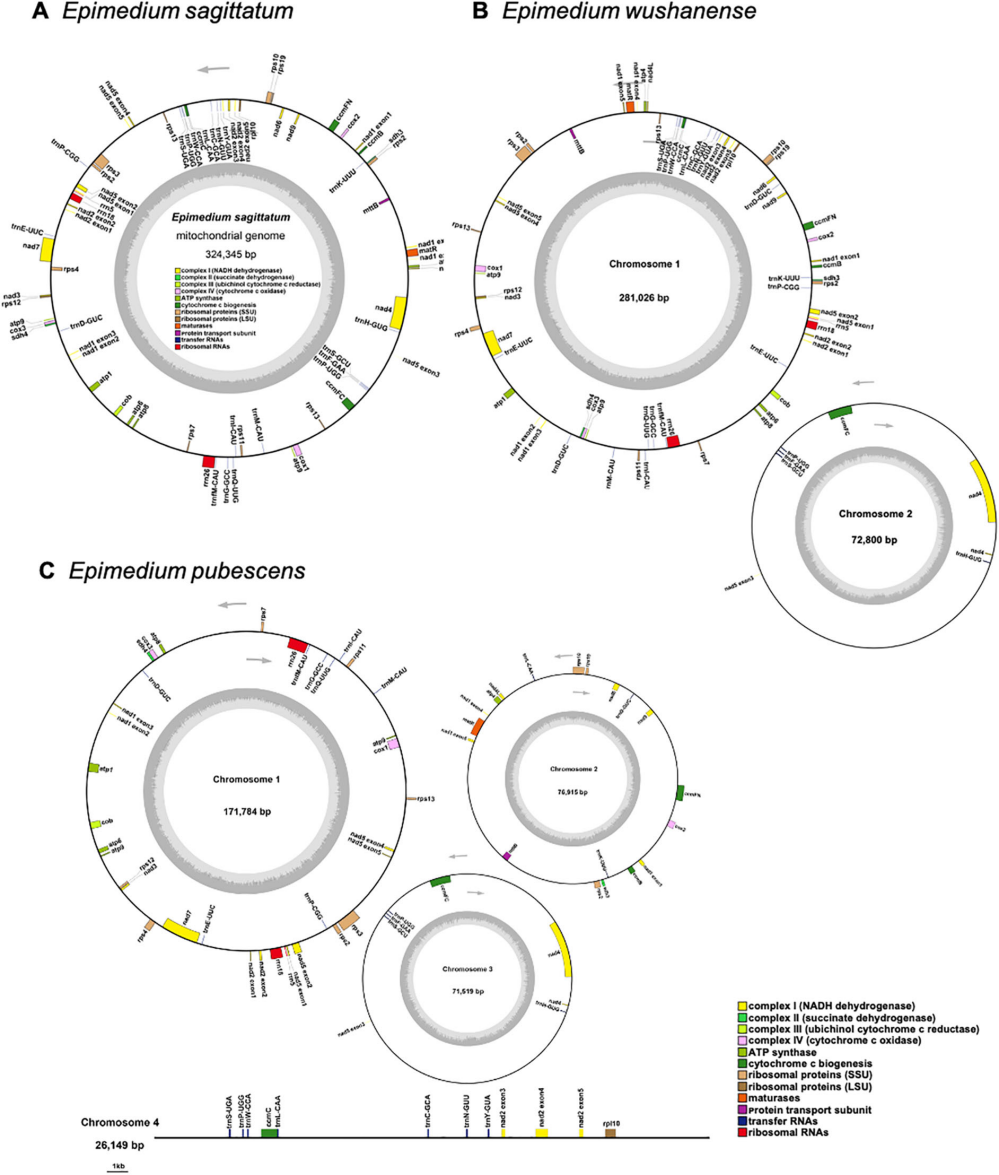
The map of the mitogenome of **(A)***Epimedium sagittatum*, **(B)***Epimedium wushanense*, and **(C)***Epimedium pubescens*. The arrows show transcriptional direction of each mitogenome. Genes with different functions are depicted using different colors.

Three high-quality *Epimedium* mitogenomes were assembled by integrating Illumina short-read and Nanopore long-read sequencing data. The final uniting graphs consisted of six contigs in *E. sagittatum*, five contigs in *E. pubescens* (including two circular contigs), and 12 contigs in *E. wushanense* (with one circular contig) (**Figures 1A–C**). To validate the accuracy of the graphical assemblies, primers were designed at the ends of selected contigs to test the connectivity between adjacent contigs. PCR validation and subsequent Sanger sequencing confirmed eight junctions in *E. sagittatum*, four junctions in *E. pubescens*, and 16 junctions in *E. wushanense* (**Figures 1D, F**; **Supplementary Figure 1**). Both the PCR product sizes and Sanger sequencing results supported the graphical assembly results. Finally, the mitogenome of *E. sagittatum* was simplified into a single circular molecule measuring 324,345 bp with a GC content of 46.91% (**Table 1**; **Figure 2A**). In contrast, the *E. wushanense* mitogenome was simplified into two independent circular molecules (**Table 1**; **Figure 2B**). The total length of the *E. wushanense* mitogenome was 353,826 bp, with an overall GC content of 46.58%. The larger molecule was 281,026 bp in length with a GC content of 46.82%, while the smaller one measured 72,800 bp with a GC content of 45.64%. Additionally, the mitogenome of *E. pubescens* exhibited a complex branched structure, inferred to consist of three circular chromosomes (chromosomes 1–3) and one linear chromosome (chromosome 4), totaling 346,367 bp in length with a GC content of 46.52% (**Table 1**; **Figure 2C**). The four chromosomes exhibited variation in length and GC content: chromosome 1 was the longest at 171,784 bp with a GC content of 47.02%, followed by chromosome 2 (76,915 bp, 45.81%), chromosome 3 (71,519 bp, 45.79%), and chromosome 4 (26,149 bp, 47.36%).

**Table 1:** Basic information for the mitogenome of *Epimedium sagittatum*, *Epimedium wushanense*, and *Epimedium pubescens*.

Species	Chromosome	Type	Length (bp)	GC content (%)
*E. sagittatum*	1	Circular	324,345	46.91
*E. wushanense*	1	Circular	281,026	46.82
	2		72,800	45.64
*E. pubescens*	1	Circular	171,784	47.02
	2	Circular	76,915	45.81
	3	Circular	71,519	45.79
	4	Linear	26,149	47.36

A total of 58 unique mitochondrial genes were annotated in the three *Epimedium* mitogenomes, consisting of 36 distinct PCGs, 19 tRNA genes, and 3 rRNA genes (**Table 2**). Among the 36 unique PCGs, 24 were classified as core genes, which included five ATP synthase genes (*atp1*, *atp4*, *atp6*, *atp8*, and *atp9*), nine NADH dehydrogenase genes (*nad1*, *nad2*, *nad3*, *nad4*, *nad4L*, *nad5*, *nad6*, *nad7*, and *nad9*), four cytochrome *c* biogenesis genes (*ccmB*, *ccmC*, *ccmFC*, and *ccmFN*), three cytochrome *c* oxidase genes (*cox1*, *cox2*, and *cox3*), one protein transport subunit gene (*mttB*), one maturase gene (*matR*), and one cytochrome *b* gene (*cob*). The non-core genes are represented by one ribosomal protein large subunit gene (*rpl10*), nine ribosomal protein small subunit genes (*rps2*, *rps3*, *rps4*, *rps7*, *rps10*, *rps11*, *rps12*, *rps13*, and *rps19*), and two succinate dehydrogenase genes (*sdh3* and *sdh4*). Additionally, unlike all rRNA genes, which were single-copy, certain PCGs or tRNA genes presented in double copies. Among the three *Epimedium* species, *atp9*, *rps2*, and *trnP-UGG* were consistently present in two copies. The *rps13* gene was duplicated in *E. sagittatum* and *E. wushanense*, whereas *trnD-GUC* showed two copies in *E. pubescens* and *E. wushanense*. Additionally, *trnL-CAA* and *trnE-UUC* were duplicated in *E. pubescens* and *E. wushanense*, respectively.

**Table 2:** Gene composition in the mitogenome of three *Epimedium* species.

Group of genes	Name of genes
ATP synthase	*atp1*, *atp4*, *atp6*, *atp8*, *atp9*^1^ (×2)
NADH dehydrogenase	*nad1*, *nad2*, *nad3*, *nad4*, *nad4L*, *nad5*, *nad6*, *nad7*, *nad9*
Cytochrome *b*	*cob*
Cytochrome *c* biogenesis	*ccmB*, *ccmC*, *ccmFC*, *ccmFN*
Cytochrome *c* oxidase	*cox1*, *cox2*, *cox3*
Maturases	*matR*
Protein transport subunit	*mttB*
Ribosomal protein large subunit	*rpl10*
Ribosomal protein small subunit	*rps2*^1^ (×2), *rps3*, *rps4*, *rps7*, *rps10*, *rps11*, *rps12*, *rps13*^2^ (×2), *rps19*
Succinate dehydrogenase	*sdh3*, *sdh4*
Ribosome RNA	*rrn5*, *rrn18*, *rrn26*
Transfer RNA	*trnC-GCA*, *trnD-GUC*^3^ (×2), *trnE-UUC*^4^ (×2), *trnF-GAA*, *trnfM-CAU*, *trnG-GCC*, *trnH-GUG*, *trnI-CAU*, *trnK-UUU*, *trnL-CAA*^5^ (×2), *trnM-CAU*, *trnN-GUU*, *trnP-CGG*, *trnP-UGG*^1^ (×2), *trnQ-UUG*, *trnS-GCU*, *trnS-UGA*, *trnW-CCA*, *trnY-GUA*

“×2” represents the number of copies. For example, *atp9* had two copies.

^1^The genes that had two copies in the three *Epimedium* species.

^2^The genes that had two copies in *Epimedium sagittatum* and *Epimedium wushanense*.

^3^The genes that had two copies in *Epimedium pubescens* and *E. wushanense*.

^4^The genes that had two copies in *E. wushanense*.

^5^The genes that had two copies in *E. pubescens*.

### 3.2 Repeat element analysis

**Figure 3:**
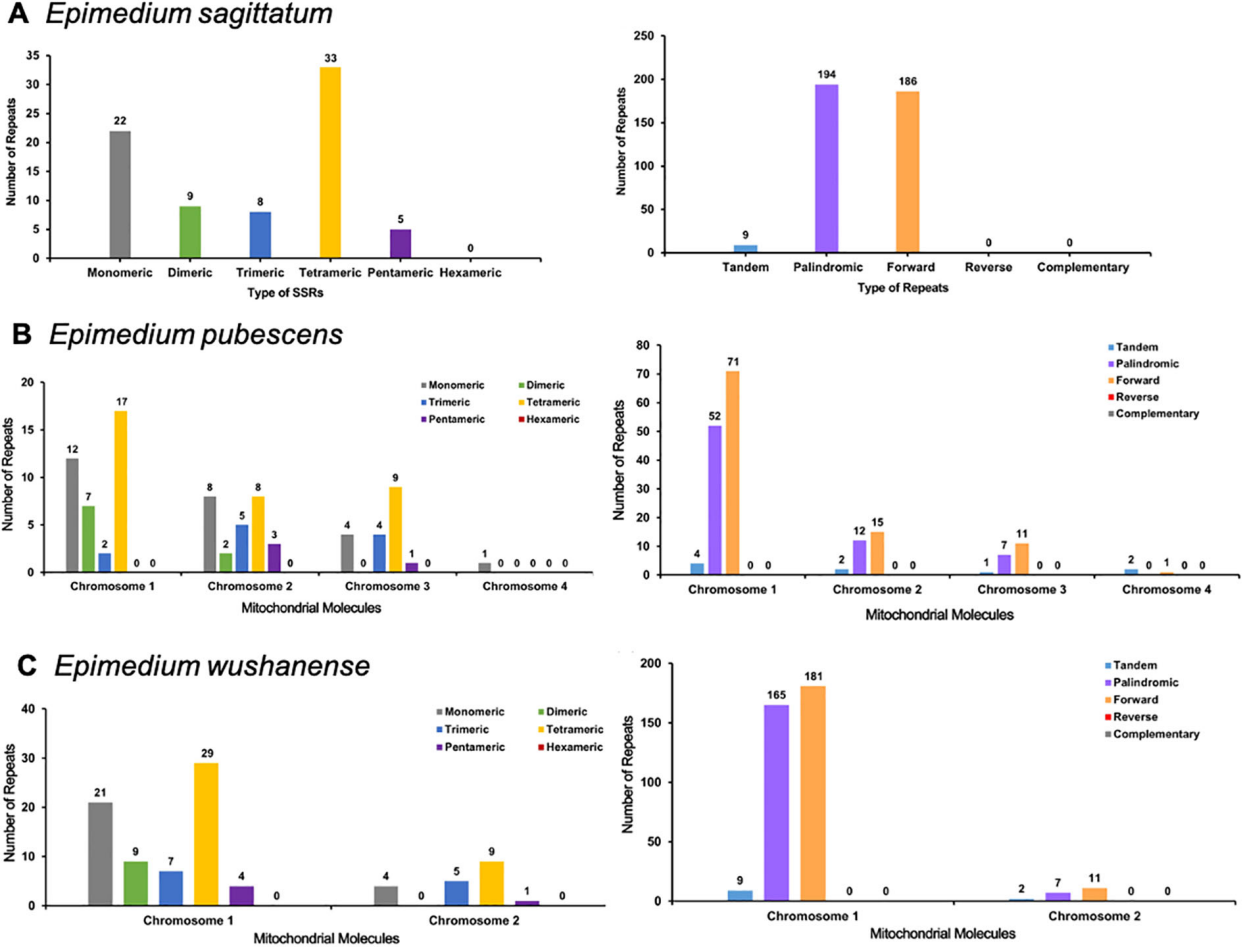
Analysis of repeat elements in the mitochondrial genome of **(A)***Epimedium sagittatum*, **(B)***Epimedium pubescens*, and **(C)***Epimedium wushanense*. Distribution and classification of repeat motifs based on SSR unit length (monomeric to hexameric) and structural types (tandem, palindromic, forward, reverse, and complementary repeats). SSR, simple sequence repeat.

A total of 77, 83, and 89 SSRs were identified in the *E. sagittatum*, *E. pubescens*, and *E. wushanense* mitogenomes, respectively (**Figures 3A–C**; **Supplementary Table S4**). Monomeric and tetrameric SSRs constituted the largest proportion of SSRs, accounting for 71.43%, 71.08%, and 70.79% in the *E. sagittatum*, *E. pubescens*, and *E. wushanense* mitogenomes, respectively. Notably, no hexameric repeats were found in the three *Epimedium* species mitogenomes. Furthermore, a total of nine, nine, and 11 tandem repeats were detected within the *E. sagittatum*, *E. pubescens*, and *E. wushanense* mitogenomes, respectively, displaying a variation in length ranging from 12 to 39 bp (**Supplementary Table S5**). Additionally, in the *E. pubescens* mitogenome, a total of four, two, one, and two tandem repeats, ranging from 15 to 32, 18 to 39, 12, and 29 bp, matched on chromosomes 1, 2, 3, and 4, respectively. Moreover, in the *E. wushanense* mitogenome, a total of nine and two tandem repeats, ranging from 15 to 39 and 12 to 25 bp, matched on chromosomes 1 and 2, respectively. In addition to these tandem repeats and SSRs, dispersed repeats are also prevalent throughout the genome, including palindromic repeats, forward repeats, reverse repeats, and complement repeats, and their function was observed to be of equivalent significance, as supported by previous studies. Here, a total of 380, 169, and 364 dispersed repeats were identified in *E. sagittatum*, *E. pubescens*, and *E. wushanense* mitogenomes, respectively, and each repeat was at over or equal to 30 bp in length (**Supplementary Table S6**). Of these dispersed repeats, 194 palindromic repeats and 186 forward repeats, 71 palindromic repeats and 98 forward repeats, and 172 palindromic repeats and 192 forward repeats were identified in the *E. sagittatum*, *E. pubescens*, and *E. wushanense* mitogenomes, respectively. No reverse repeats and complement repeats were detected in the three *Epimedium* species mitogenomes.

### 3.3 Analysis of relative synonymous codon usage

**Figure 4:**
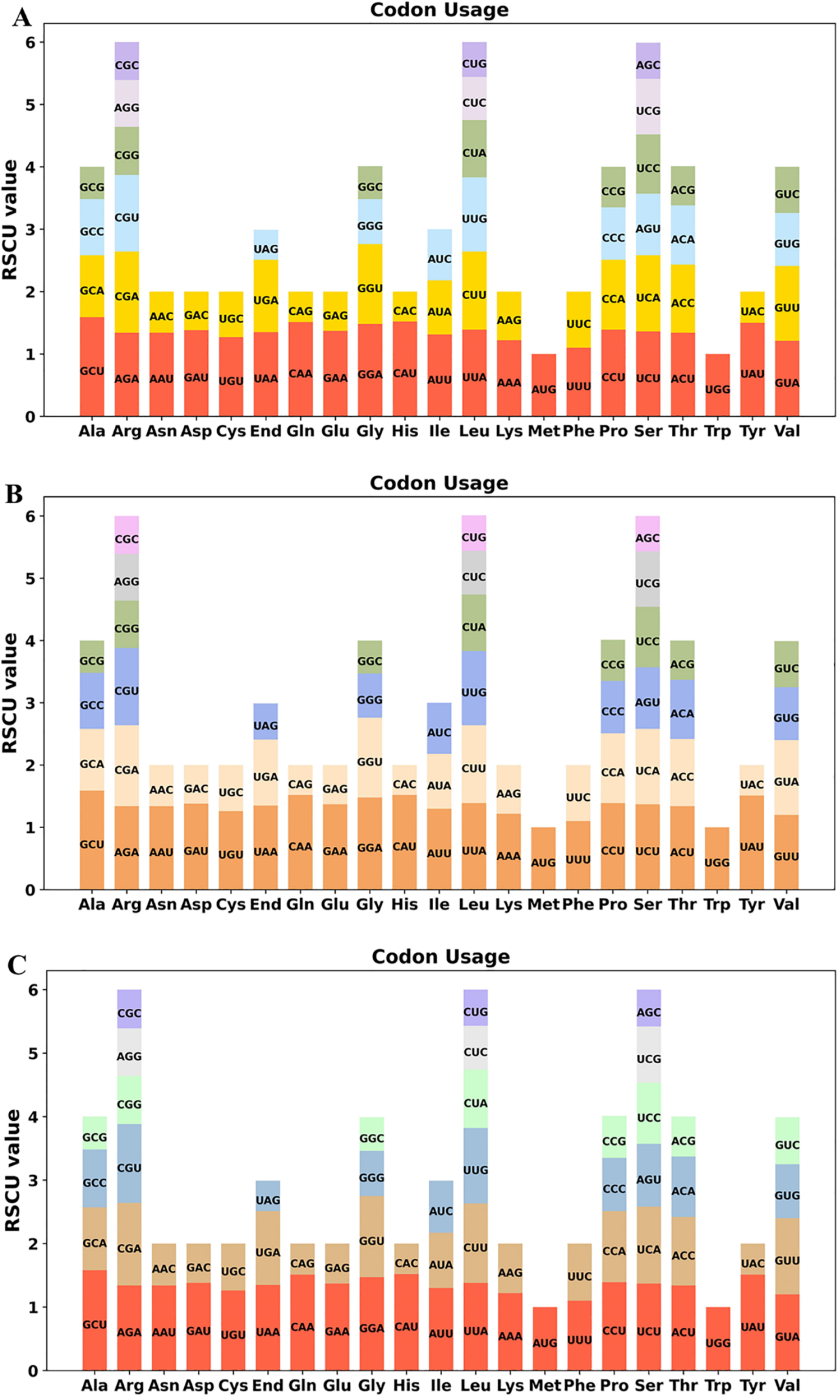
Relative synonymous codon usage (RSCU) in the mitochondrial protein-coding genes of three *Epimedium* species: **(A)***Epimedium sagittatum*, **(B)***Epimedium pubescens*, and **(C)***Epimedium wushanense*. The figures illustrate RSCU values for 36 unique mitochondrial protein-coding genes, representing codon usage patterns across 20 amino acids and stop codons.

In our study, 36 unique PCGs were conducted to analyze the codon usage patterns in the three *Epimedium* mitogenomes (**Supplementary Tables S7–S9**). RSCU values exceeding 1 signify a preference for specific codons, indicating a bias toward certain amino acids, whereas values less than 1 imply the opposite. These mitogenome PCGs exhibited a general preference for codon use except for the standard AUG (Met) and UGG (Trp). For instance, alanine (Ala) exhibited a comparable highest preference for the codon GCU, with an RSCU value ranging from 1.58 to 1.59. Additionally, most amino acids were represented by at least two different codons, whereas arginine, leucine, and serine each have six associated codons (**Figures 4A–C**).

### 3.4 RNA editing events

**Figure 5:**
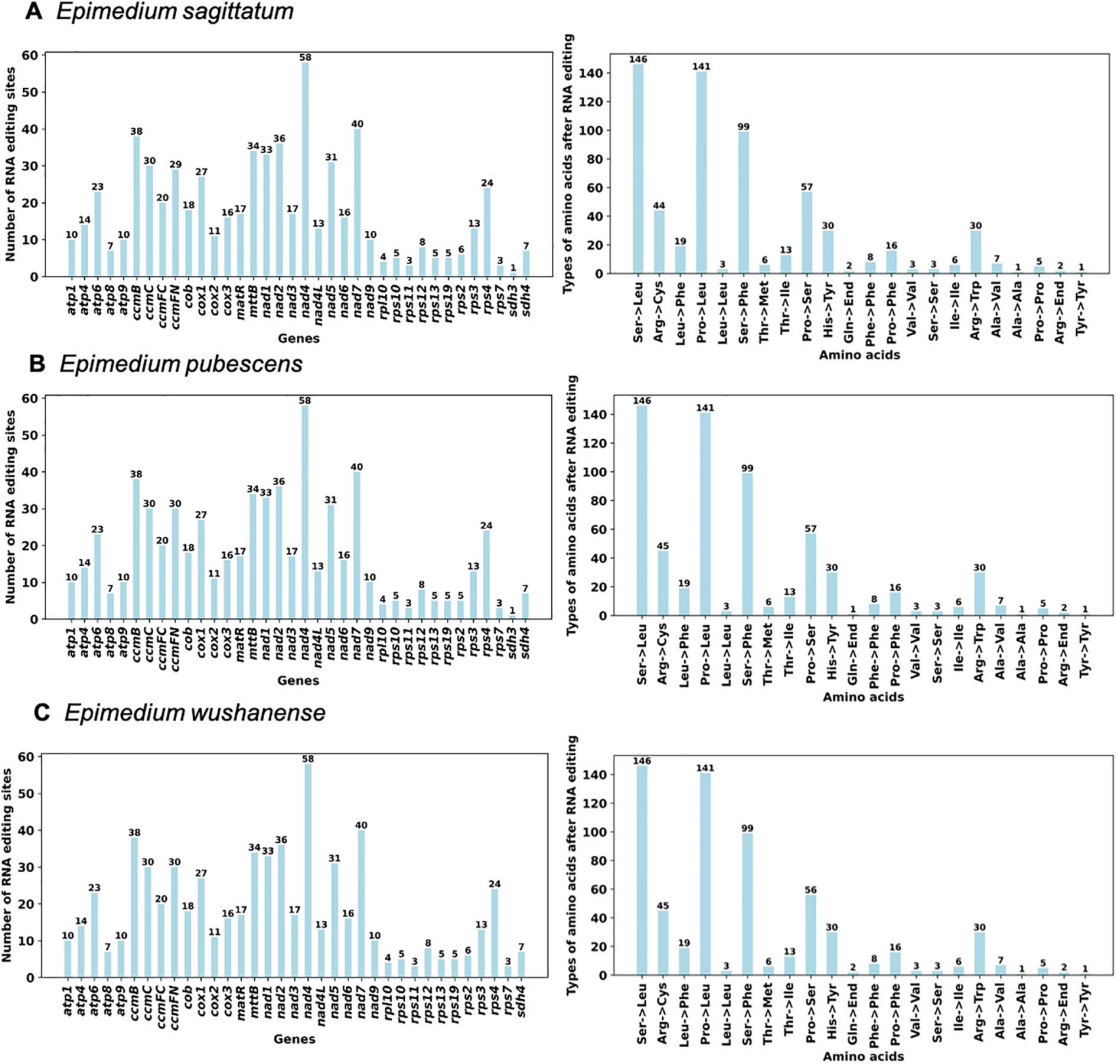
Predicted RNA editing sites based on protein-coding genes of **(A)***Epimedium sagittatum*, **(B)***Epimedium pubescens*, and **(C)***Epimedium wushanense*. The bar chart displays the number of predicted RNA editing sites of protein-coding genes in three *Epimedium* species. The x-axis represents different protein-coding genes, while the y-axis indicates the number of RNA editing sites for each gene. Each bar corresponds to the number of editing sites in a gene, visually representing the distribution of editing sites across the genes.

Previous studies have shown that RNA editing events are pivotal in plant growth and development (Small et al., 2020). RNA editing sites were predicted in the three *Epimedium* species (*E. sagittatum*, *E. pubescens*, and *E. wushanense*), with each species exhibiting a total of 642 editing sites. These sites were distributed across 36 unique PCGs in *E. sagittatum* and *E. pubescens* and across 35 PCGs in *E. wushanense* (**Figure 5**; **Supplementary Tables S10–12**). All identified editing events involved cytidine (C)-to-uridine (U) conversions. *nad4* harbored the highest number of editing sites (58), followed by *nad7* (40). Although the overall number of RNA editing sites was consistent across most PCGs in the three *Epimedium* species, some PCGs exhibited notable variations. For example, predicted RNA editing sites in *ccmFN* were detected in *E. sagittatum* (29), *E. pubescens* (30), and *E. wushanense* (30). The gene *rps2* had five predicted editing sites in *E. pubescens*, and the same number was detected in the other two species. For *sdh3*, one editing site was predicted in both *E. pubescens* and *E. sagittatum*, whereas no editing sites were detected in *E. wushanense*.

**Figure 6:**
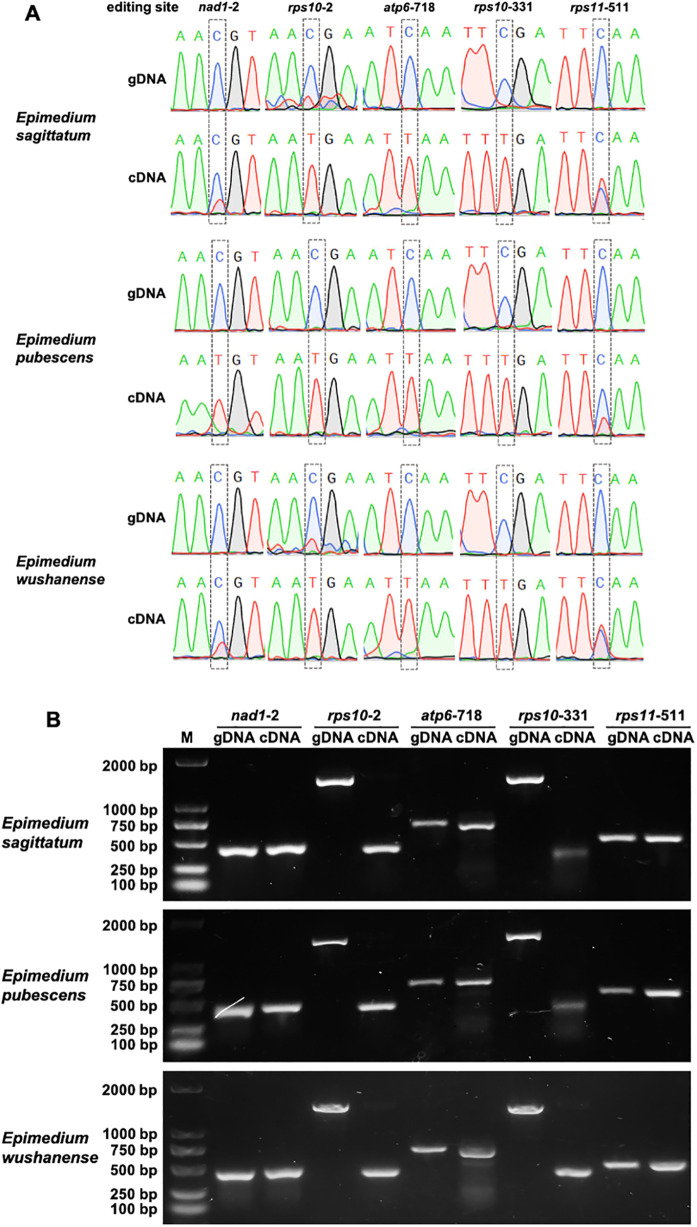
Validation of RNA editing sites in mitochondrial protein-coding genes of three *Epimedium* species. **(A)** PCR amplification of five editing sites (*nad1*-2, *rps10*-2, *atp6*-718, *rps10*-331, and *rps11*-511) from four mitochondrial protein-coding genes (*nad1*, *rps10*, *atp6*, and *rps11*) in genomic DNA (gDNA) and complementary DNA (cDNA) from three *Epimedium* species. **(B)** RNA editing validation at five sites (*nad1*-2, *rps10*-2, *atp6*-718, *rps10*-331, and *rps11*-511) in *Epimedium sagittatum*, *Epimedium pubescens*, and *Epimedium wushanense*. Sanger sequencing chromatograms of gDNA and cDNA show C-to-U conversions at the indicated sites. Dashed boxes highlight the edited positions.

To confirm the accuracy of predicted RNA editing events, five sites (*nad1*-2, *rps10*-2, *atp6*-718, *rps10*-331, and *rps11*-511) from four PCGs (*nad1*, *rps10*, *atp6*, and *rps11*) shared among the three species that generate start and stop codons were selected. For example, the following were predicted: one ACG (Thr) to AUG (Met) in the start codon of *nad1* and *rps10*, one CAA (Gln) to UAA (End) in *atp6* and *rps11*, and one CGA (Arg) to UGA (End) in *rps10*. The corresponding genomic DNA (gDNA) and complementary DNA (cDNA) were amplified using gene-specific primers, and the PCR products were subsequently sequenced via Sanger sequencing (**Figure 6A**). The sequencing results revealed C-to-U conversions at positions *nad1–*2 and *rps11–*511 in the cDNA compared to the gDNA, although the extent of editing varied slightly among the three *Epimedium* species, indicating species-specific RNA editing at these sites (**Figure 6B**). Overall, Sanger sequencing confirmed RNA editing at several positions in cDNA, supporting the occurrence of site- and species-specific RNA editing events within the genus *Epimedium*.

### 3.5 Intracellular gene transfer between chloroplast and mitochondrial genomes

**Figure 7:**
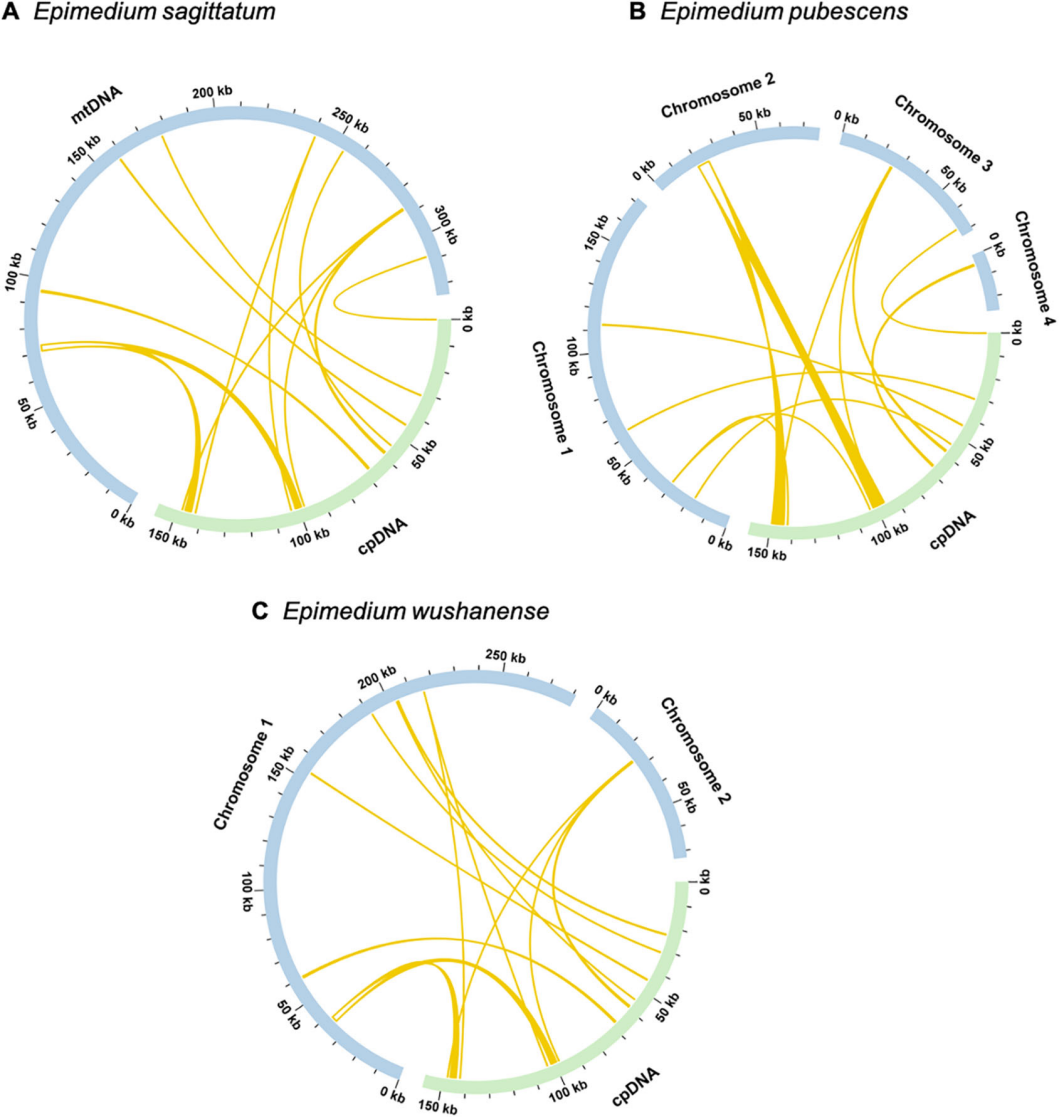
Homologous analysis between mitogenome and chloroplast genome of **(A)***Epimedium sagittatum*, **(B)***Epimedium pubescens*, and **(C)***Epimedium wushanense*. The blue arcs represent mitogenome, and the green arcs represent chloroplast genome. The yellow arcs inside the circle represent the homologous regions between mitochondrial and chloroplast genomes.

Mitochondrial plastid DNAs (MTPTs) refer to specific DNA fragments of chloroplast origin that exist in the mitogenome. For the three *Epimedium* species, sequence alignment exhibited nine homologous fragments between the chloroplast and mitochondrial genomes (**Supplementary Tables S13–15**). For *Epimedium sagittatum*, the total length of the nine homologous fragments spanned 4,394 bp (1.35% of the mitogenome), with MTPT1 being the largest at 2,400 bp in size, making it the most substantial fragment among the identified homologous sequences (**Figure 7A**). Subsequent annotation of these sequences unveiled the presence of seven complete genes, comprising two complete PCGs and five tRNA genes. The PCGs identified were *petG* and *rps7*. The tRNA genes included *trnD-GUC*, *trnH-GUG*, *trnM-CAU*, *trnP-UGG*, and *trnW-CCA*. In *Epimedium pubescens*, the total length of the nine homologous fragments spanned 7,505 bp (2.17% of the mitogenome), with MTPT5 as the largest at 5,505 bp, representing the most substantial fragment among the identified homologous sequences (**Figure 7B**). Further examination of these sequences showed that nine complete genes were present, comprising three complete PCGs and six tRNA genes. The PCGs identified were *petG*, *rps7*, and *ndhB*. The tRNA genes included *trnD-GUC*, *trnH-GUG*, *trnM-CAU*, *trnP-UGG*, *trnW-CCA*, and *trnL-CAA*. In *Epimedium wushanense*, these transfer fragments spanned 4,706 bp, comprising 1.33% of the mitogenome. Among these transfer fragments, MTPT1 was the largest, measuring 2,494 bp in size and representing the most substantial fragment among the identified homologous sequences (**Figure 7C**). Further annotation of these sequences showed that six complete genes were present, comprising two complete PCGs and four tRNA genes. The PCGs identified were *petG* and *rps7*, while the detected tRNA genes included *trnD-GUC*, *trnM-CAU*, *trnP-UGG*, and *trnW-CCA*.

### 3.6 Collinear analysis

**Figure 8:**
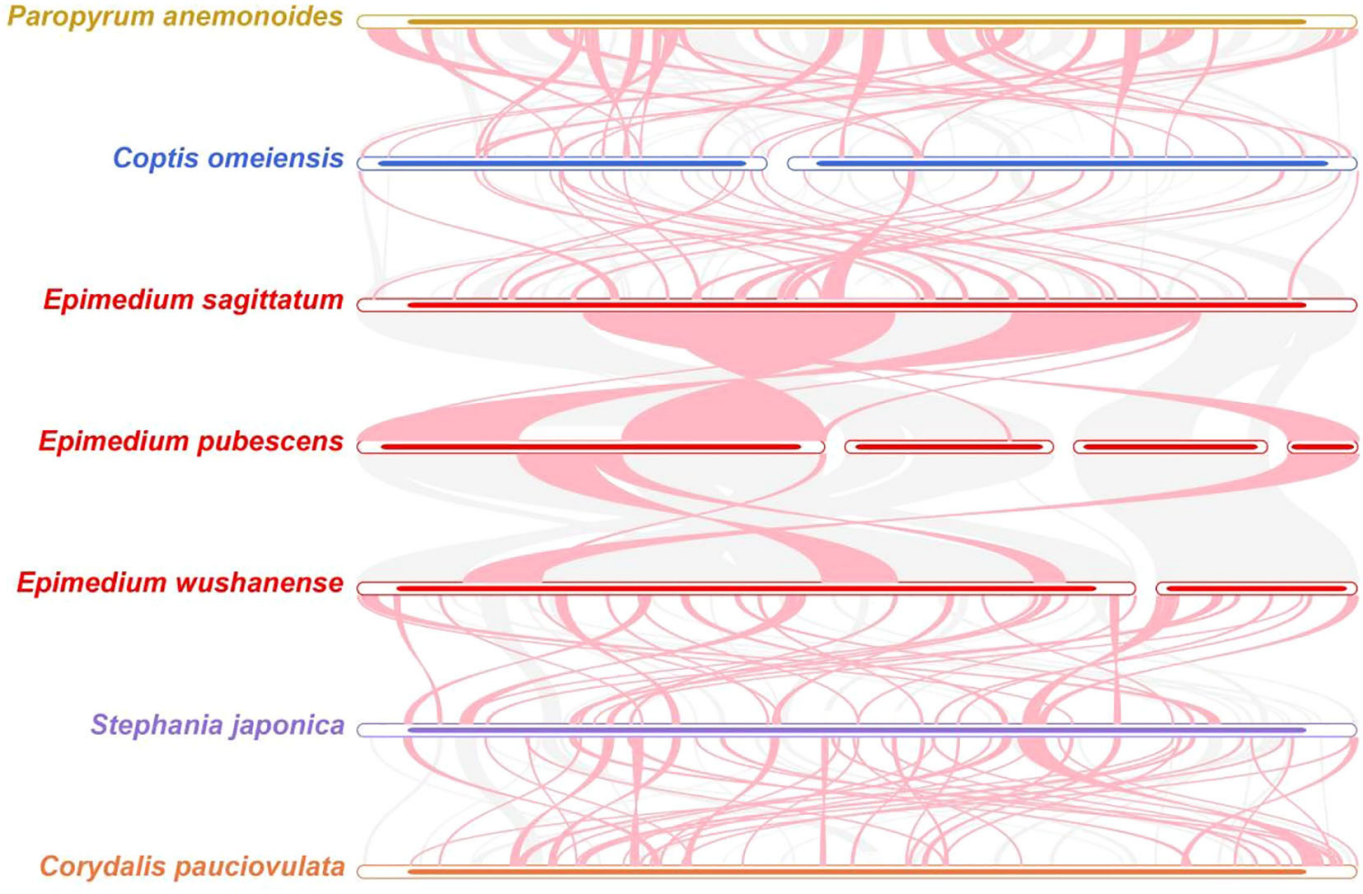
Collinear analysis of seven species in Ranunculales. The pink curve blocks indicate regions with inversion events, the gray ones represent homologous regions, and areas lacking collinear blocks were unique to each species.

To further clarify the conservatism of mitogenome evolution in three *Epimedium* species and four other species (*Paropyrum anemonoides*, *C. omeiensis*, *Stephania japonica*, and *C. pauciovulata*) (**Figure 8**; **Supplementary Table S16**), collinearity analysis revealed a high degree of synteny among the three *Epimedium* species, with long conserved blocks—for example, chromosome 4 of *E. pubescens* showed substantial homologous regions in both *E. sagittatum* and *E. wushanense*, indicating its conservation during *Epimedium* mitogenome evolution. However, many homologous collinear blocks were relatively short, and some were missing in the compared genomes, suggesting the presence of unique regions within each mitogenome. Furthermore, the order of these collinear blocks varied among the seven species analyzed, reflecting extensive gene rearrangements. Additionally, numerous inverted regions were detected, implying a lower conservation of chromosomal structure across the mitogenomes.

### 3.7 Phylogenetic analysis

**Figure 9:**
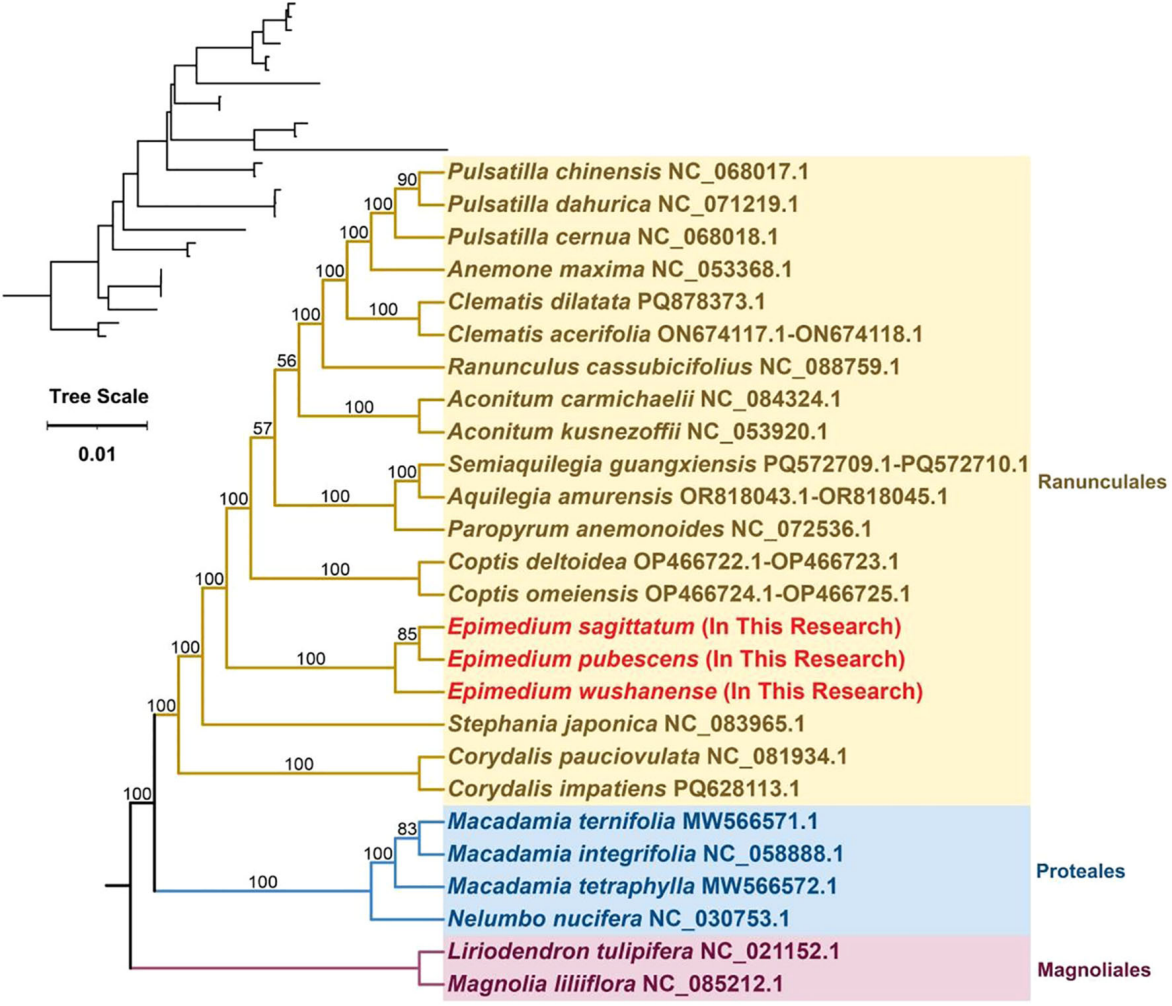
Phylogenetic relationships of three *Epimedium* species (*Epimedium sagittatum*, *Epimedium pubescens*, and *Epimedium wushanense*) with other 17 species in Ranunculales and four species in Proteales; two species from Magnoliales were used as outgroup. The phylogenetic tree was constructed based on concatenated sequences of conserved mitochondrial protein-coding genes from 26 species using the maximum likelihood (ML) method. Bootstrap support values from 1,000 replicates are shown at each node. The original tree with branch lengths is displayed in the upper left corner. These 29 PCGs were *atp1*, *atp4*, *atp6*, *atp8*, *atp9*, *ccmB*, *ccmC*, *ccmFC*, *ccmFN*, *cob*, *cox1*, *cox2*, *cox3*, *matR*, *mttB*, *nad1*, *nad2*, *nad3*, *nad4*, *nad4L*, *nad5*, *nad6*, *nad7*, *rpl10*, *rps3*, *rps4*, *rps7*, *rps12*, and *rps13*.

To better elucidate the phylogenetic relationships of the *Epimedium* species with closely related species, a maximum likelihood (ML) phylogenetic tree was reconstructed based on 29 conserved PCGs shared among 26 species across three orders—Ranunculales, Proteales, and Magnoliales—with *Liriodendron tulipifera* (NC_021152.1) and *Magnolia liliiflora* (NC_085212.0) serving as outgroups (**Figure 9**). Phylogenetic analysis revealed that the three *Epimedium* species within the Pandanales order clustered clade with 100% bootstrap support. *E. sagittatum* and *E. pubescens* clustered together, indicating a close phylogenetic relationship, and this clade formed a sister group with *E. wushanense*. The phylogenetic tree constructed using mitochondrial PCGs effectively distinguishes *Epimedium* species from other taxa and exhibits a well-resolved topology that is consistent with the latest classification by the Angiosperm Phylogeny Group (APG), supporting the reliability of mitochondrial PCGs for phylogenetic inference in plants.

## 4 Discussion

Both the mitochondrial and chloroplast genomes are essential for energy production and cellular metabolism in plants, while they function in different aspects of cellular activity. The chloroplast genome of *E. koreanum* was first reported in 2016 (Lee et al., 2016), and subsequently, the chloroplast genomes of other *Epimedium* species were published, including *E. acuminatum*, *E. lishihchenii*, *E. pseudowushanense*, and *E. wushanense* (Sun et al., 2016b; Guo et al., 2019; Lai et al., 2022). These studies provided insights into the *Epimedium* chloroplast and the phylogenetic relationships of *Epimedium* species. However, there are no complete mitogenomes of *Epimedium* yet available in the public databases. The available data remain insufficient to clarify phylogenetic relationships across the genus, requiring further investigation from other genetic approaches. In our study, the complete mitogenome of three *Epimedium* (*E. sagittatum*, *E. pubescens*, and *E. wushanense*) species had been assembled and characterized. Our results showed that the three *Epimedium* mitogenomes displayed wide differences in structure. A configuration consisting of multiple branched molecules was observed in the *E. pubescens* (three circular chromosomes and one linear chromosome) and *E. wushanense* (two circular chromosomes) mitogenomes, rather than a single circular chromosome in the *E. sagittatum* mitogenome. In the graphical assembly results, two and four contigs in *E. sagittatum* and *E. wushanense*, respectively, were identified as repetitive sequences. These large repeats are known to mediate homologous recombination (Yang et al., 2022b), driving frequent genomic rearrangements and giving rise to the dynamic and multiconfigurational nature of plant mitogenomes. A similar structural diversity has also been reported in *Broussonetia* spp. and *Saccharum* spp., where different species within each genus exhibited considerable structure variation in the mitogenome (Lai et al., 2022; Li et al., 2024c). Additionally, *A. biserrata*, *Allium cepa*, and *Silene conica* also displayed complex multi-chromosomal structures (Alverson et al., 2011; Sloan et al., 2012; Wang et al., 2024).

During the evolutionary process in land plants, the GC content is vital for determining the amino acid composition of protein groups (Wang, 2020). The mitogenomes of the three *Epimedium* (*E. sagittatum*, *E. pubescens*, and *E. wushanense*) exhibited similar CG contents of 46.91%, 46.52%, and 46.59%, respectively, comparable to other plant species such as *Punica granatum* (46.09%) (Lu et al., 2023) and *Corydalis saxicola* (46.50%) (Li et al., 2024a). Significantly, this exceeds the GC content of the *Epimedium* chloroplast genome (38.8%) (Guo et al., 2019), suggesting that the GC content in angiosperm mitogenomes remains relatively stable throughout evolution.

Repetitive sequences, including tandem repeats, dispersed repeats, and SSRs, are widespread in organelle genomes and play crucial roles in shaping mitogenome architecture through rearrangements, duplications, and recombination events. For instance, in the mitogenomes of *Prunus salicina*, three pairs of repetitive sequences promoted genomic recombination, resulting in eight and seven distinct conformations (Fang et al., 2021). In this study, multiple types of repetitive sequences were abundantly identified in the three *Epimedium* mitogenomes. Specifically, 77, 83, and 89 SSRs were detected in the mitogenomes of *E. sagittatum*, *E. pubescens*, and *E. wushanense*, respectively, with monomeric and tetrameric motifs being the most prevalent. Notably, no hexameric repeats were found in the three *Epimedium* species mitogenomes. The findings are consistent with the findings in the chloroplast genome of *Epimedium* (Guo et al., 2019). Additionally, dispersed repeats and tandem repeats also showed variations in their distribution among different *Epimedium* species. A total of nine, nine, and 11 tandem repeats, displaying a variation in length ranging from 12 to 39 bp, and 380, 169, and 364 dispersed repeats were detected within the *E. sagittatum*, *E. pubescens*, and *E. wushanense* mitogenomes, respectively. The abundance of repetitive sequences in the mitogenome is markedly higher than in the chloroplast genome, reflecting a characteristic feature of plant mitochondria that contributes to their more complex genomic architecture. The mitogenome of *E. wushanense* contained a greater abundance and diversity of repetitive sequences than those of *E. sagittatum* and *E. pubescens*. This elevated repeat content reflects its enhanced recombination potential and structural complexity, resulting in a more dynamic mitochondrial genome organization. Although we have confirmed the existence of these structural variations, their specific roles within the genome still need to be further explored.

Codon usage bias, an important evolutionary feature, has been observed in both prokaryotic and eukaryotic organisms. In the mitogenomes of the three *Epimedium* species, we detected a strong preference for A/U-ending codons at the third codon position. Similar findings have been reported in other species, including *Elaeagnus* (Li et al., 2023a), *Cucumis sativus* (Niu et al., 2021), and *Camellia duntsa* (Li et al., 2023b), which suggests that A/U-ending codon bias may be an inherent characteristic of organelle genomes. However, other species, such as *S. tuberosa* (Xu et al., 2025), *A. biserrata* (Wang et al., 2024), and *Mangifera persiciformis* (Niu et al., 2022), tend to favor A/T-ending codons in the third position. These results revealed that codon usage patterns in plant mitogenomes had species-specific variations.

Plant mitogenomes exhibit a high frequency of RNA editing events, which result in amino acid changes via insertions, deletions, and substitutions, thereby leading to substantial genetic diversity (Sun et al., 2016a). The number of RNA editing sites in land plant mitogenomes can exhibit substantial variation, ranging from *Marchantia polymorpha* (0 sites) to *Selaginella moellendorffii* (2,152 sites) (Zhang et al., 2020b). In the present study, 642 sites were detected in *E. sagittatum*, *E. pubescens*, and *E. wushanense* across 36 unique PCGs (across 35 PCGs in *E. wushanense*), all involving C-to-U transitions. This observation is consistent with findings from prior studies, underscoring the substantial influence of RNA editing on the functional dynamics of mitochondrial genes. Additionally, in the three *Epimedium* species, one RNA editing site was detected in the *sdh3* gene in the *E. sagittatum* and *E. pubescens* mitogenomes, while no RNA editing sites were detected in the *sdh3* gene in the *E. wushanense* mitogenome, suggesting a certain variability in RNA editing site distribution among different species. Sanger sequencing confirmed several predicted editing events, including modifications that restore start and stop codons, reinforcing the functional significance of these edits. The observed variation in editing efficiency among species further suggests a layer of species-specific post-transcriptional regulation in *Epimedium* mitochondria.

Intracellular horizontal gene transfer (IHGT) denotes the transfer of genetic sequences among the mitogenome, plastome, and nuclear genome. Among these organellar genomes, the most frequent direction is from plastome to mitogenome. The mitogenome of *Salvia miltiorrhiza* (Yang et al., 2022a) harbors chloroplast-derived gene fragments, serving as direct evidence for the transfer of DNA segments from chloroplasts to mitochondria. Generally, plant mitogenomes contain approximately 0.56% (*M. polymorpha*) to 10.85% (*Phoenix dactylifera*) of plastid-derived sequences (Zhao et al., 2019). In this study, MTPTs in the three *Epimedium* species were analyzed, and it was found that *E. sagittatum* (4,394 bp, 1.35%), *E. pubescens* (7,505 bp, 2.17%), and *E. wushanense* (4,706 bp, 1.33%) integrated nine MTPTs. The minimal heterogeneity in MTPT length distribution among these species suggests that plastid-derived integrations contribute only marginally to mitogenome expansion, reflecting a relatively low proportion of chloroplast-to-mitochondrion DNA transfer in *Epimedium*. A total of nine MTPTs were identified in each of the three *Epimedium* mitogenomes. Among them, several genes, including the protein-coding genes (*petG*, *rps7*, and *ndhB*) and the tRNA genes (*trnD*, *trnH*, *trnM*, *trnP*, and *trnW*), were completely transferred from the chloroplast genome. The plastid-derived tRNA genes may serve a compensatory role for lost mitochondrial tRNAs, thus maintaining essential functions required for mitochondrial translation, such as amino acid transfer (Warren et al., 2021). These findings not only deepen our understanding of plant mitogenome dynamics but also offer valuable insights into plant evolutionary processes and adaptive mechanisms. However, more analyses involving a broader range of *Epimedium* species are required to establish a more definitive conclusion.

Mitogenomes are less frequently employed in phylogenetic analyses of higher plants than plastid and nuclear genomes, primarily due to their relatively low mutation rates, frequent genome rearrangements, and the incorporation of foreign sequences. In this study, we constructed a phylogenetic tree based on 29 conserved PCGs from the mitogenomes of 26 species across three orders, which resolved well-supported relationships within Pandanales and were largely consistent with the APG IV system (Chase et al., 2016; Zeb et al., 2025). Notably, comparison with related families revealed that the mitogenomic data provide complementary phylogenetic signals, clarifying the placement of *Epimedium* within the Berberidaceae. These findings highlight the evolutionary significance of mitogenome variation, including structural rearrangements and gene content, and demonstrate its potential to refine taxonomic classification. Expanding mitogenomic sampling in future studies will further illuminate the evolutionary dynamics and phylogenetic relationships across Pandanales.

## 5 Conclusions

In this study, three complete *Epimedium* mitogenomes were assembled and characterized, revealing remarkable structural variation, abundant RNA editing sites, and evidence of mitochondrial–plastid DNA transfer. Phylogenetic analysis resolved the evolutionary relationships among *E. sagittatum*, *E. pubescens*, and *E. wushanense*, providing new genomic perspectives on species differentiation within the genus. These findings enrich mitochondrial genomic resources for *Epimedium* and offer valuable references for comparative and evolutionary studies in the Berberidaceae. Beyond expanding taxonomic and genomic knowledge, this work establishes a foundation for future research on mitochondrial genome evolution, inter-organellar communication, and the molecular mechanisms underlying plant adaptation and diversification.

## Sample collection

In compliance with ethical standards, freshly collected and cultivated specimens of three Epimedium species were obtained from the Dazhou Academy of Agricultural Sciences in Dazhou, China. Experimental research and plant material collection complied with all relevant institutional, national, and international guidelines.

## Data Availability

The datasets presented in this study can be found in online repositories. The names of the repository/repositories and accession number(s) can be found in the article/**Supplementary Material**.
