# Supplementary figures and images for: Comparative analysis of mitochondrial genomes of Epimedium L. reveals heterogeneity in structure, synteny, intercellular gene transfer, and RNA editing
Source: Front Plant Sci. 2025 Nov 24;16:1701895. doi: 10.3389/fpls.2025.1701895 (PMC12682791; doi:10.3389/fpls.2025.1701895)

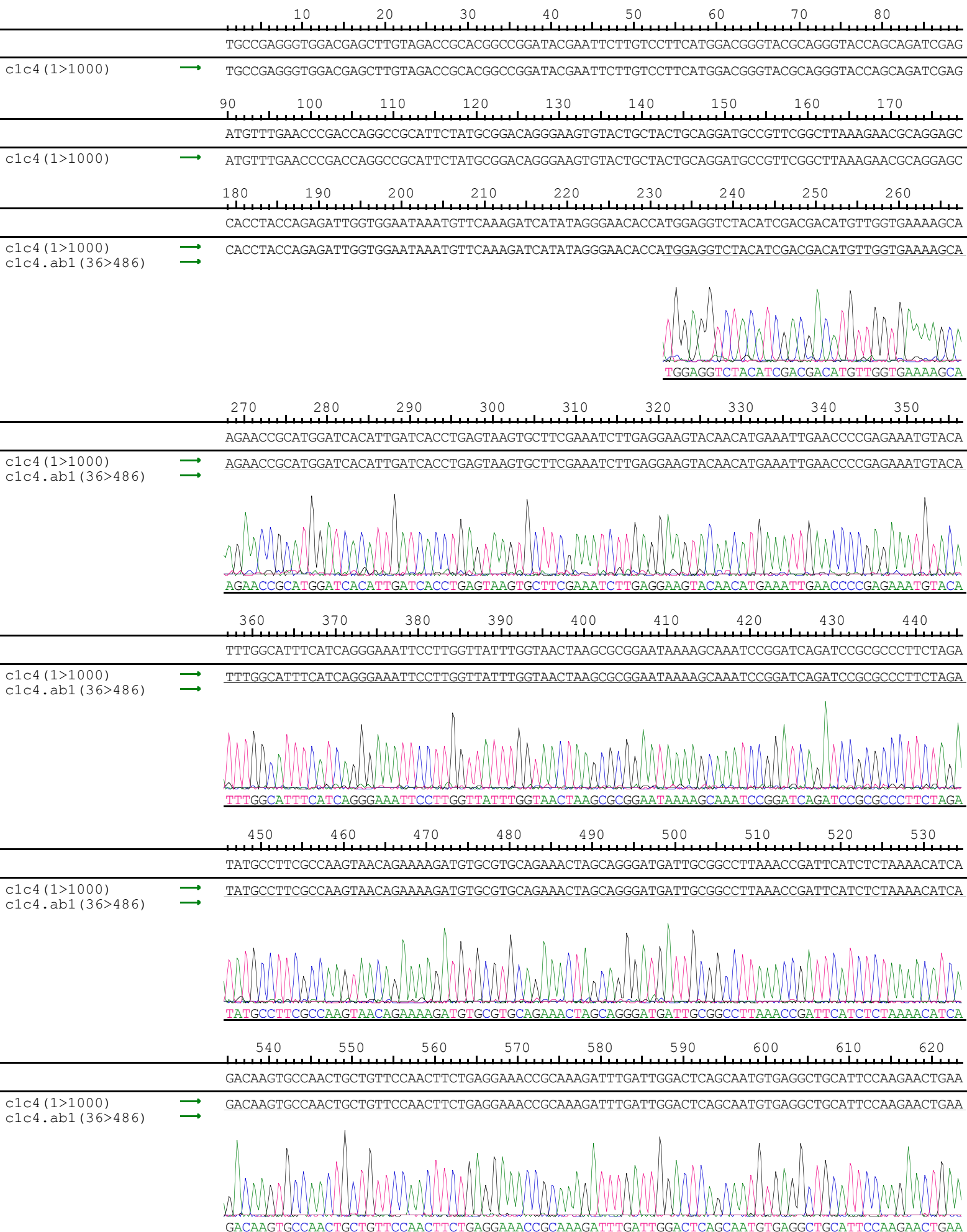

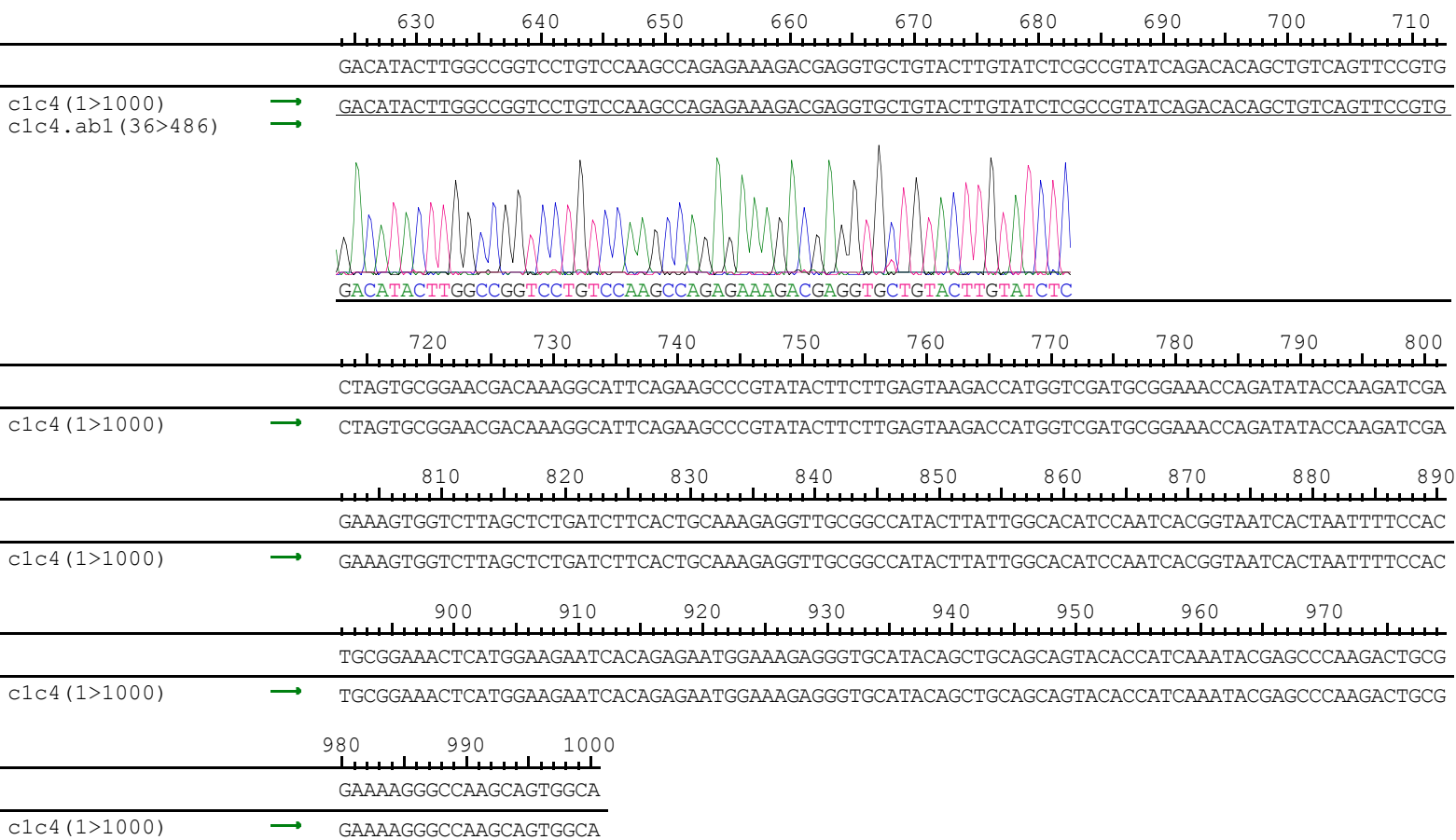

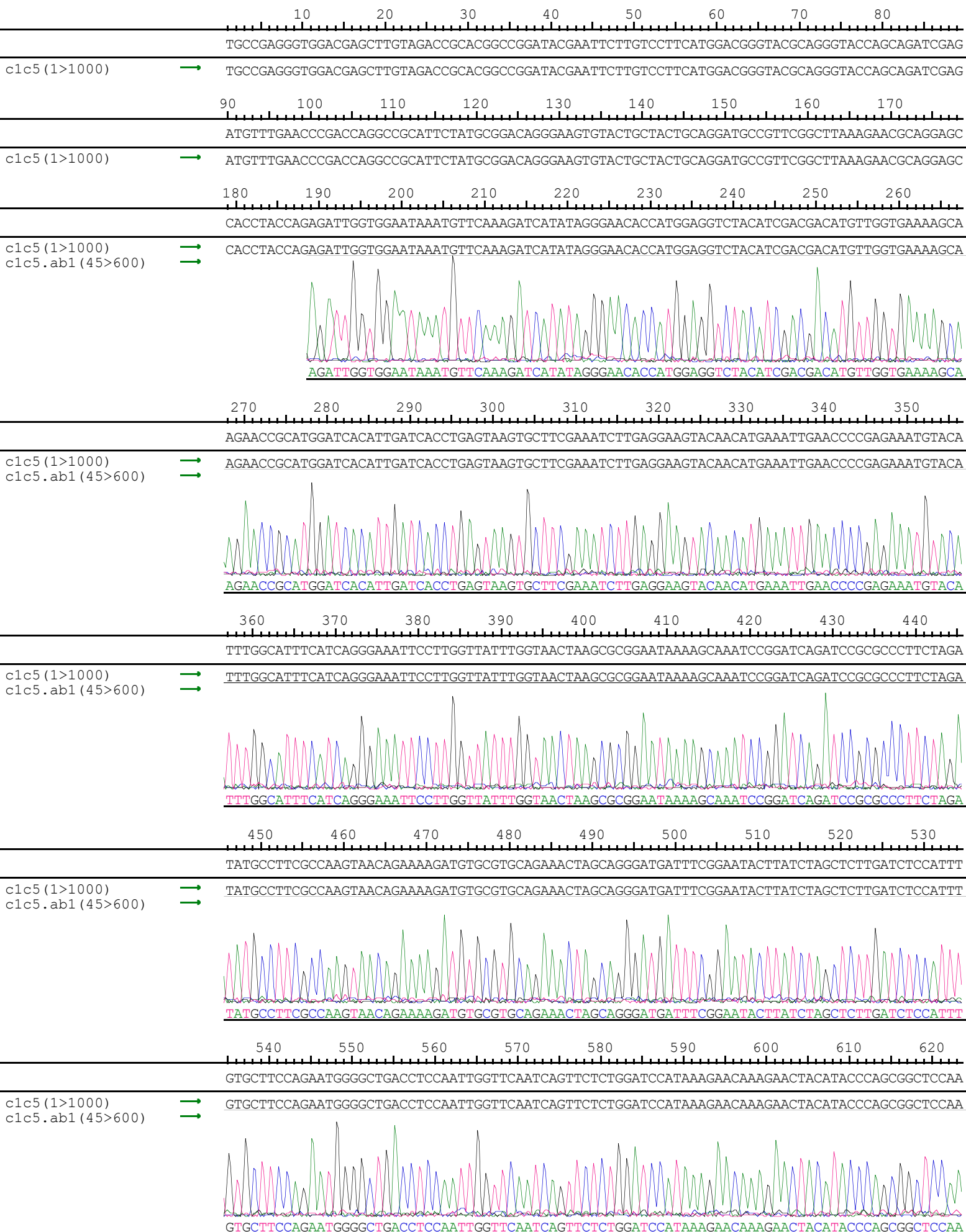

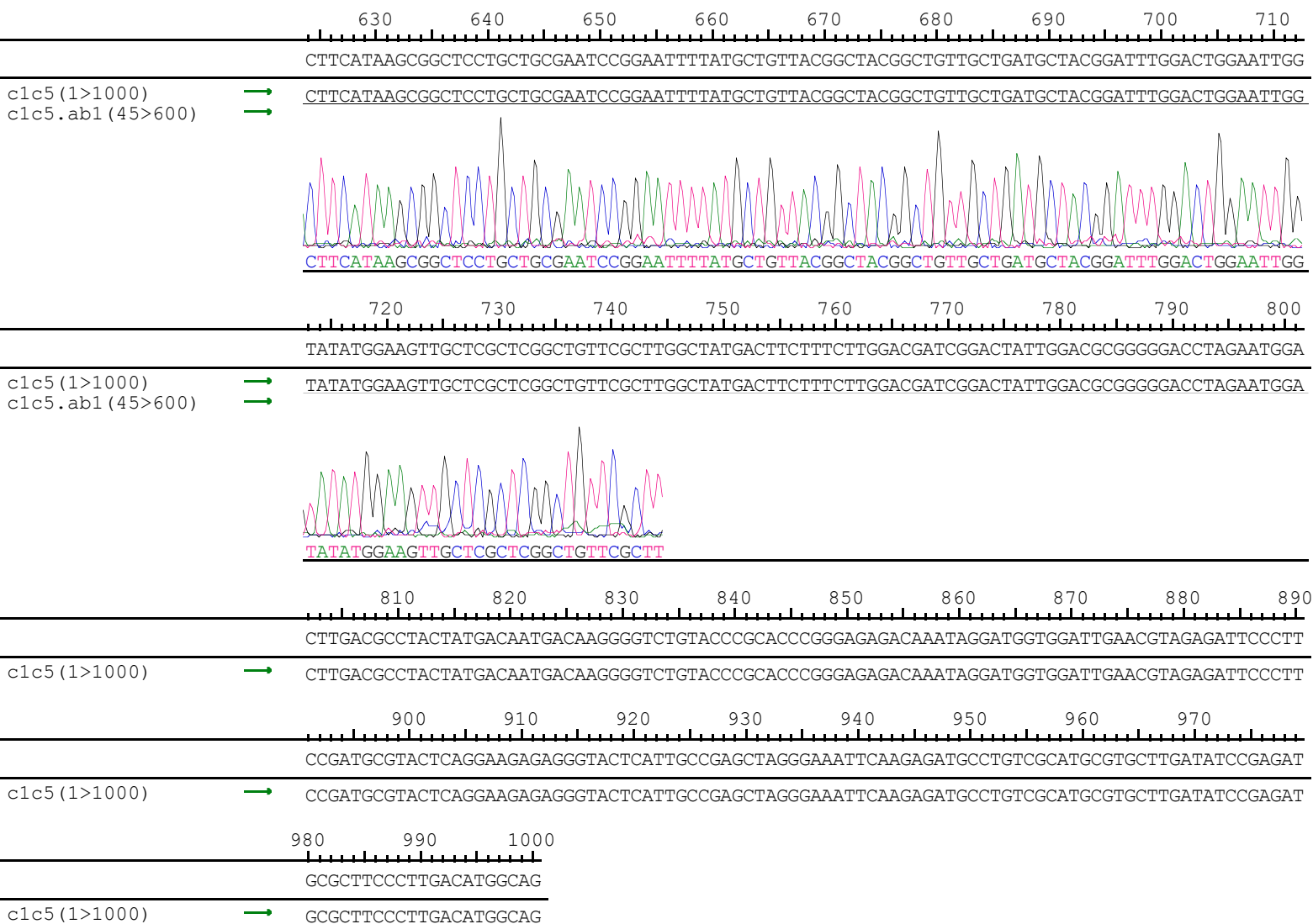

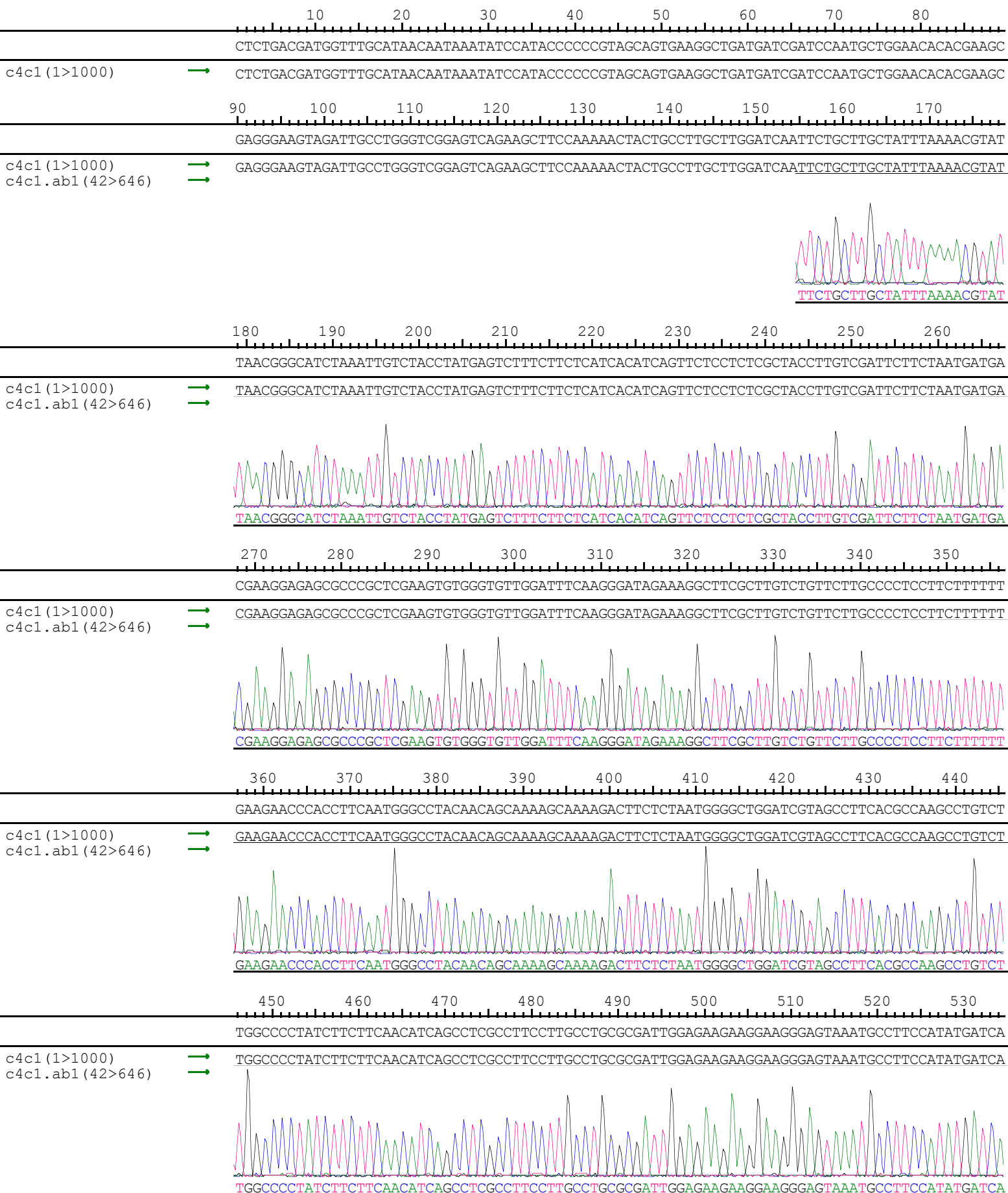

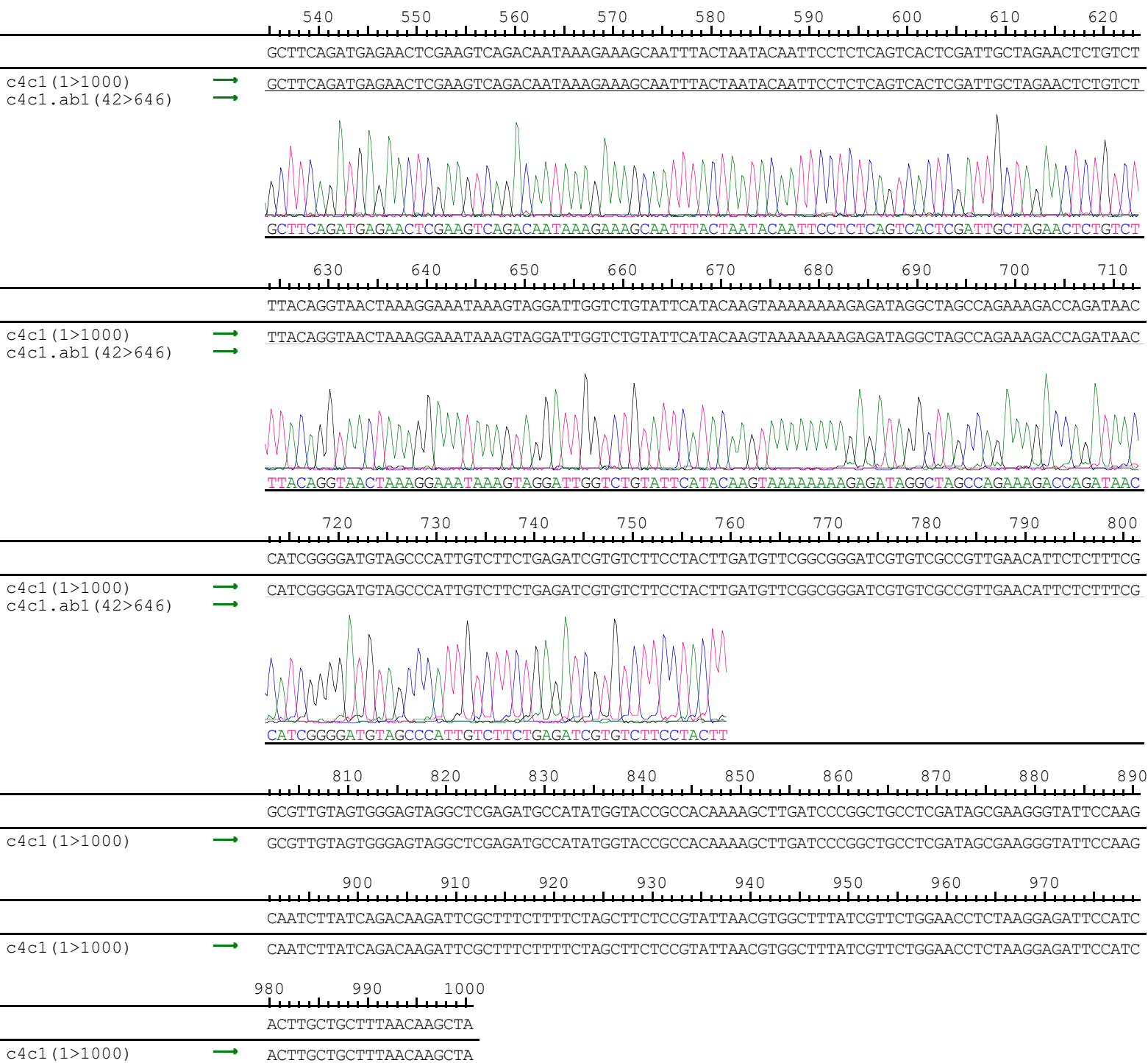

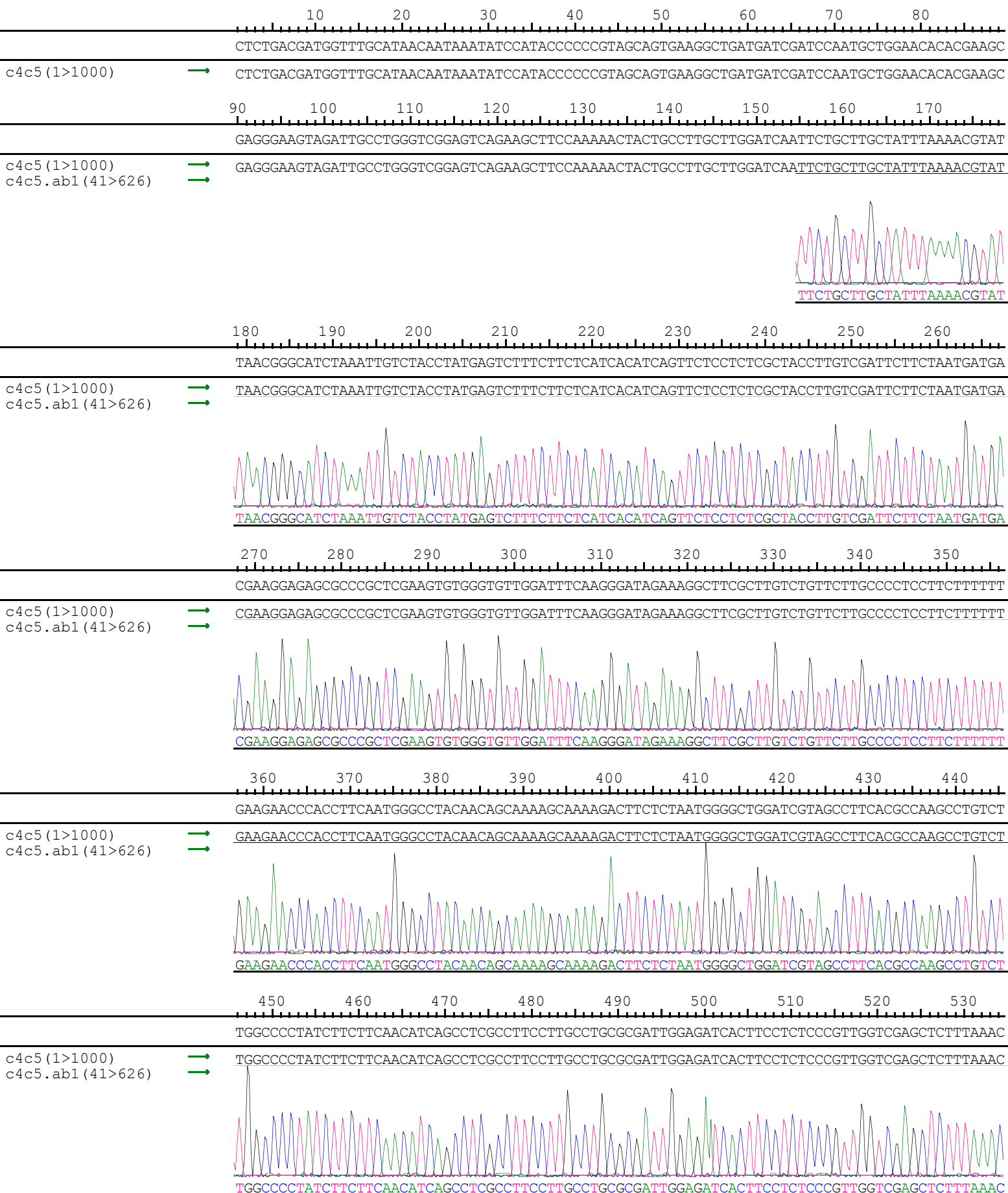

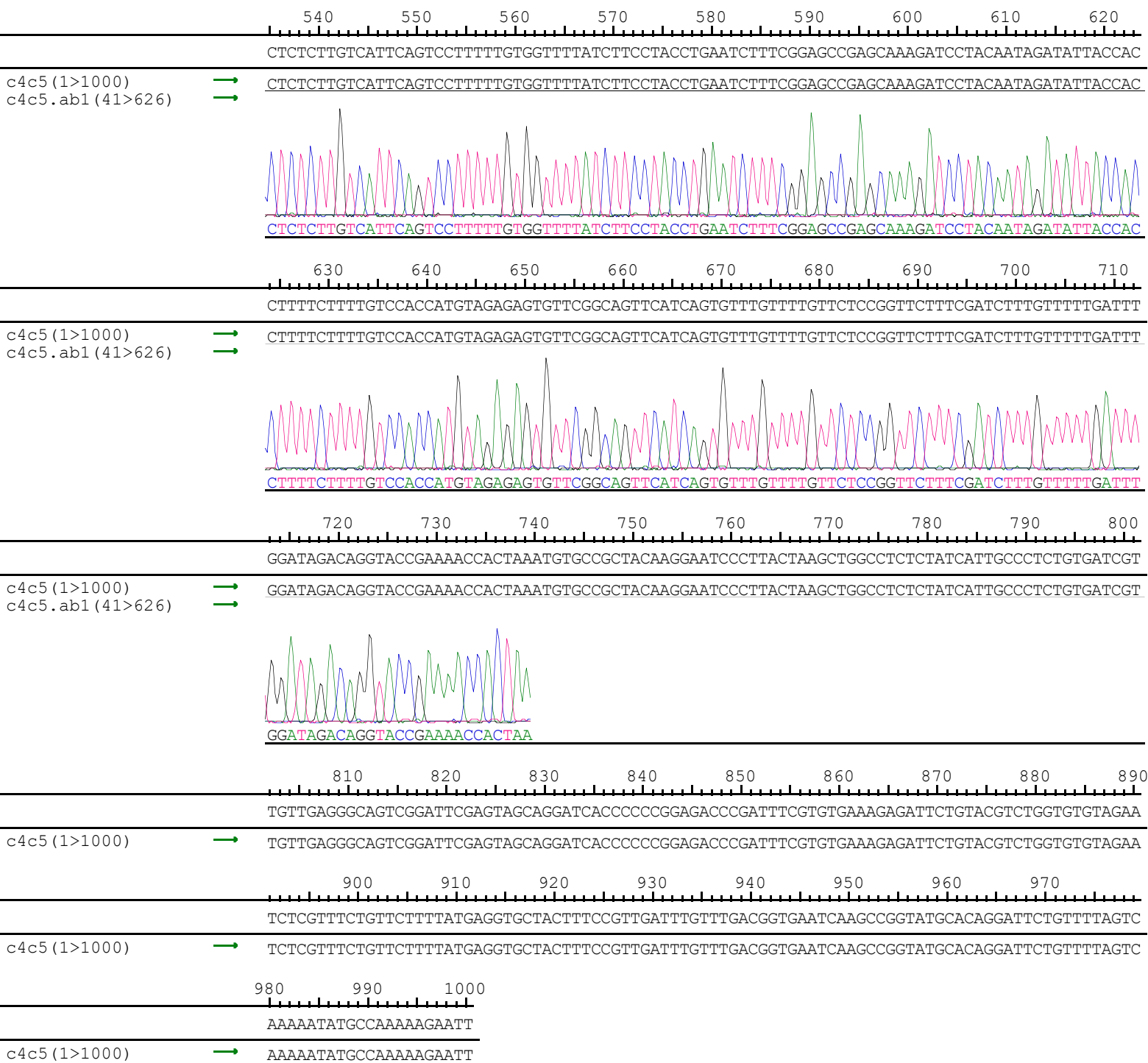

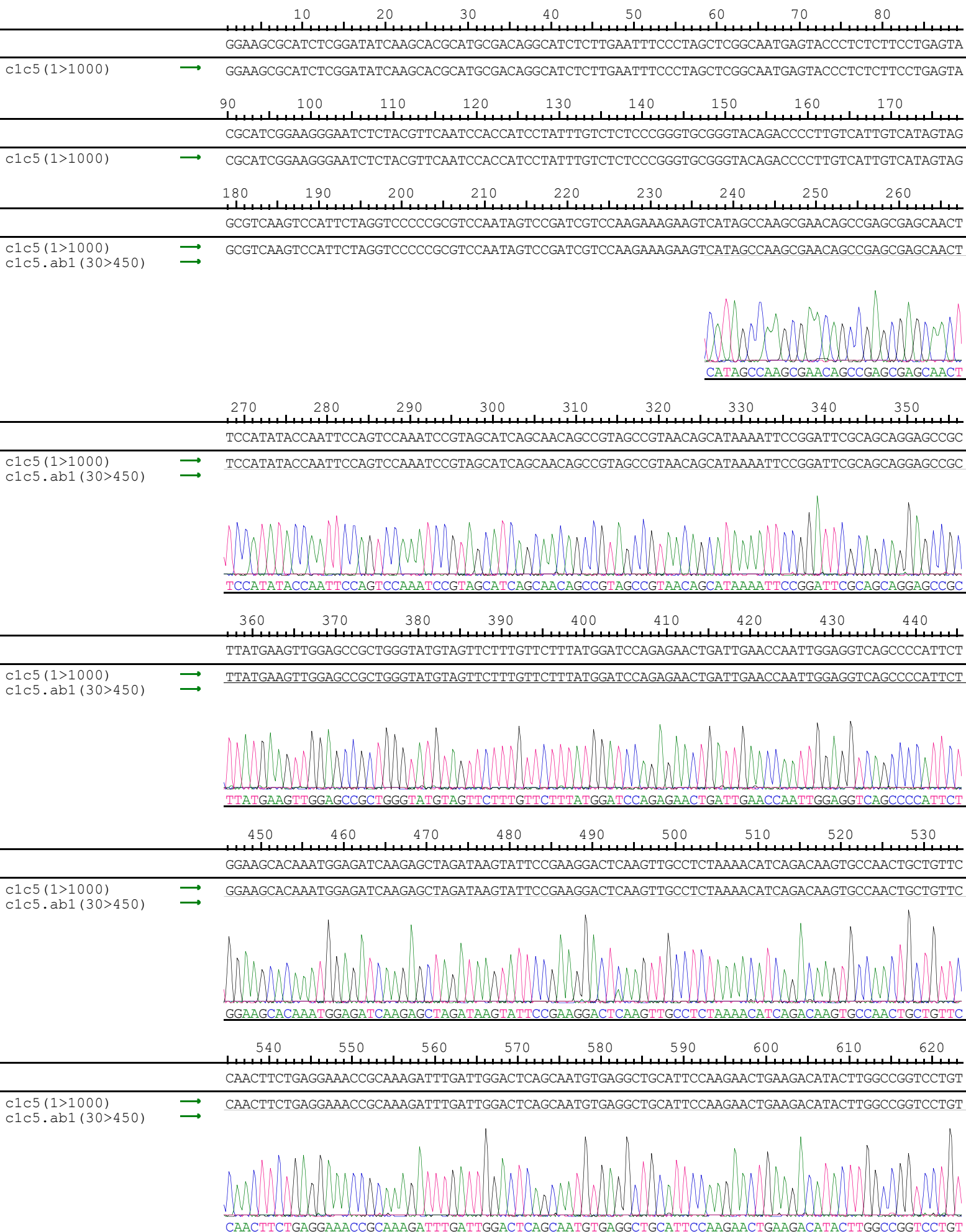

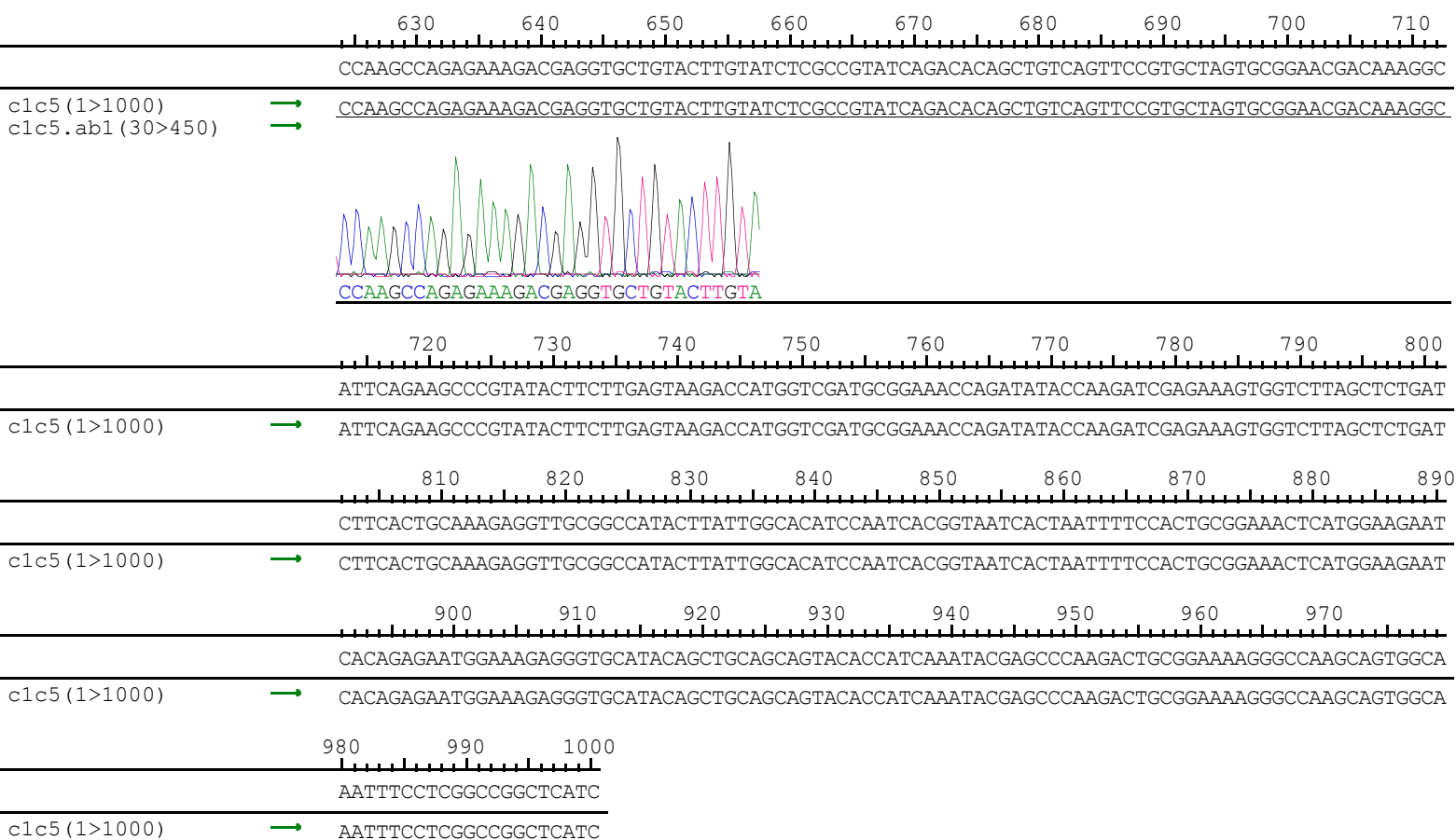

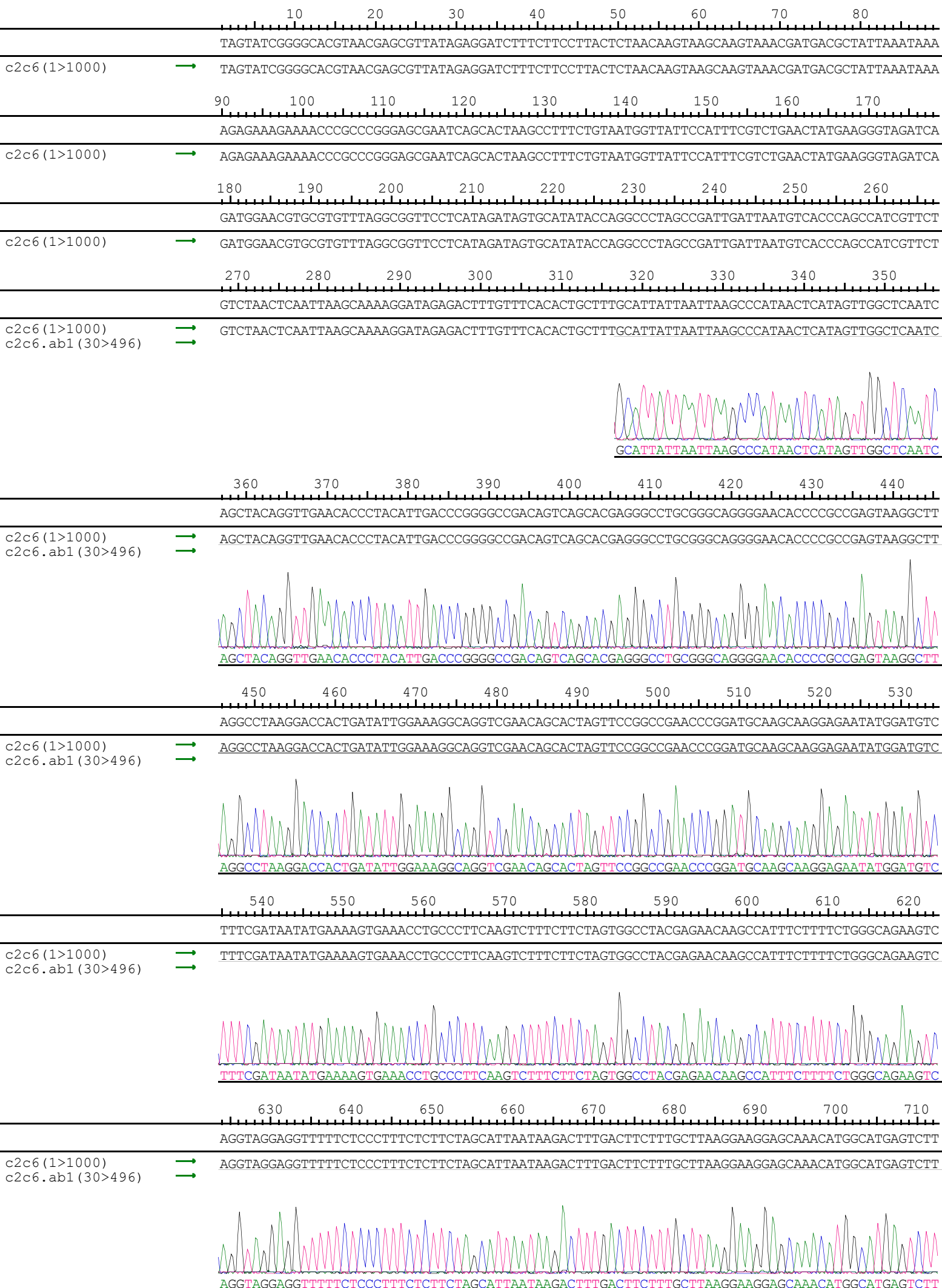

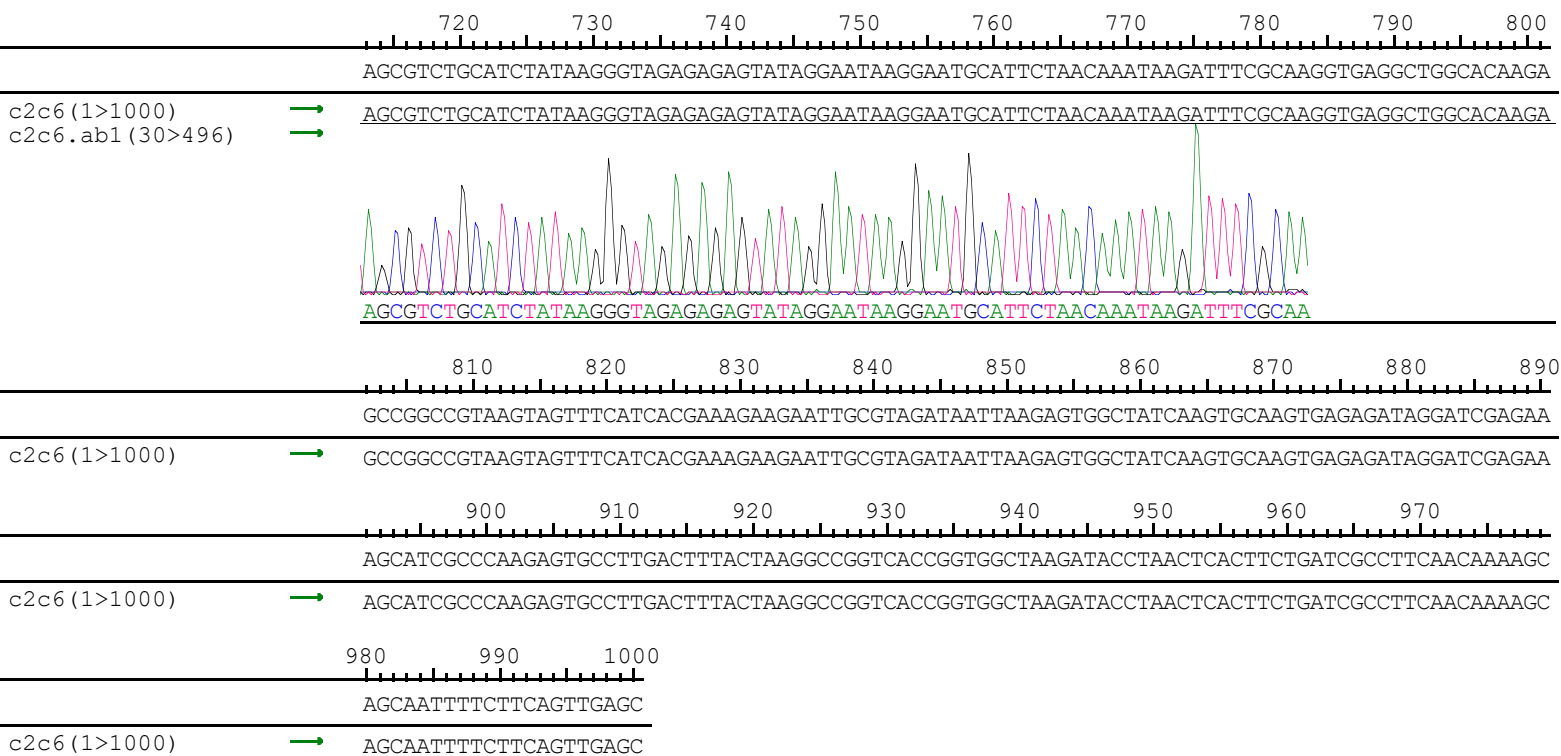

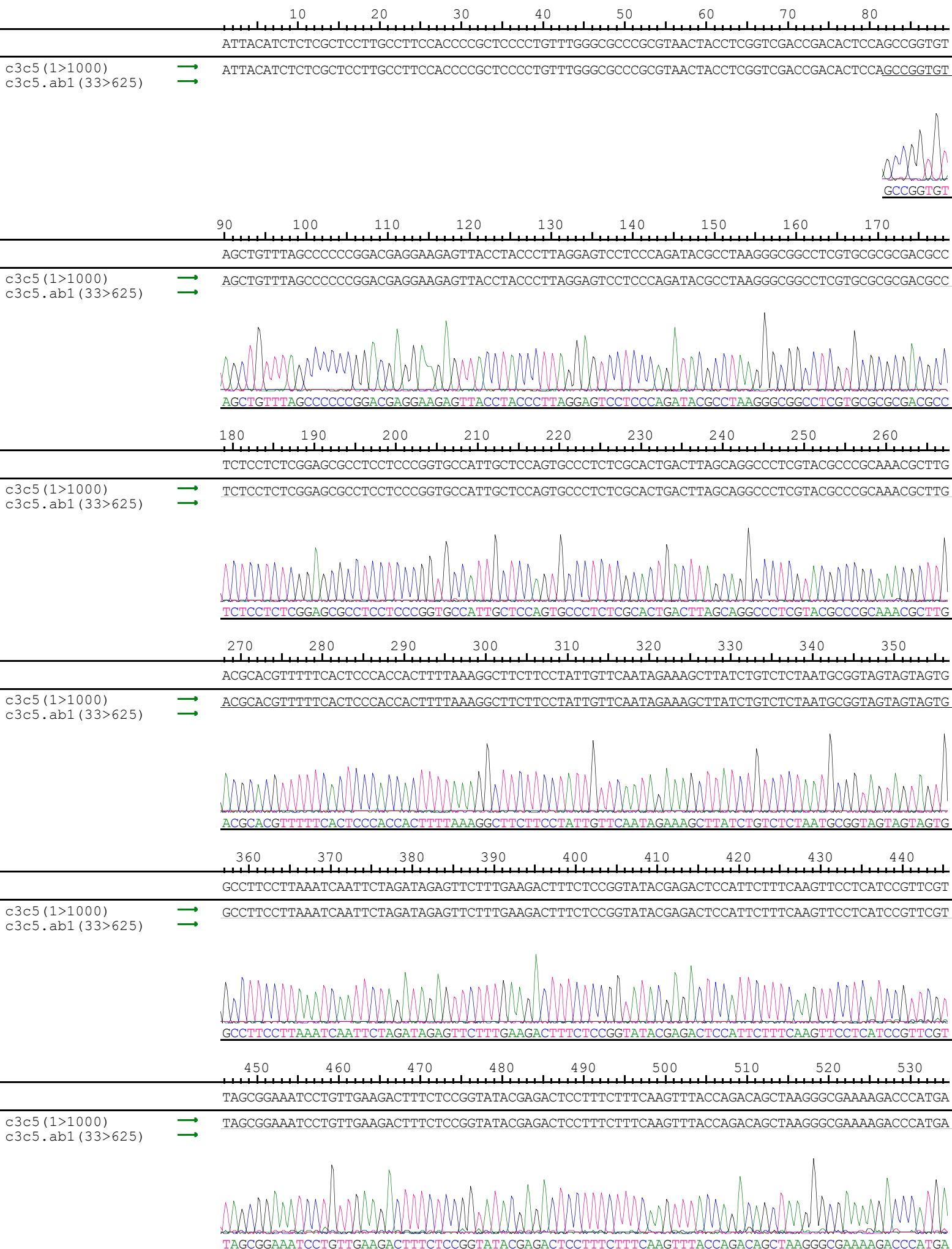

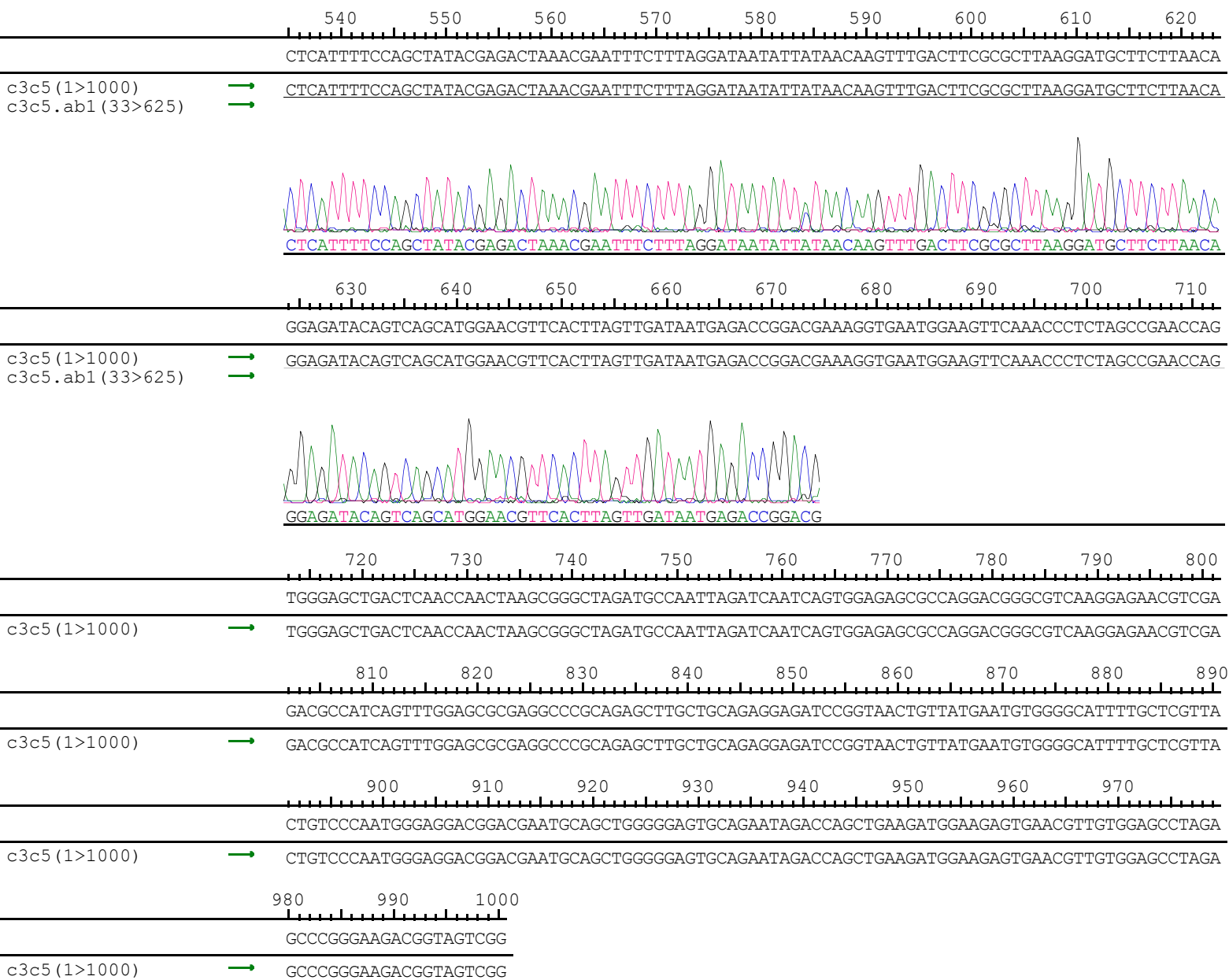

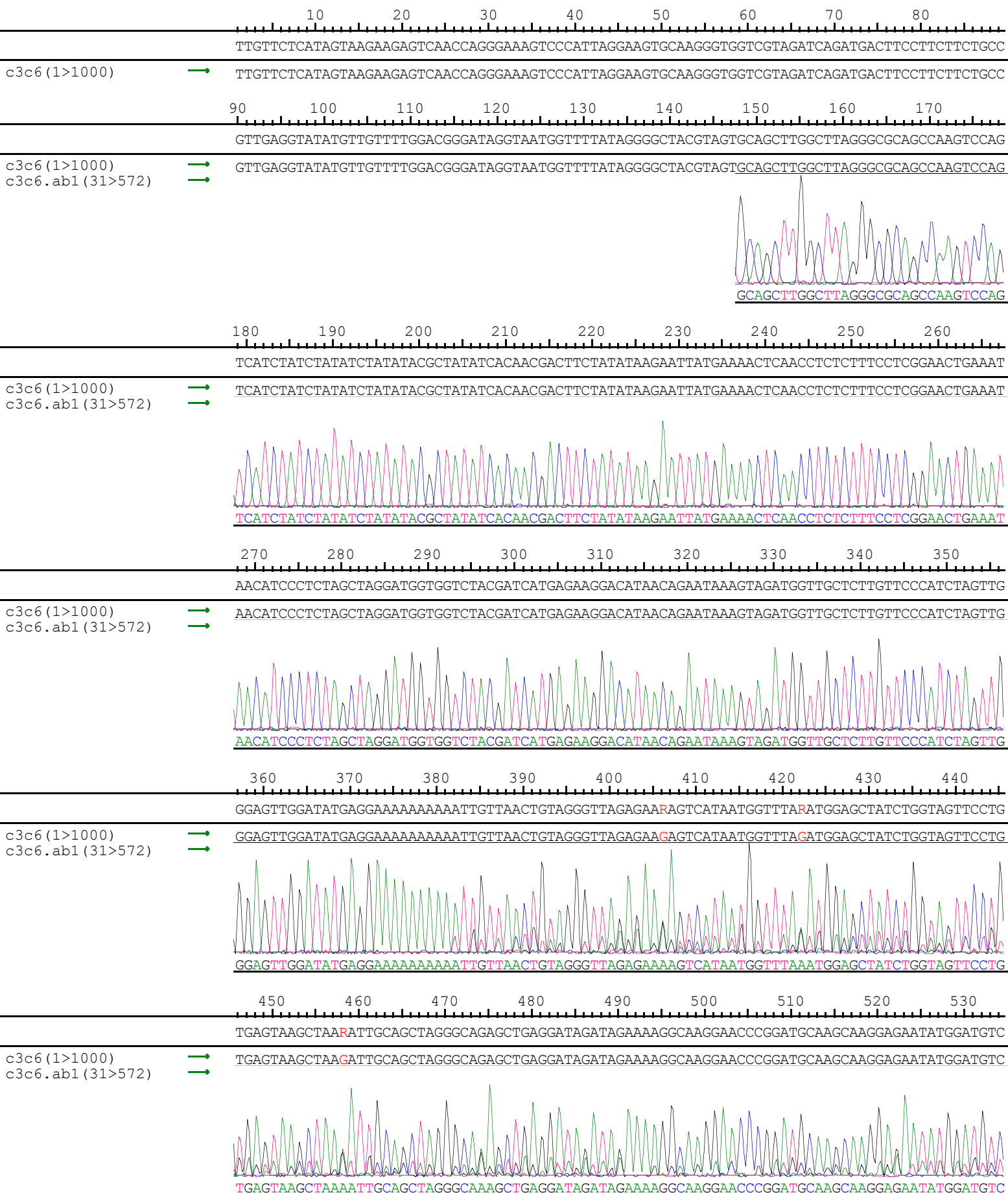

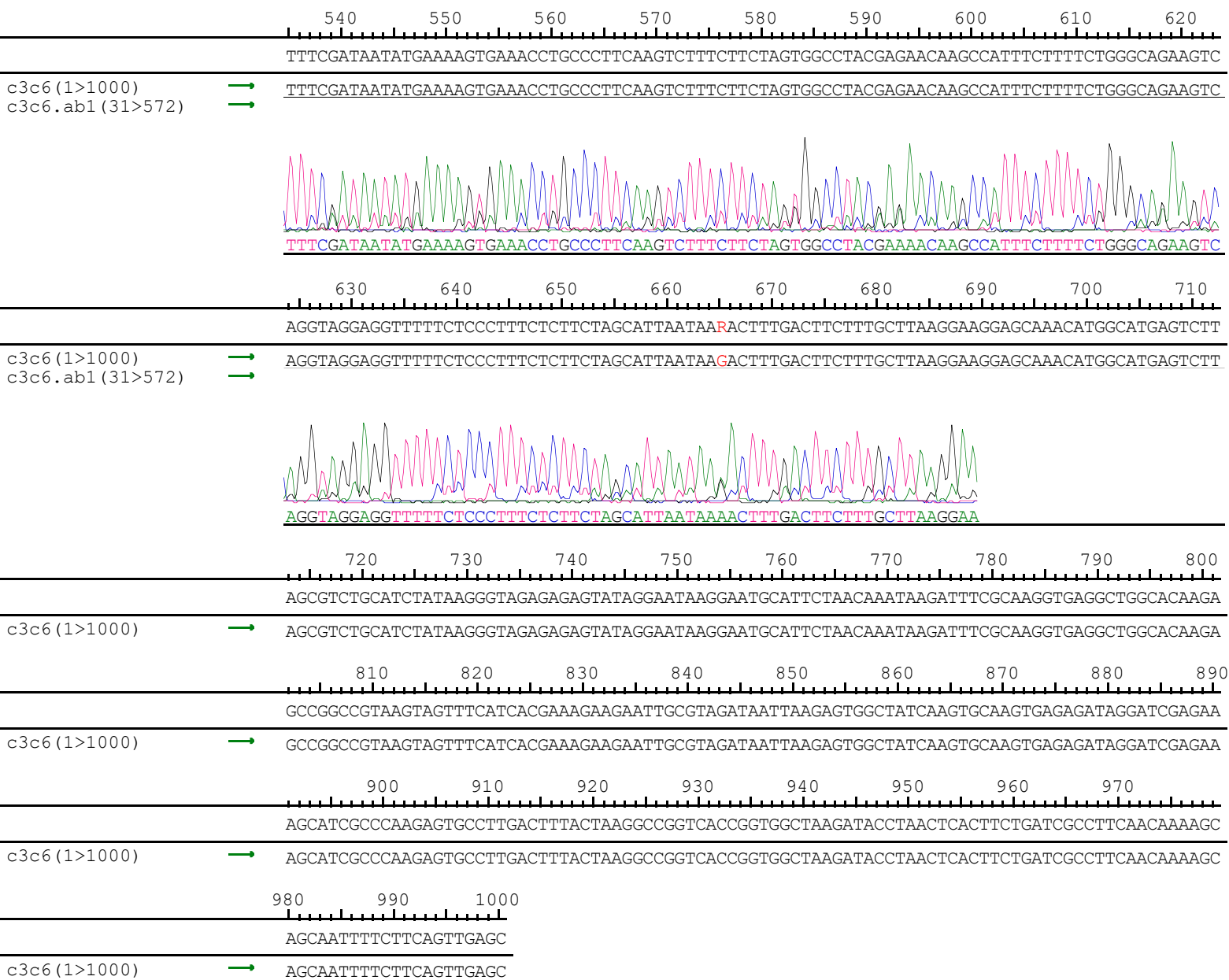

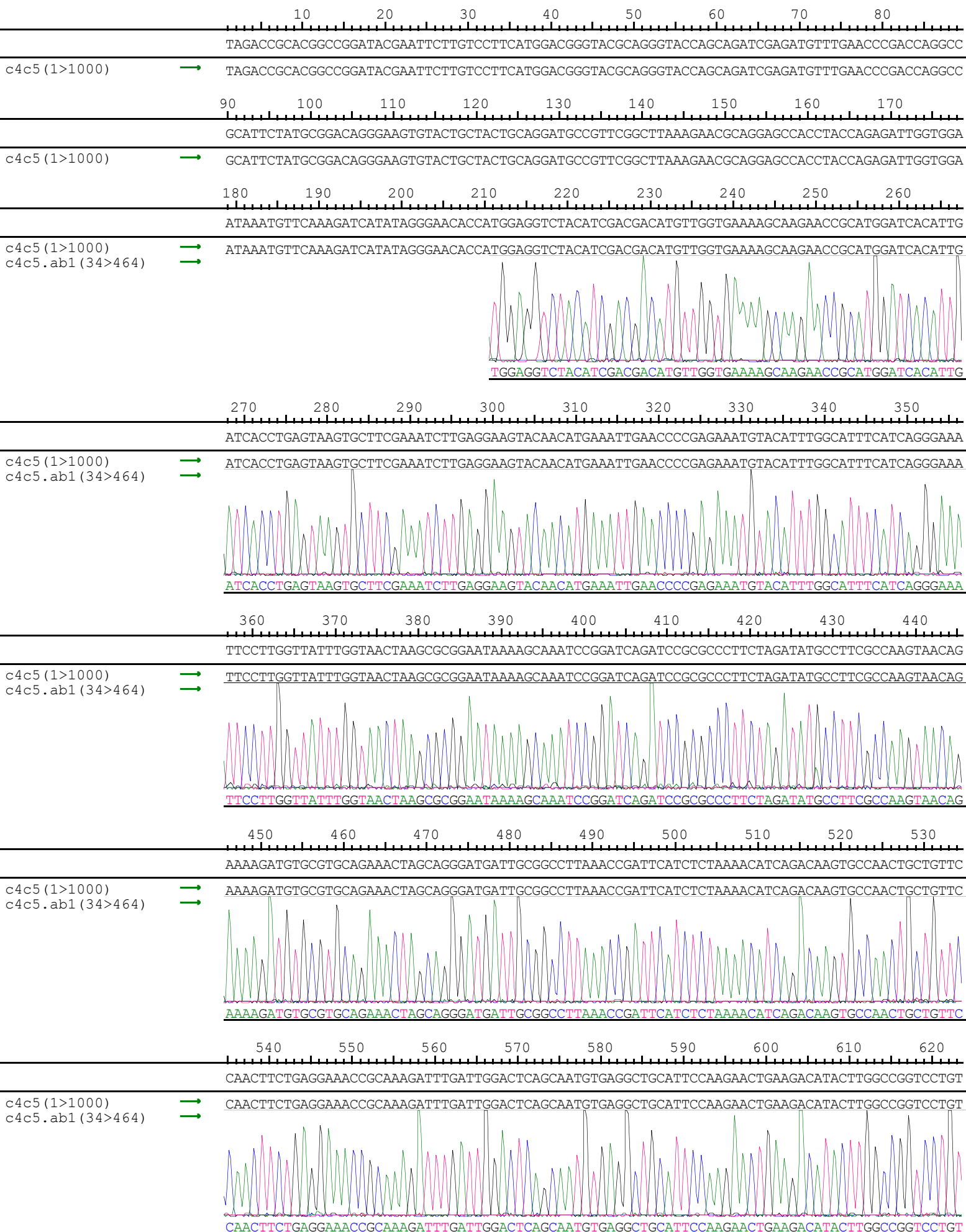

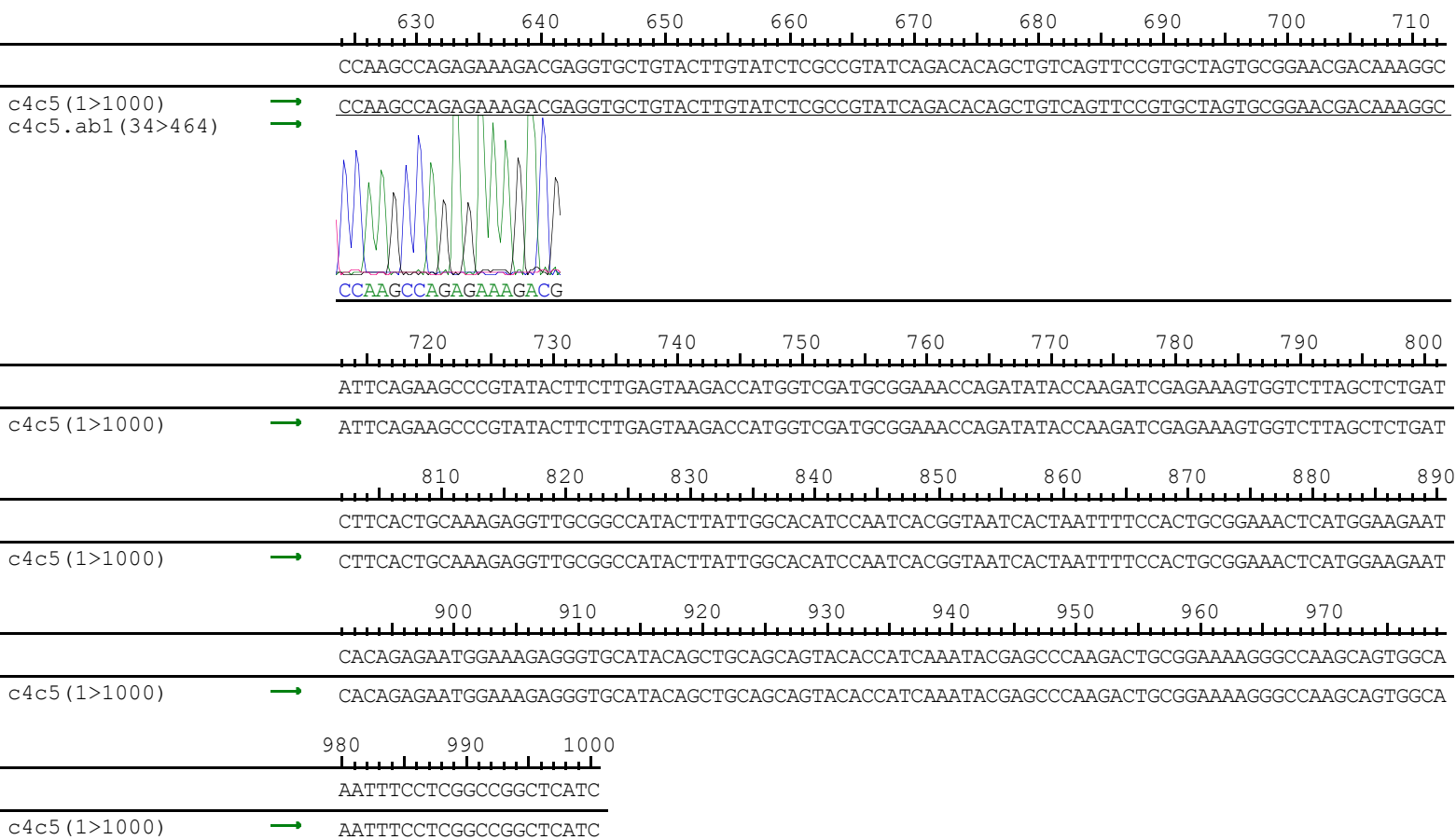

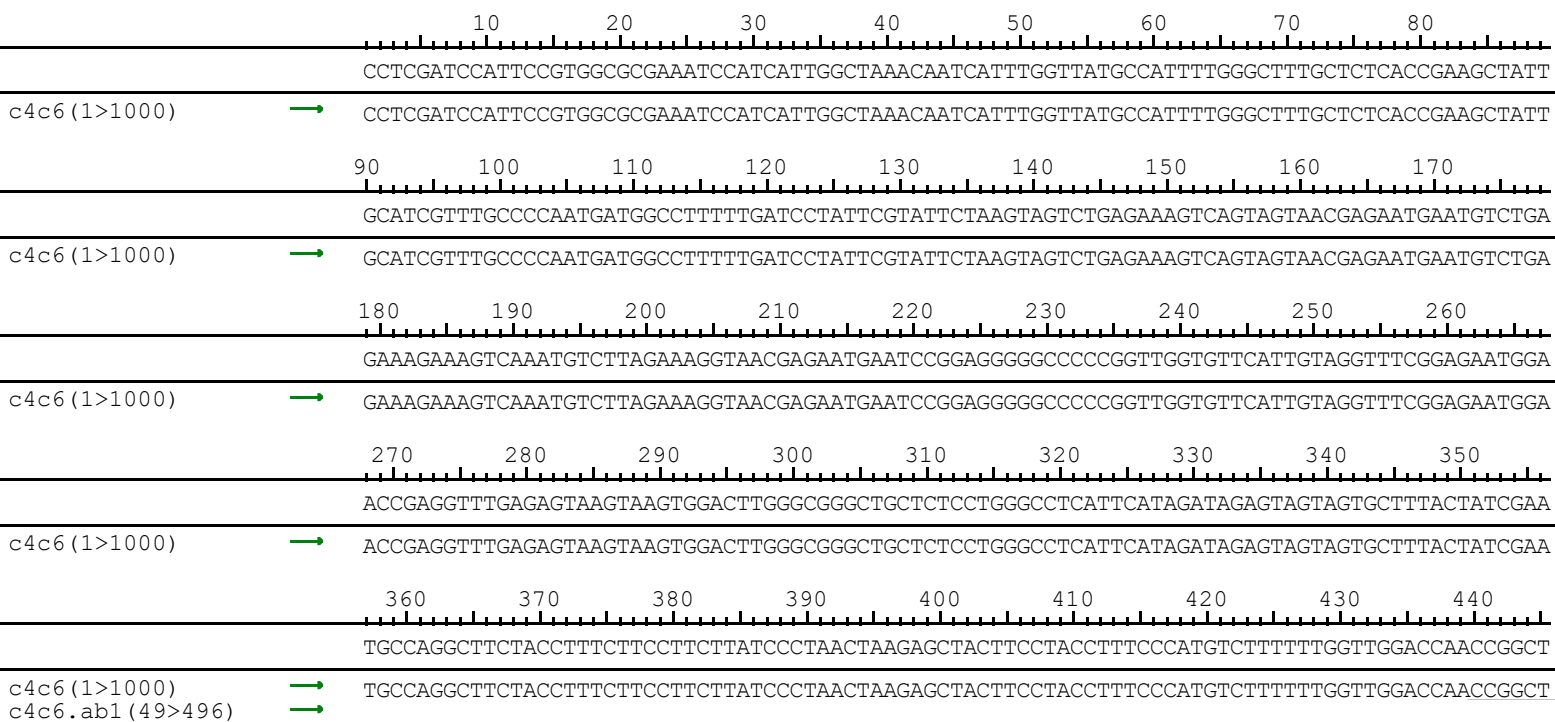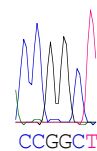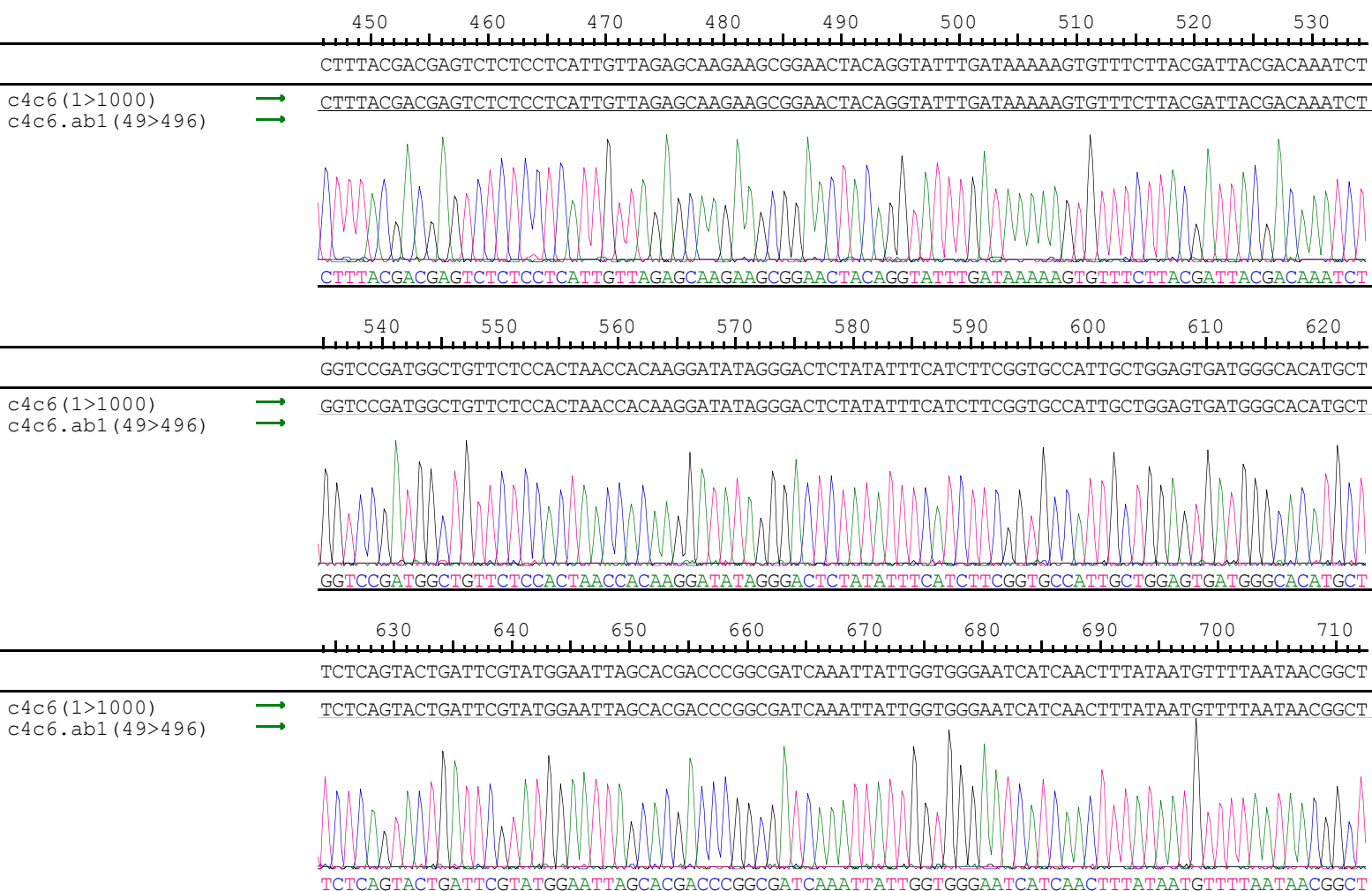

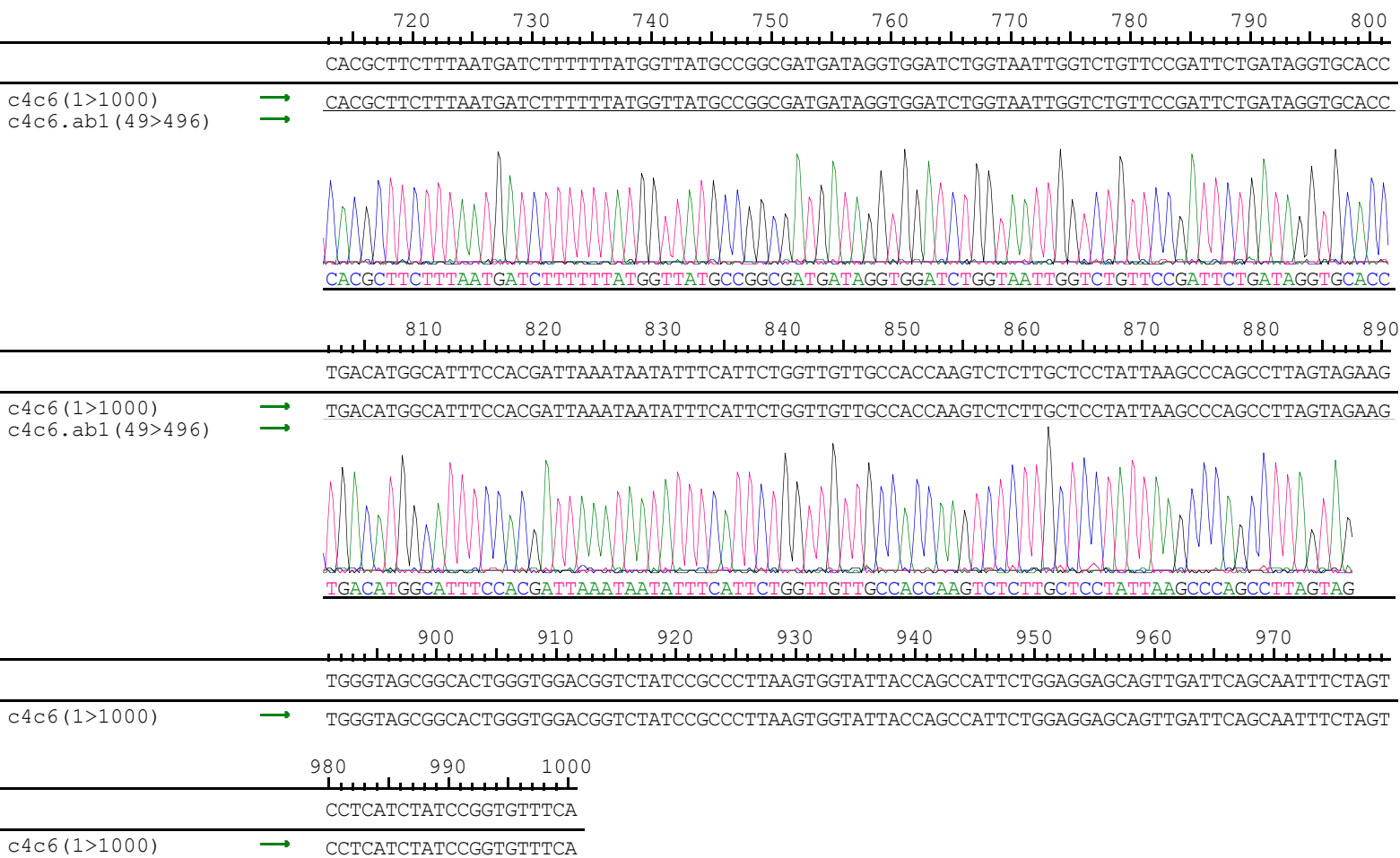

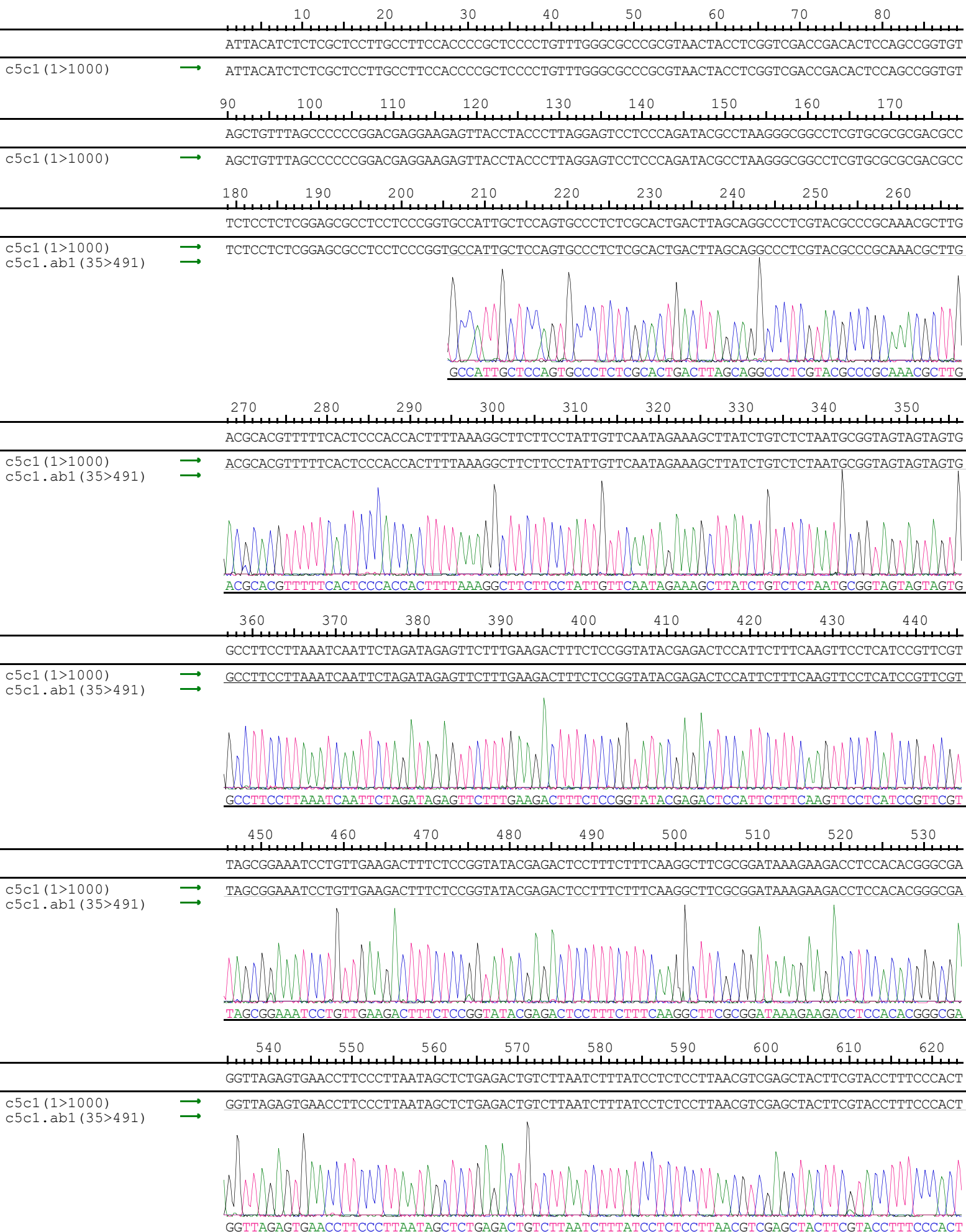

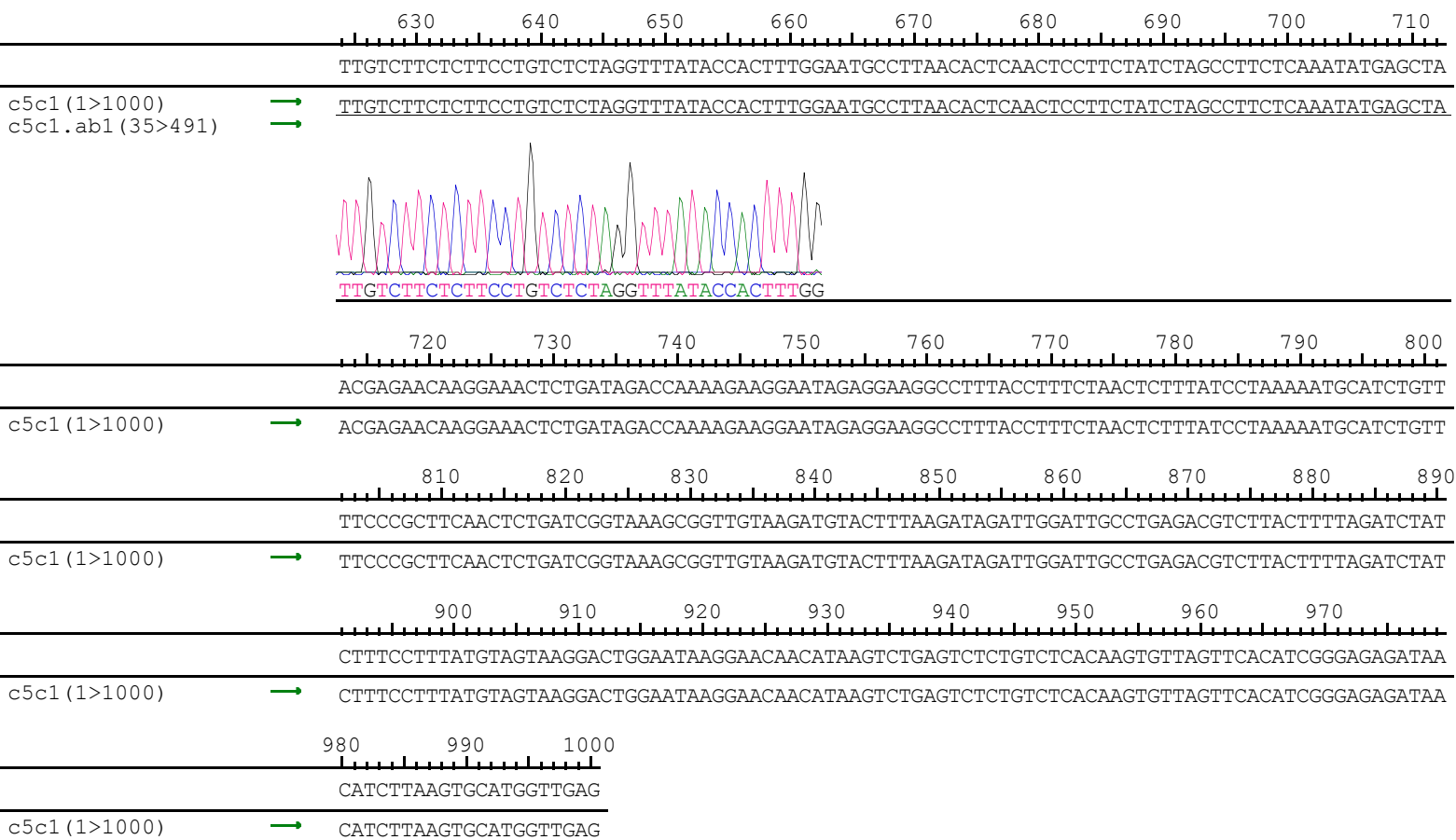

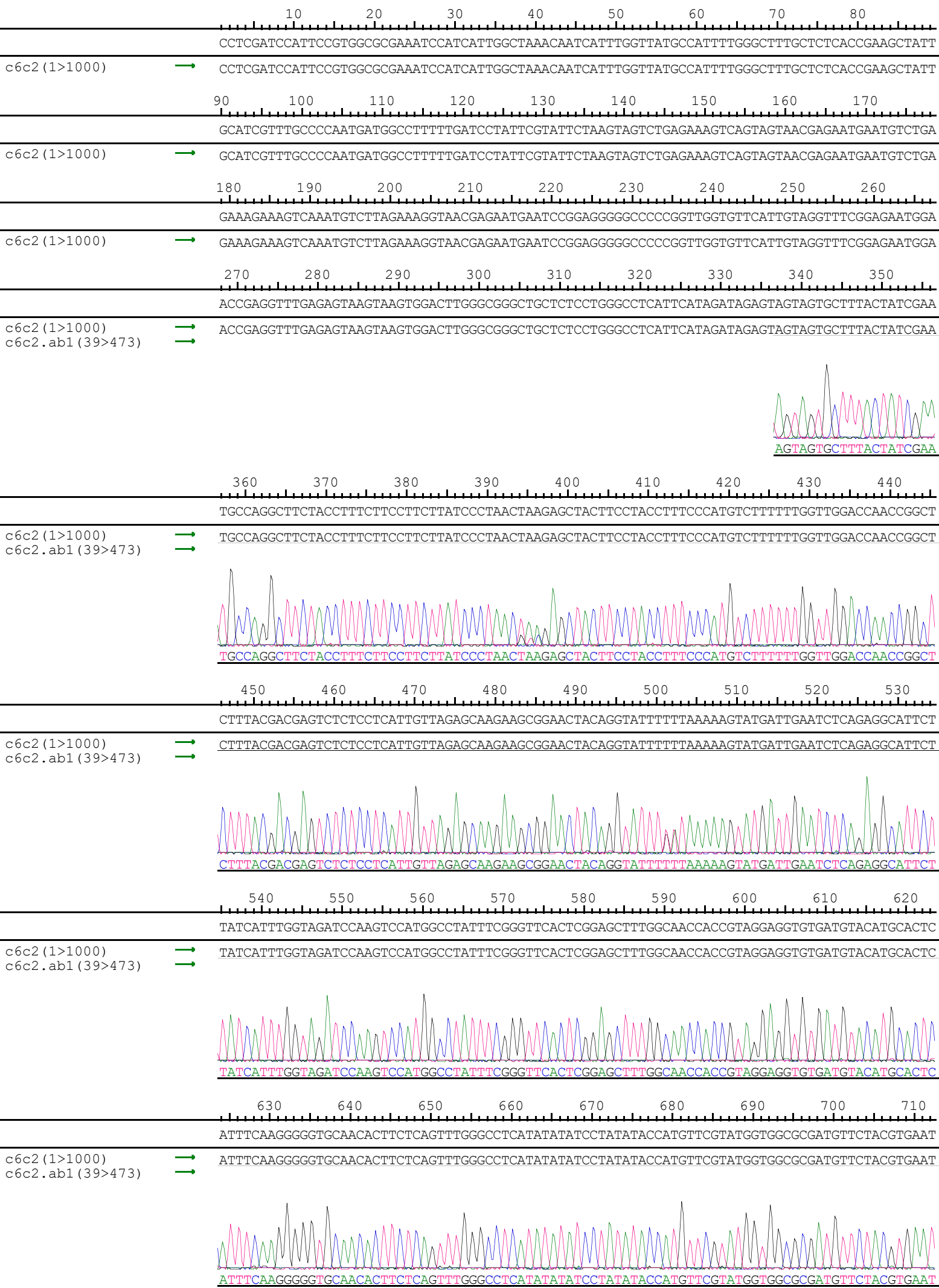

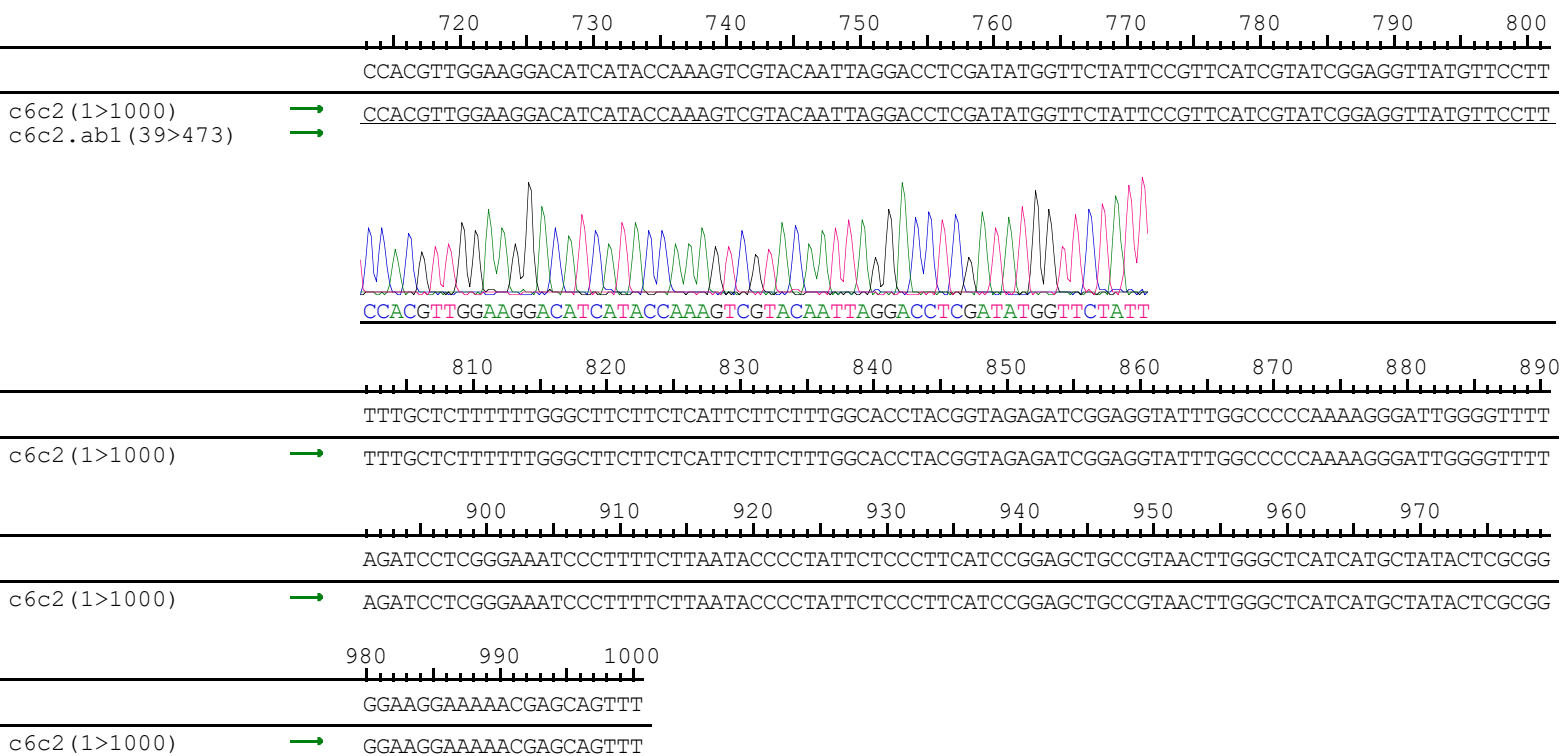

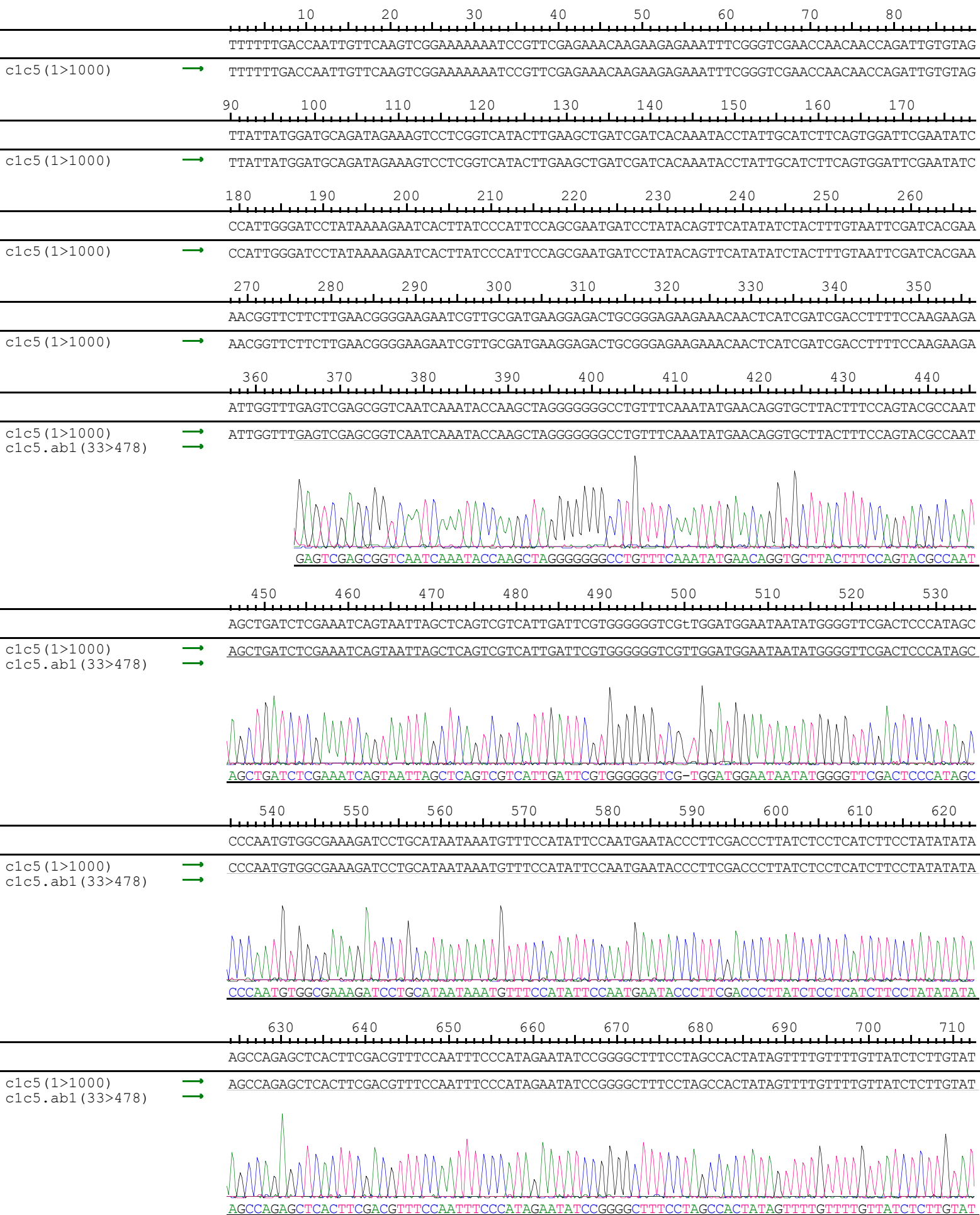

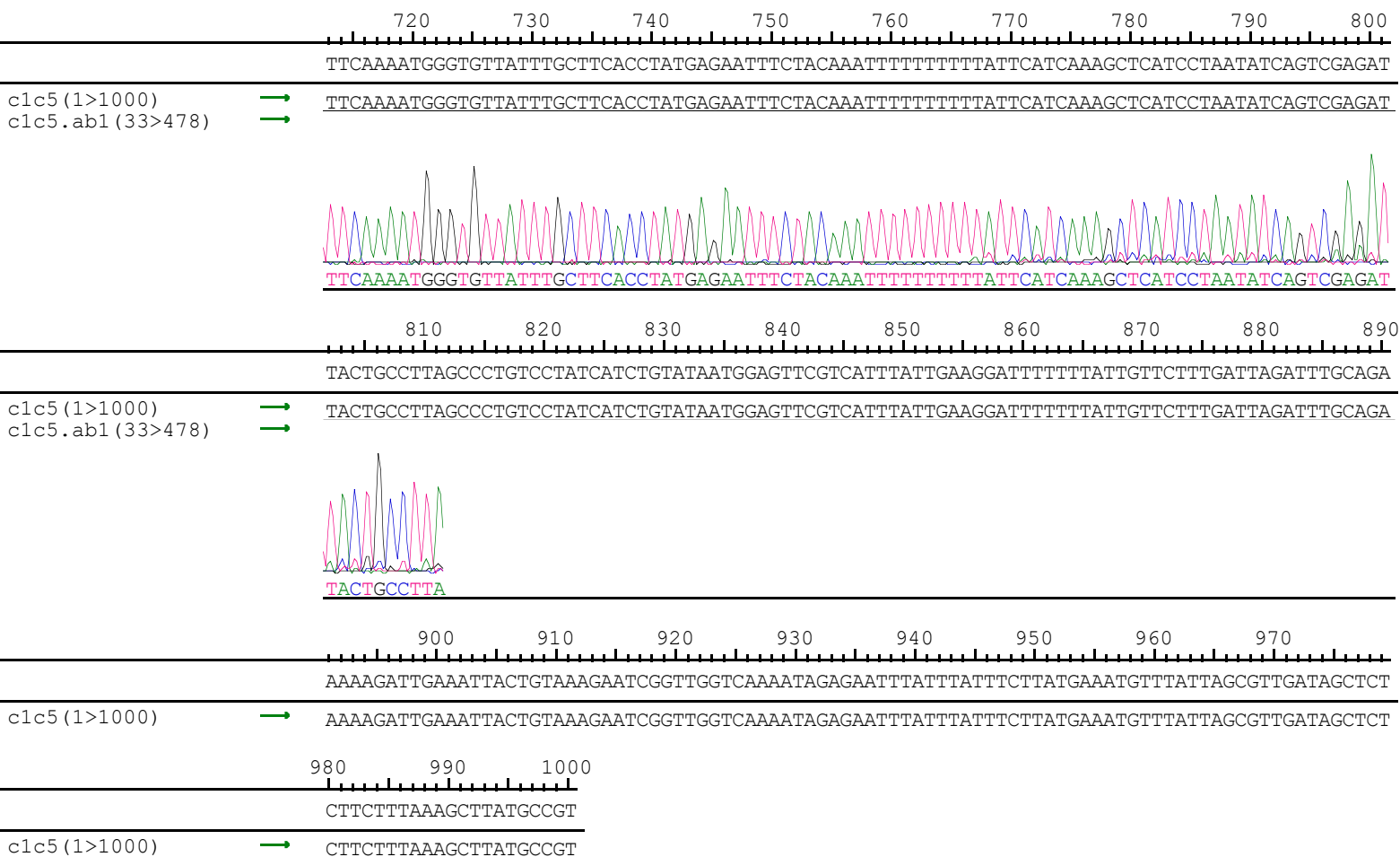

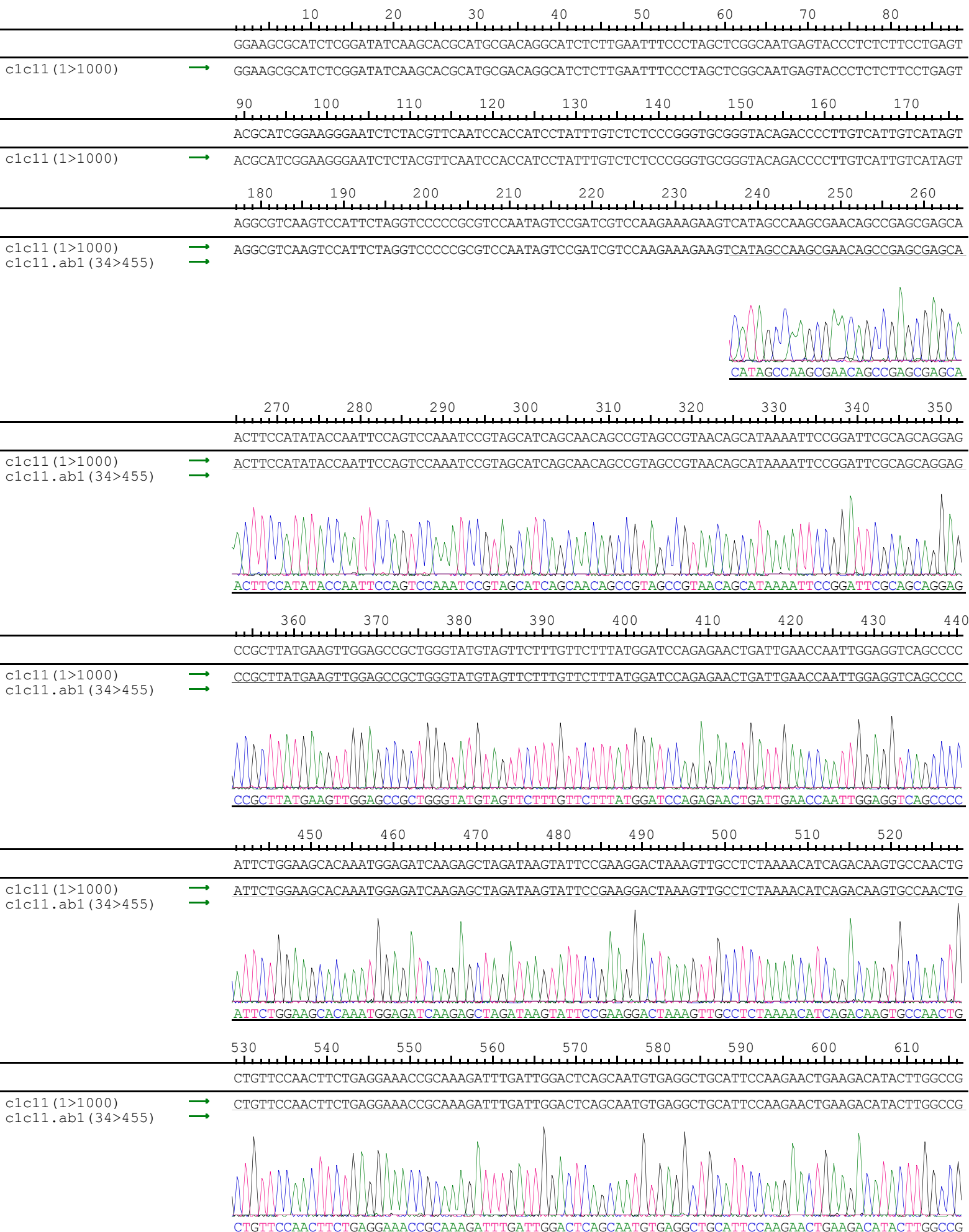

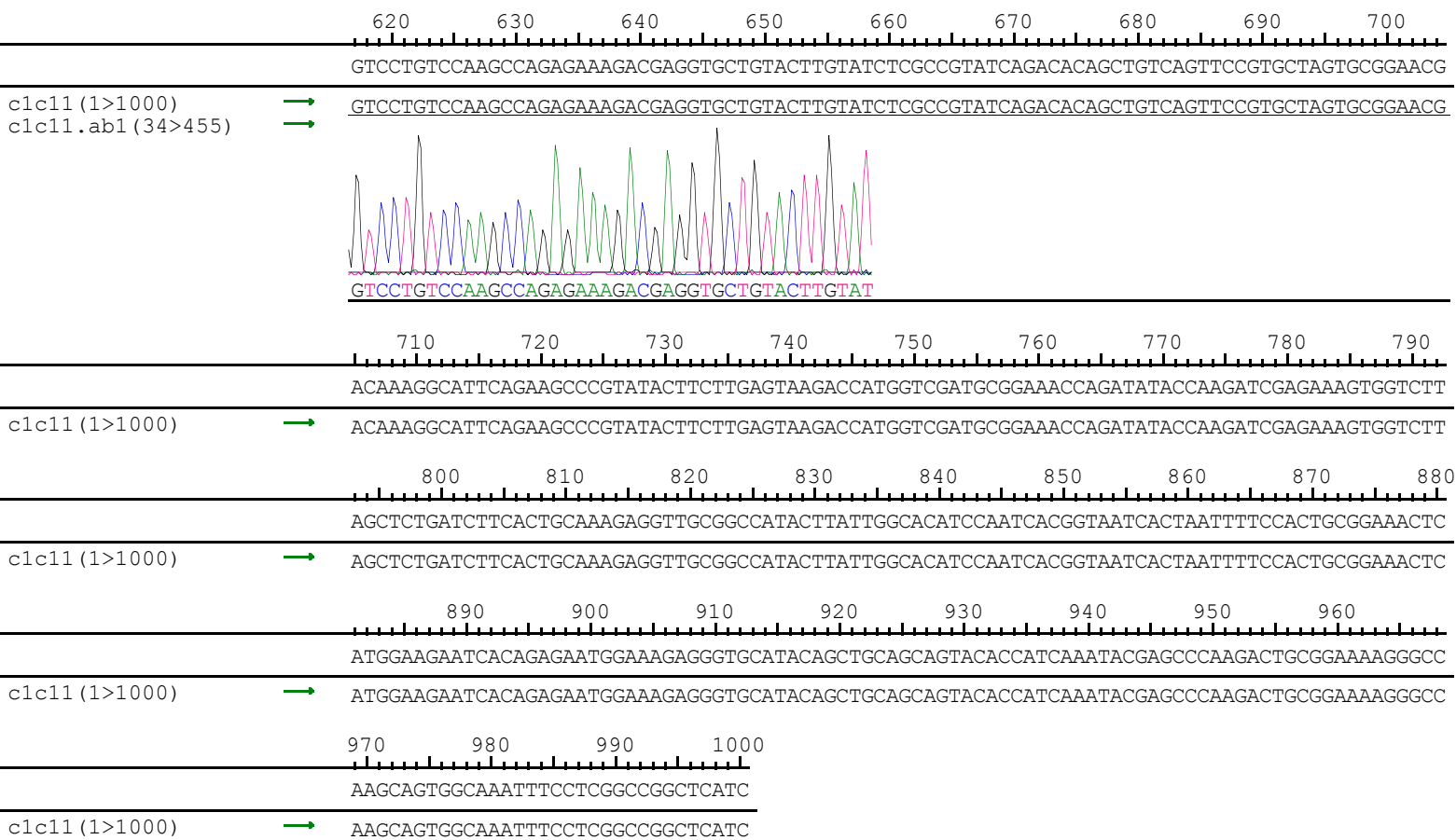

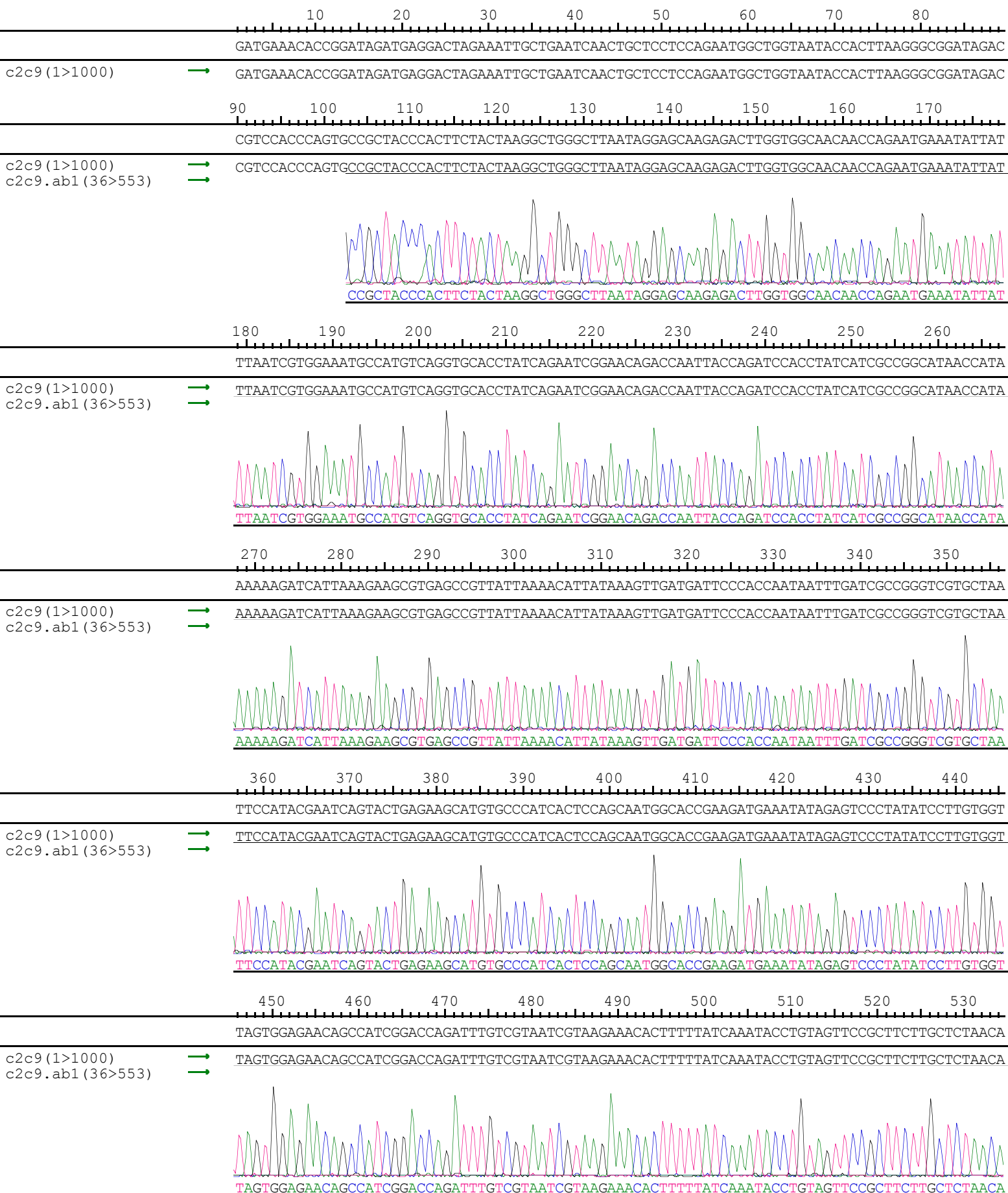

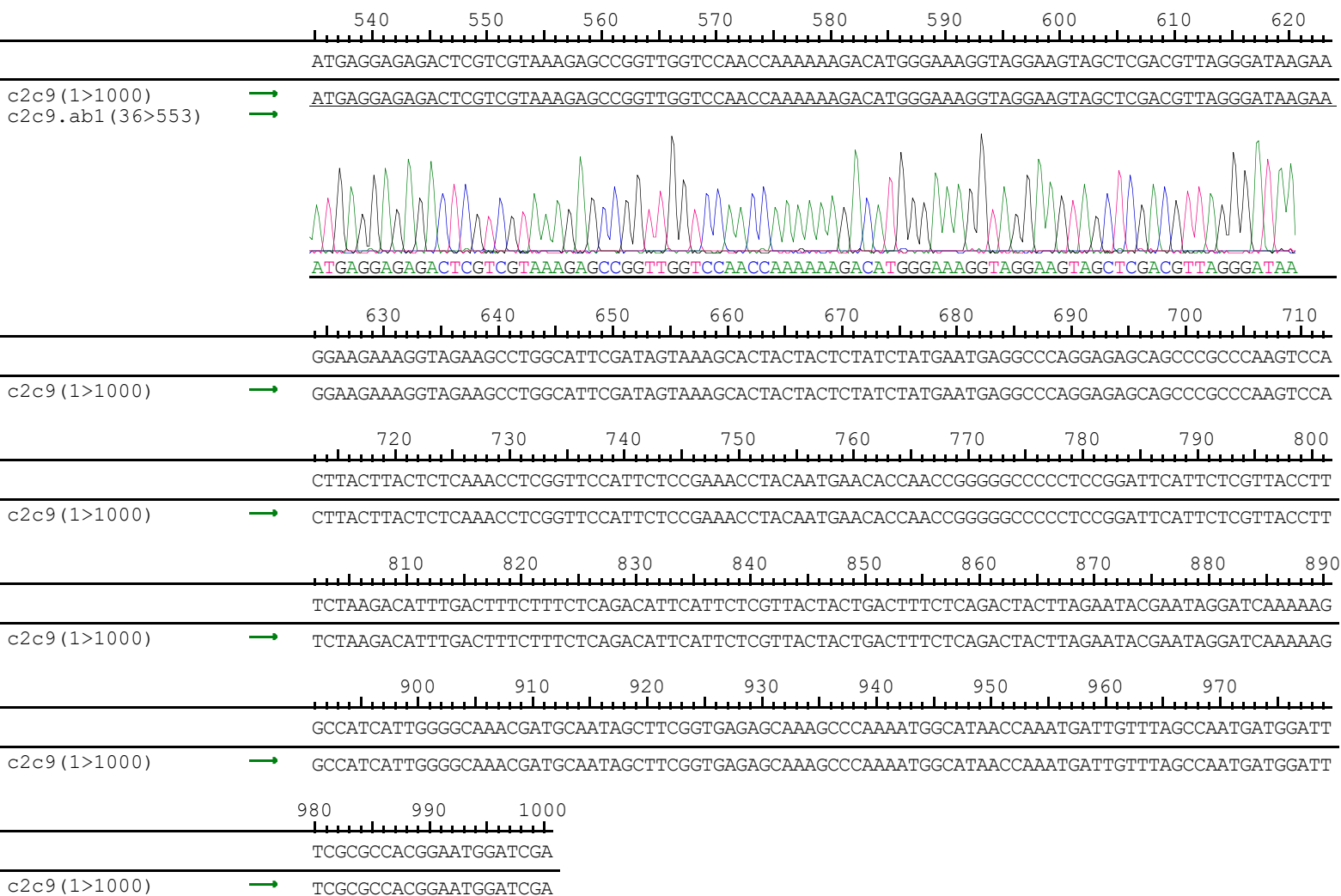

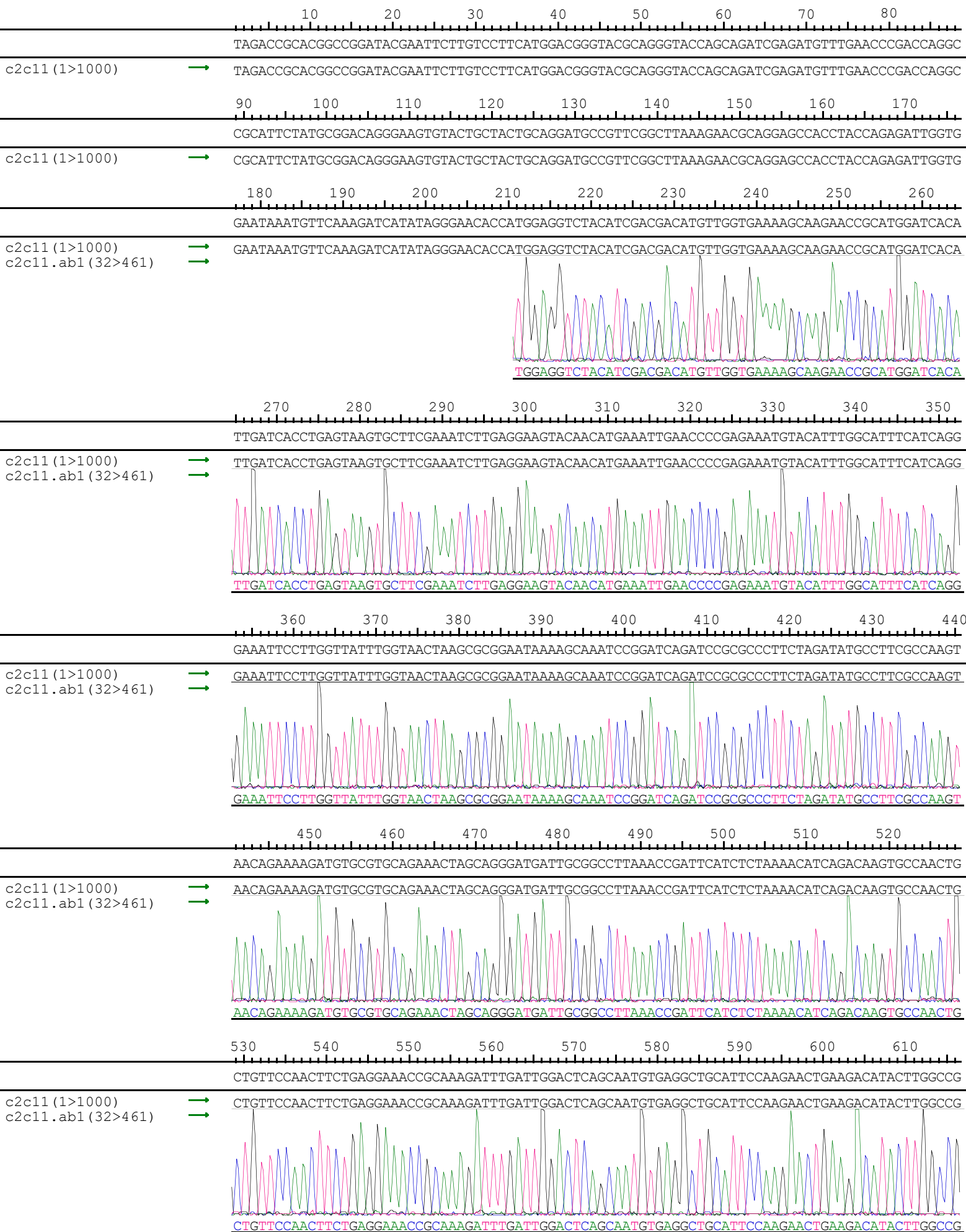

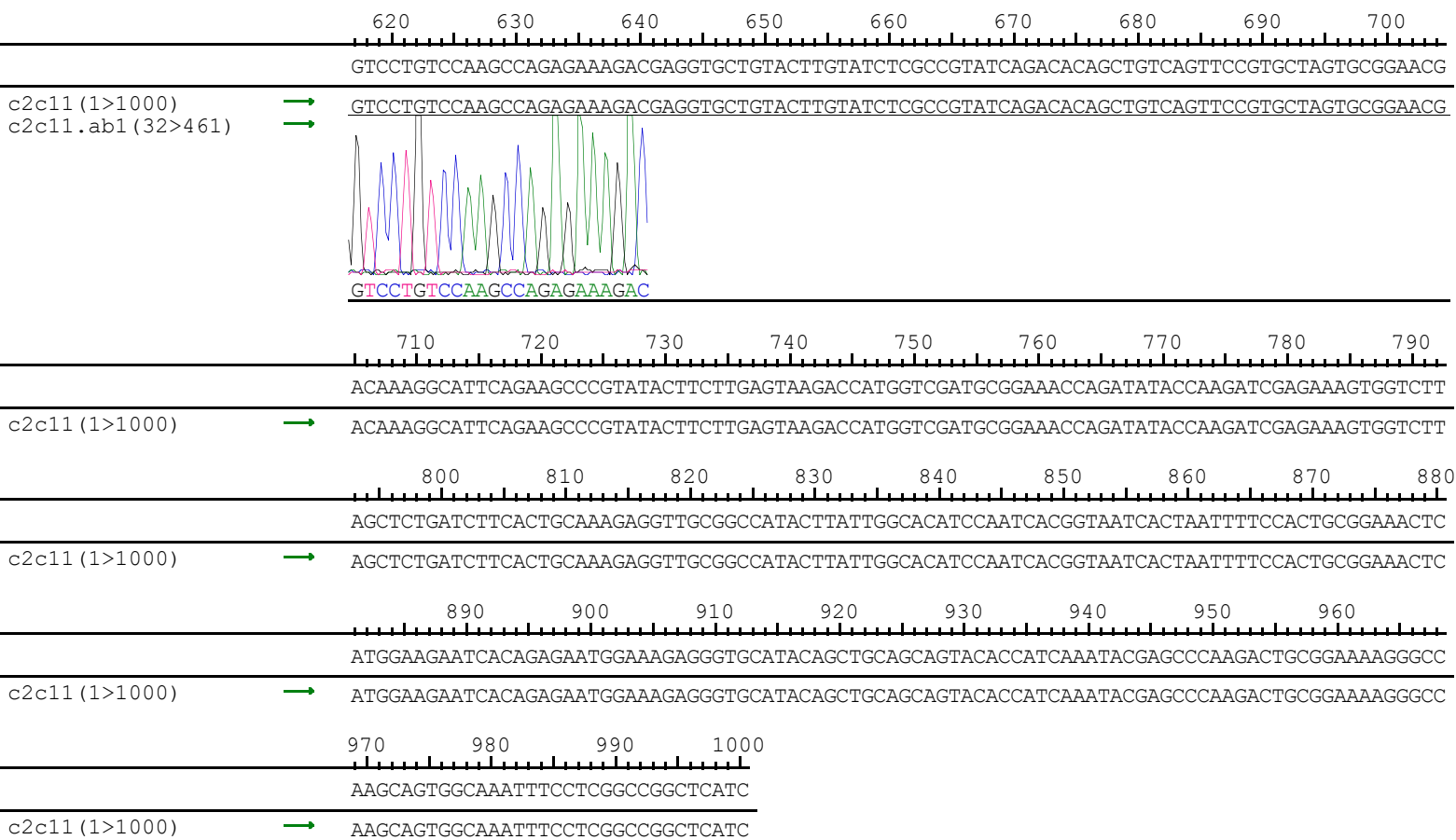

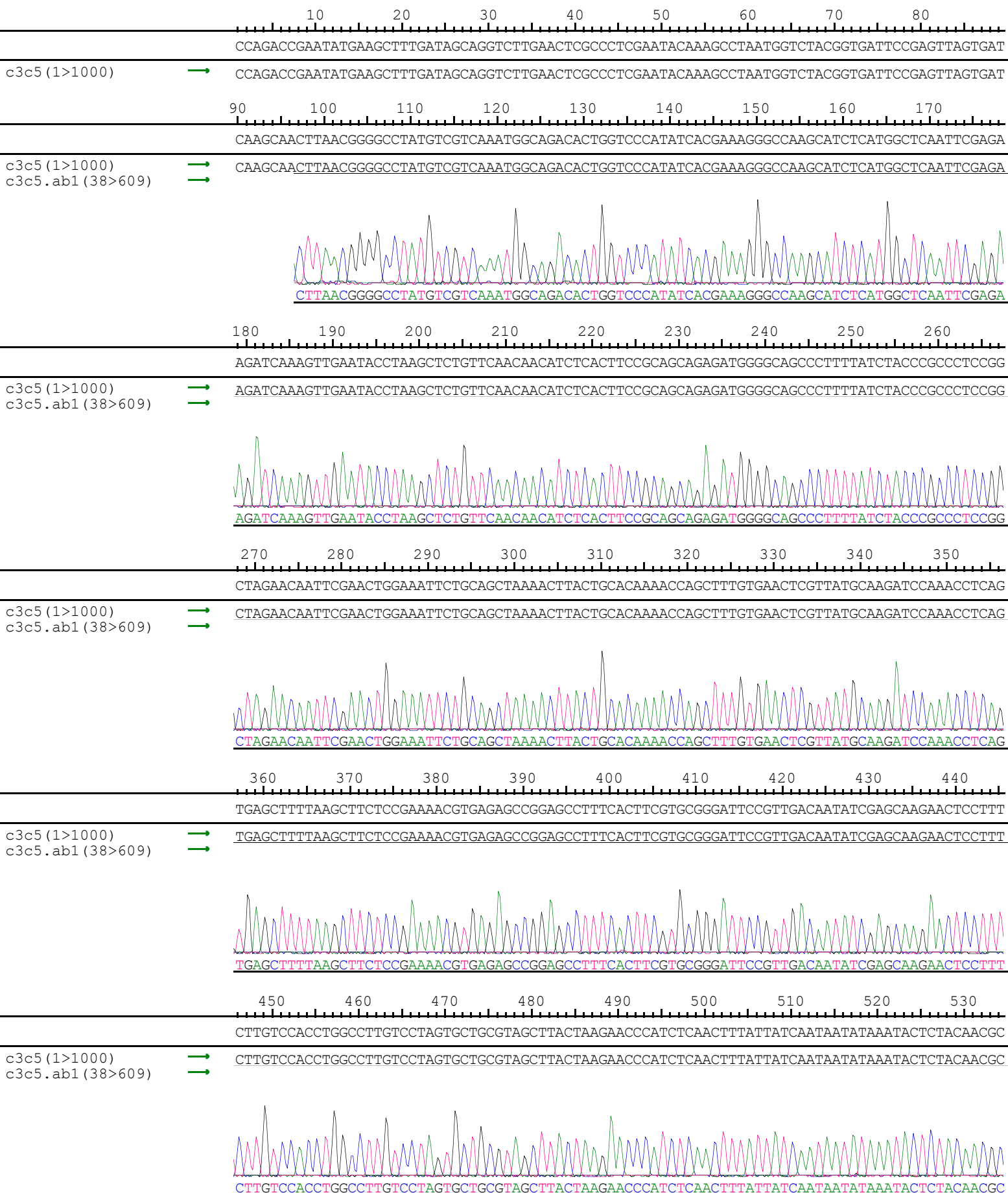

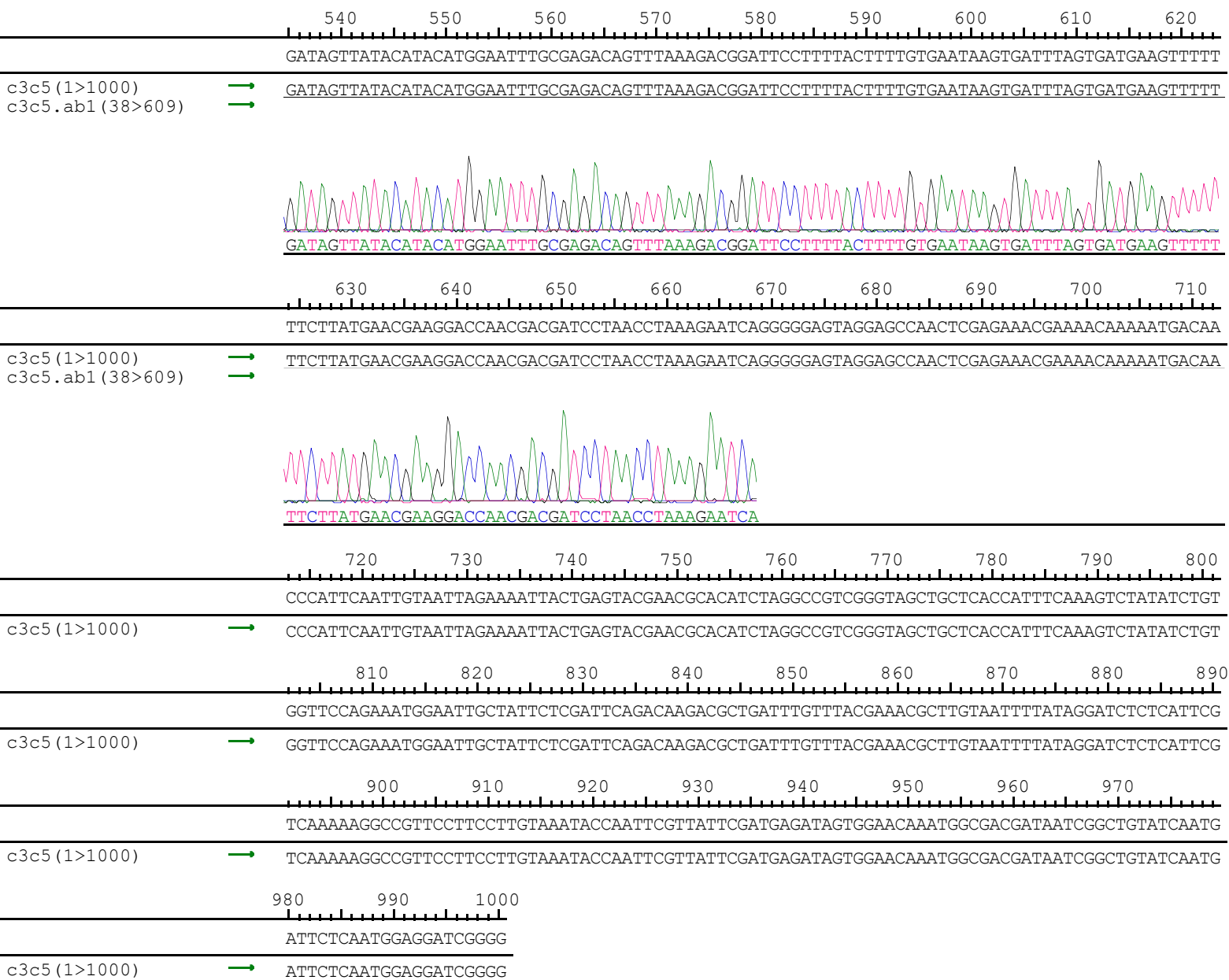

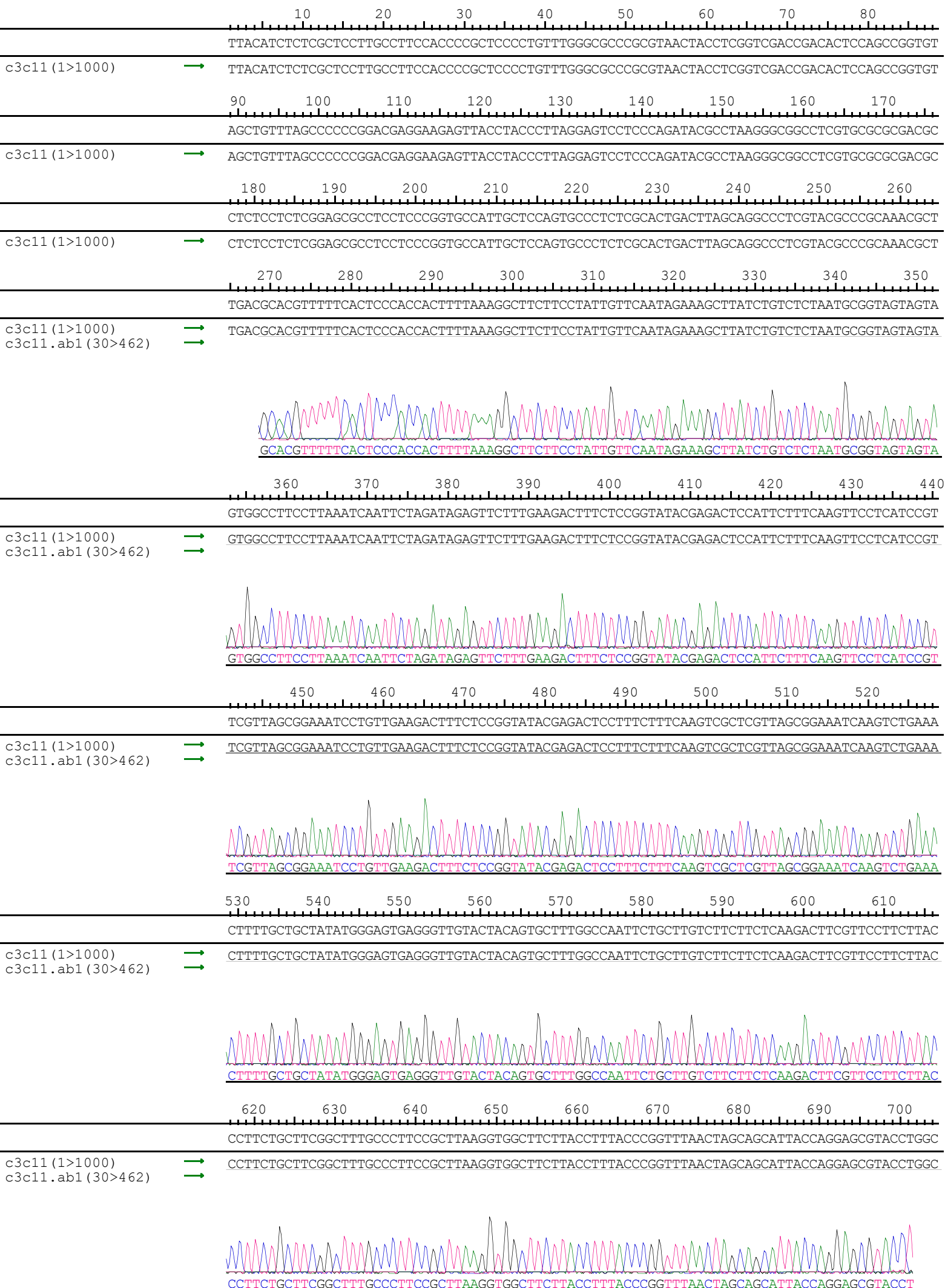

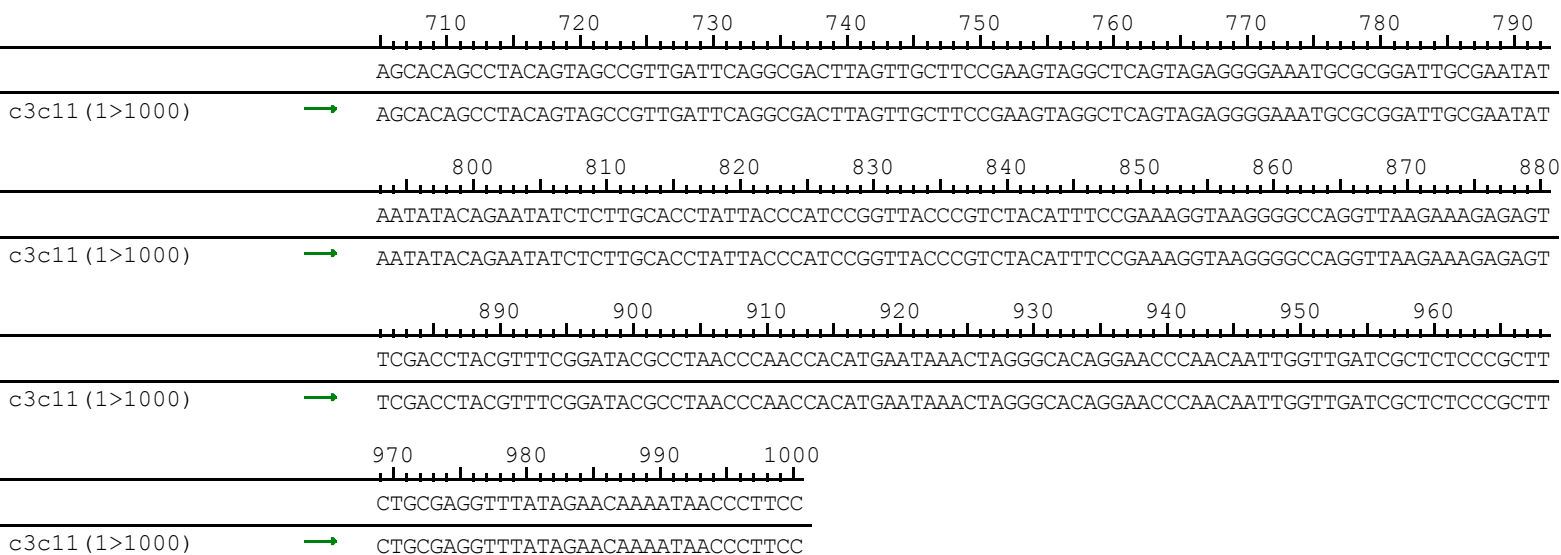

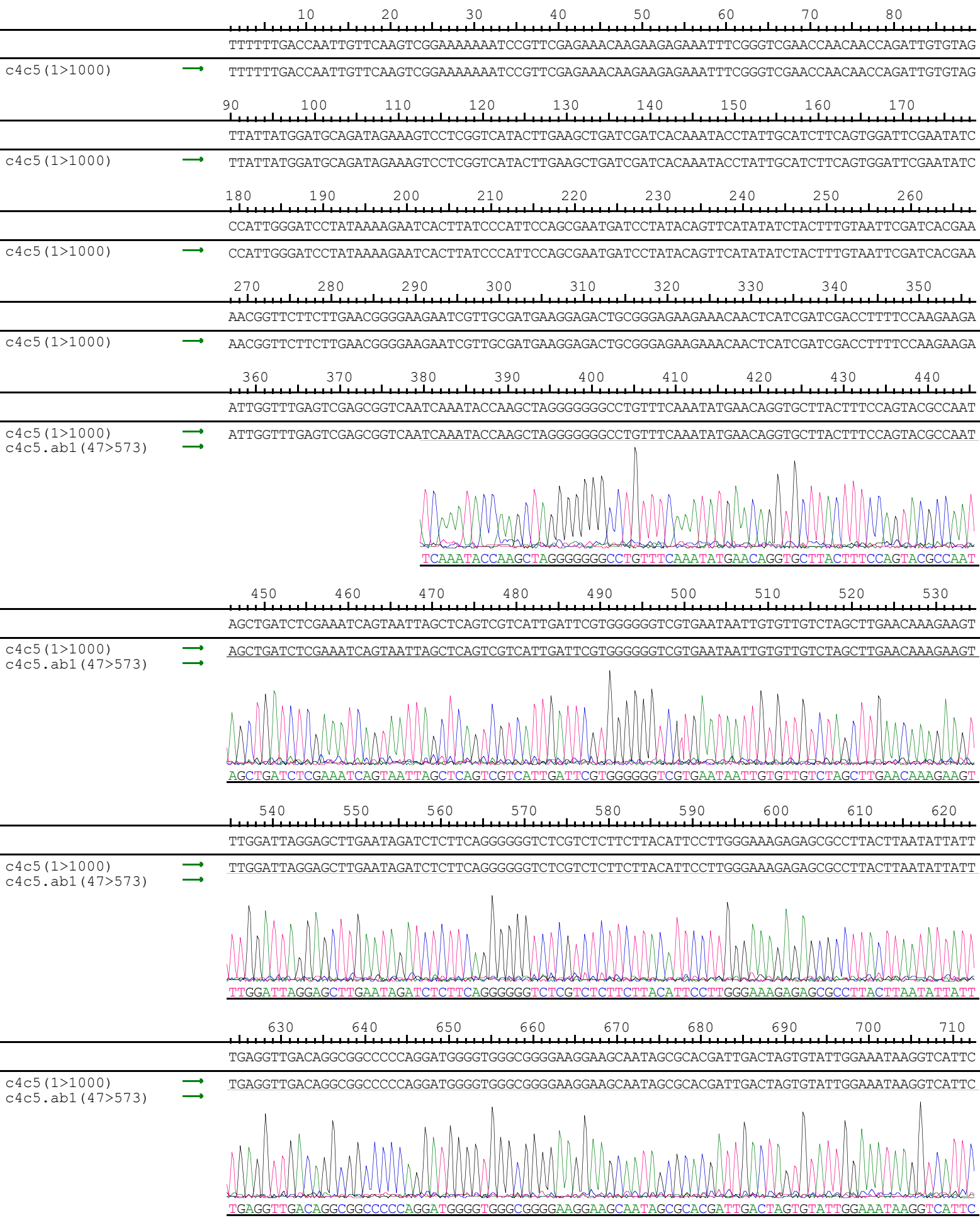

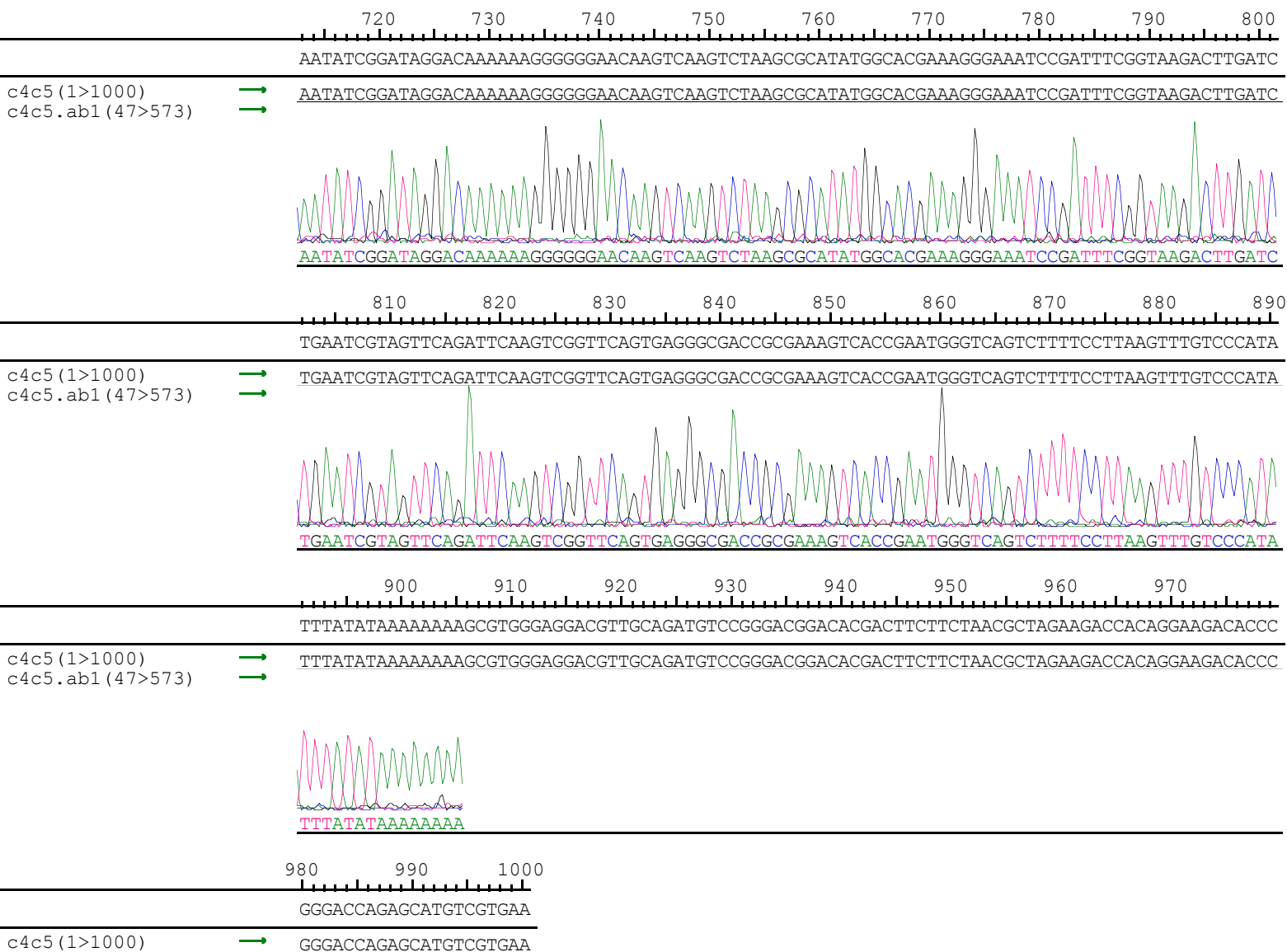

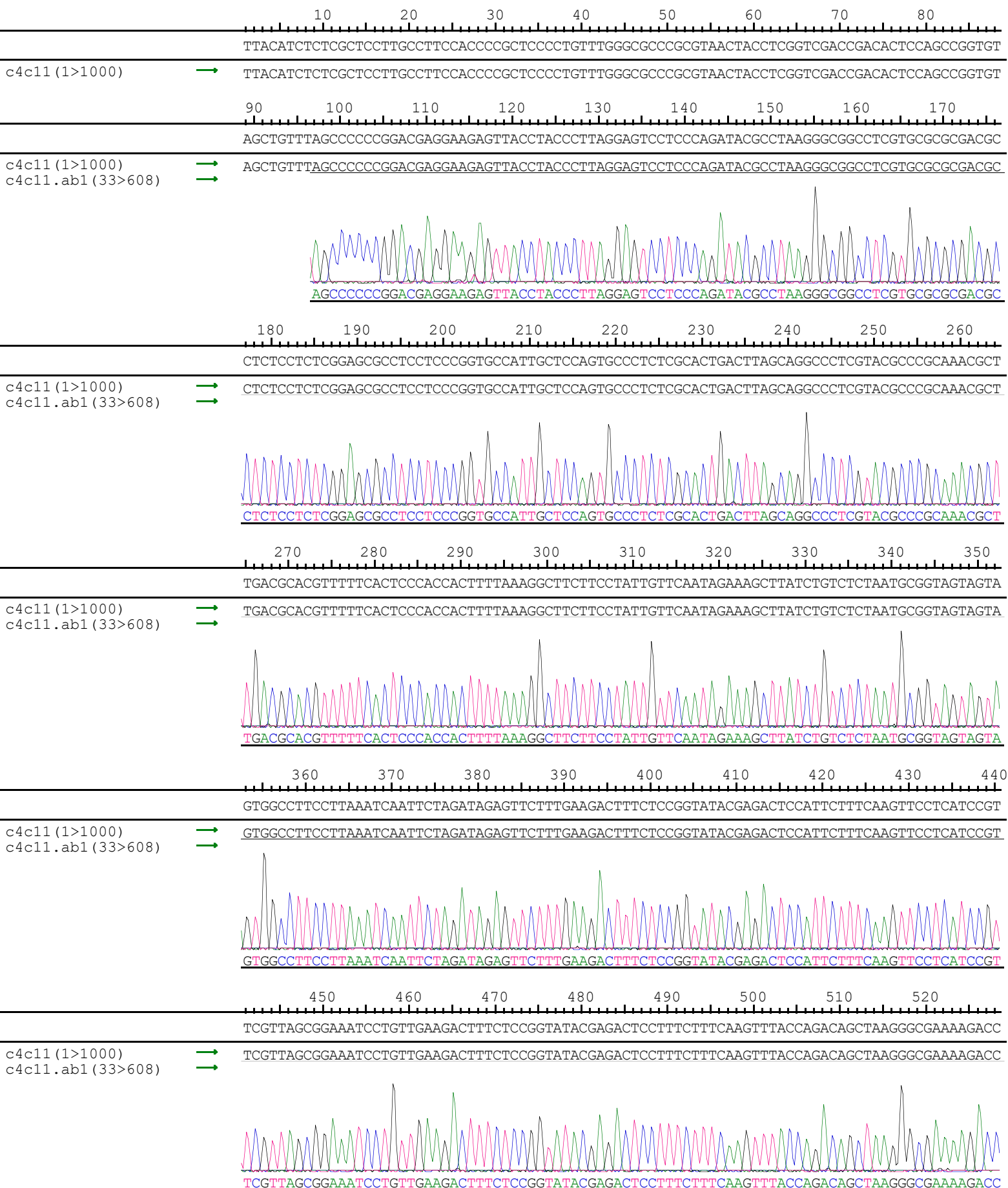

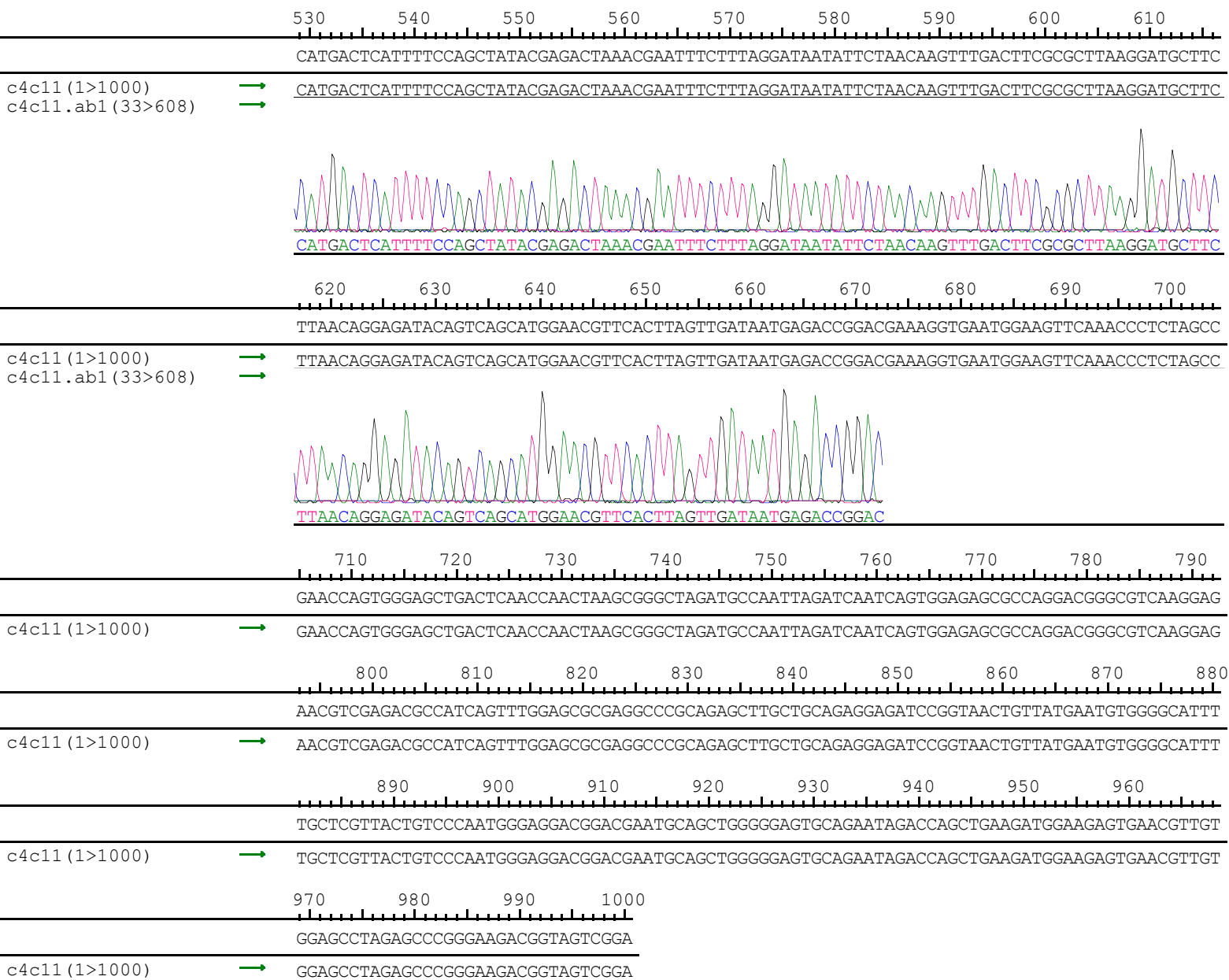

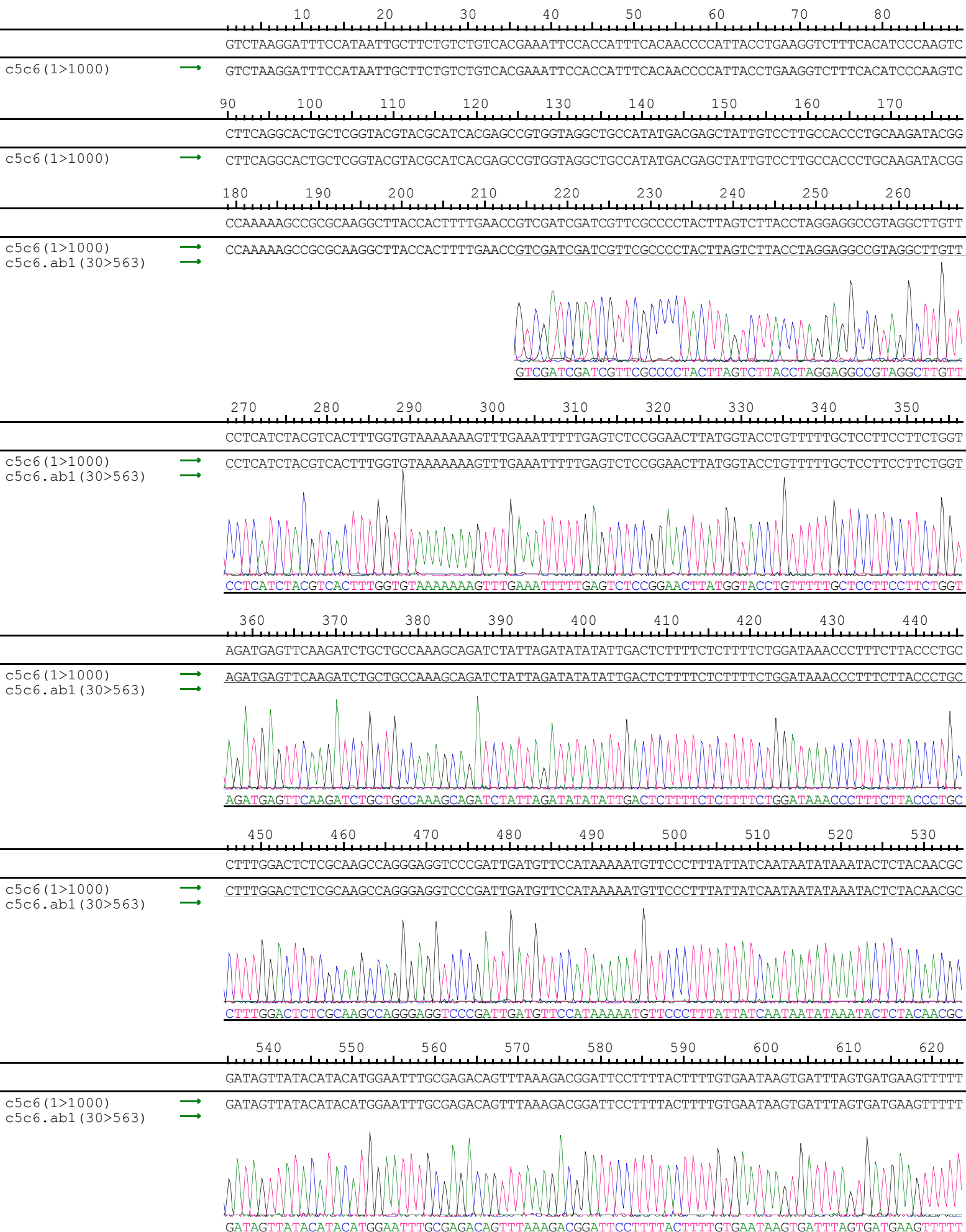

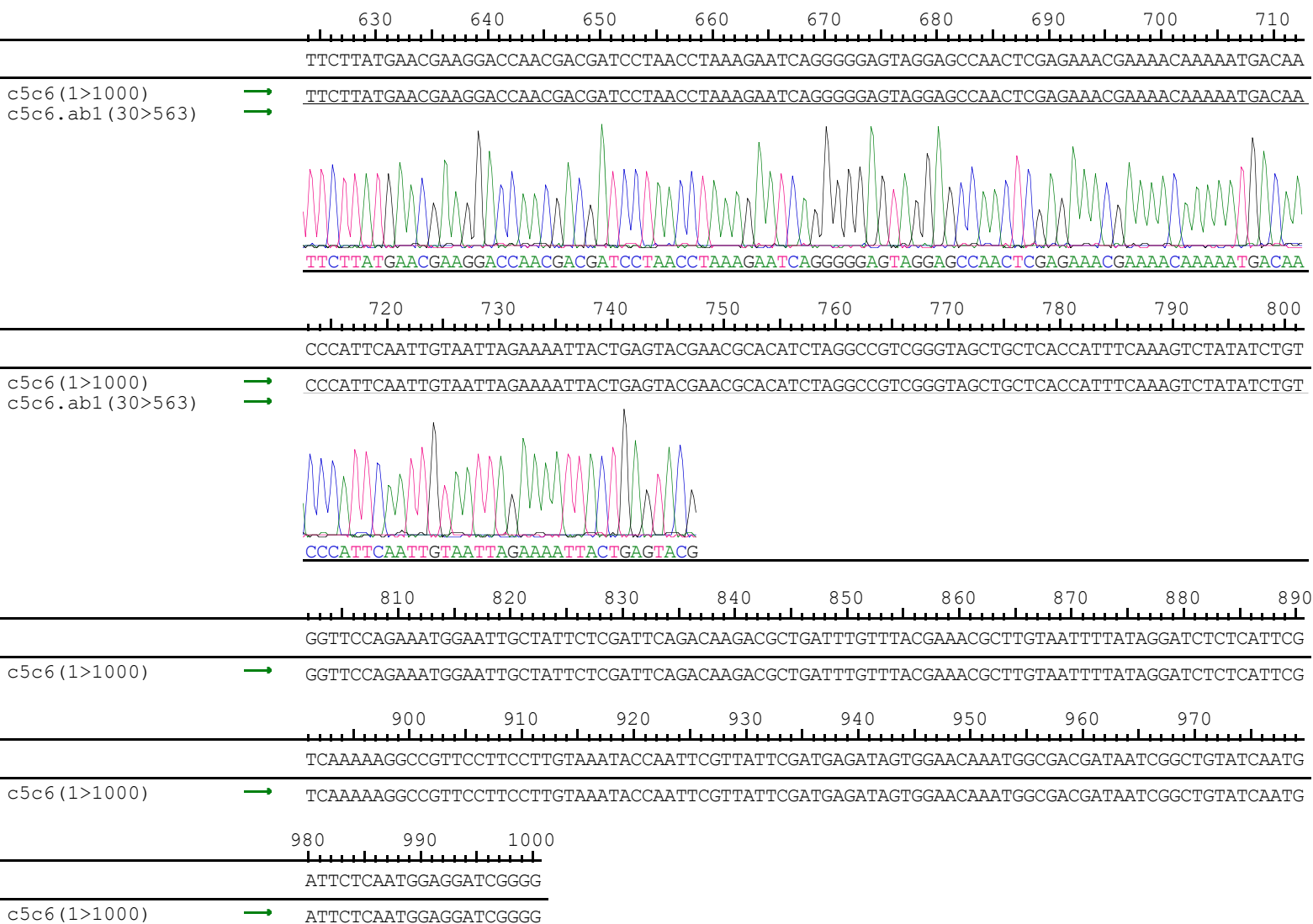

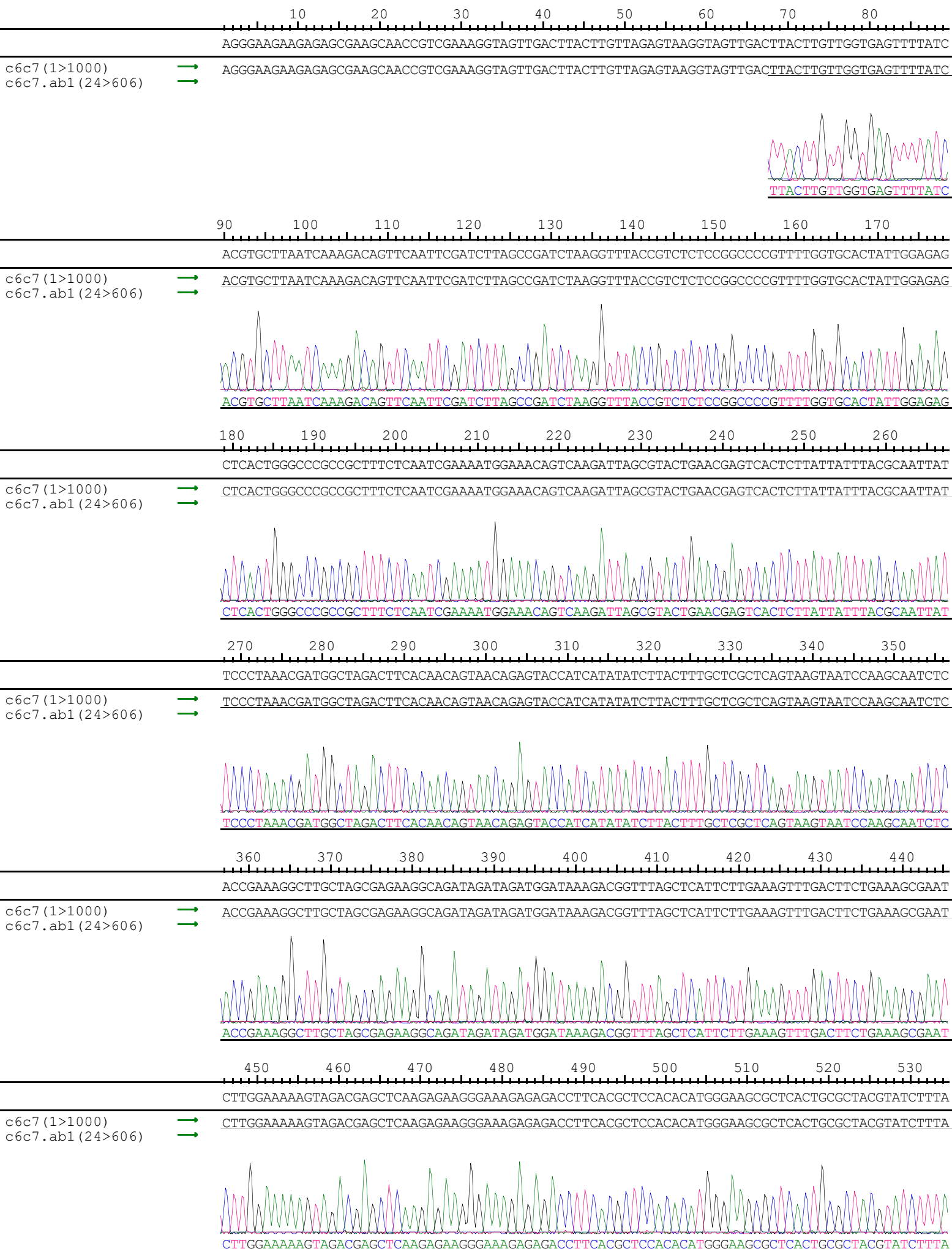

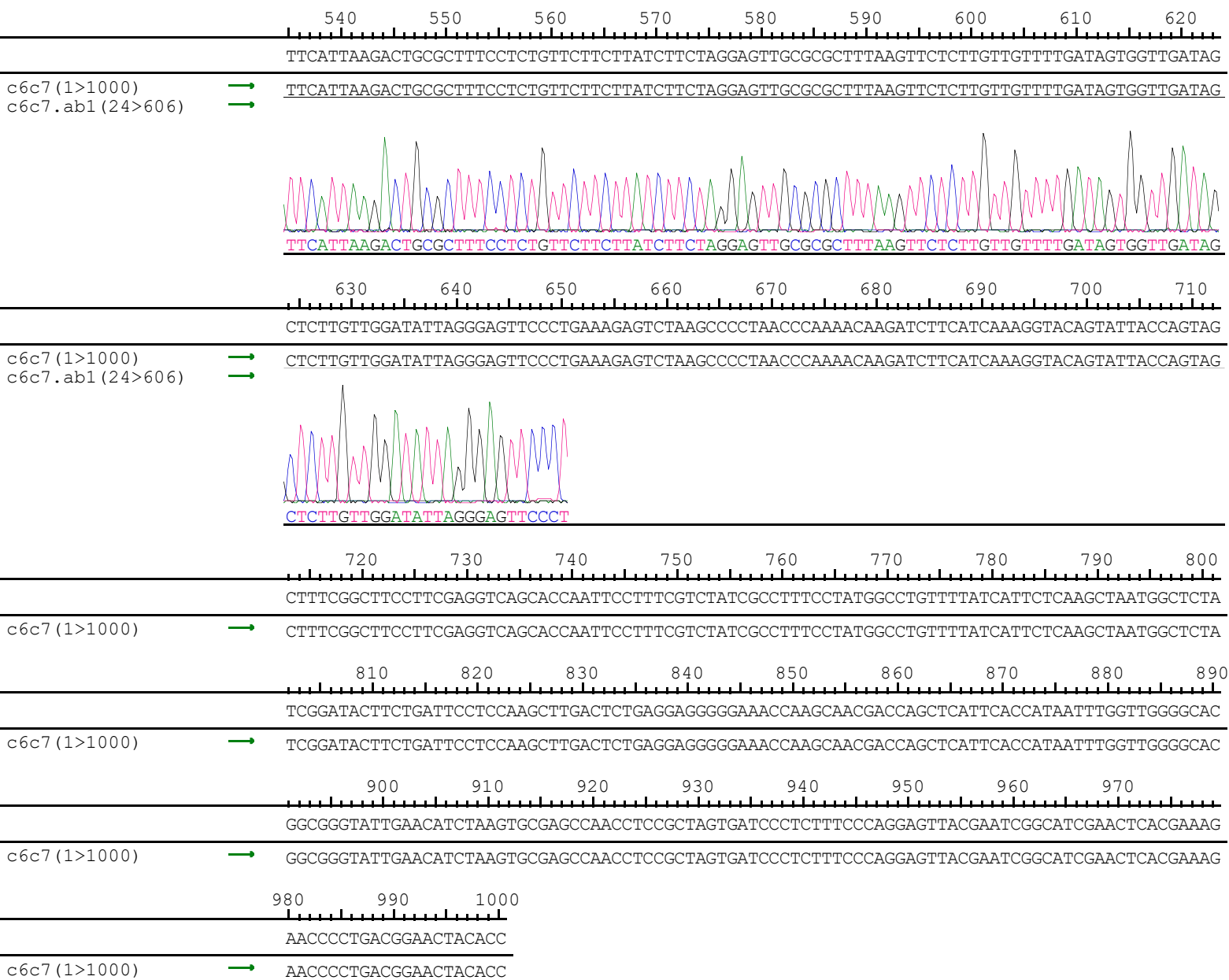

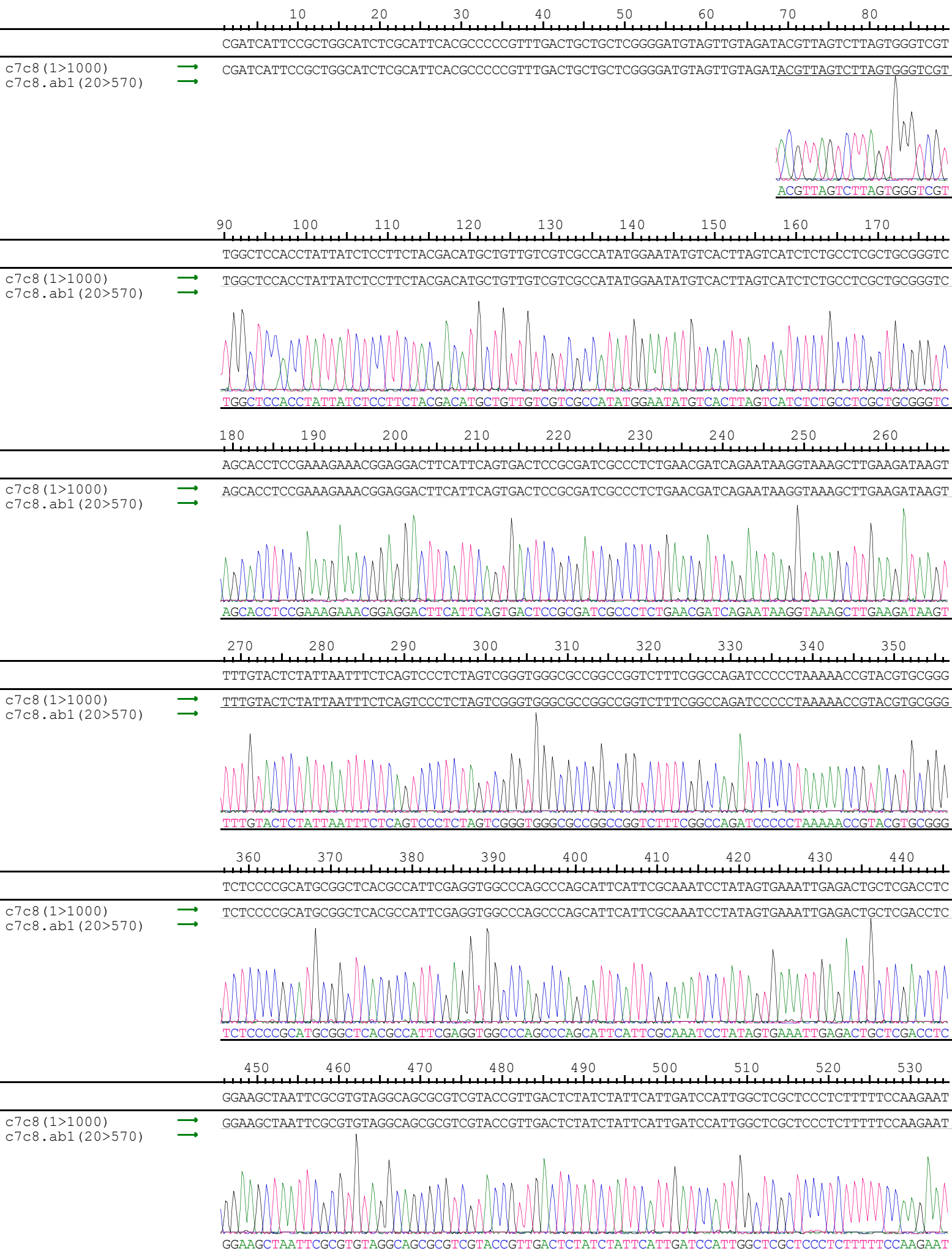

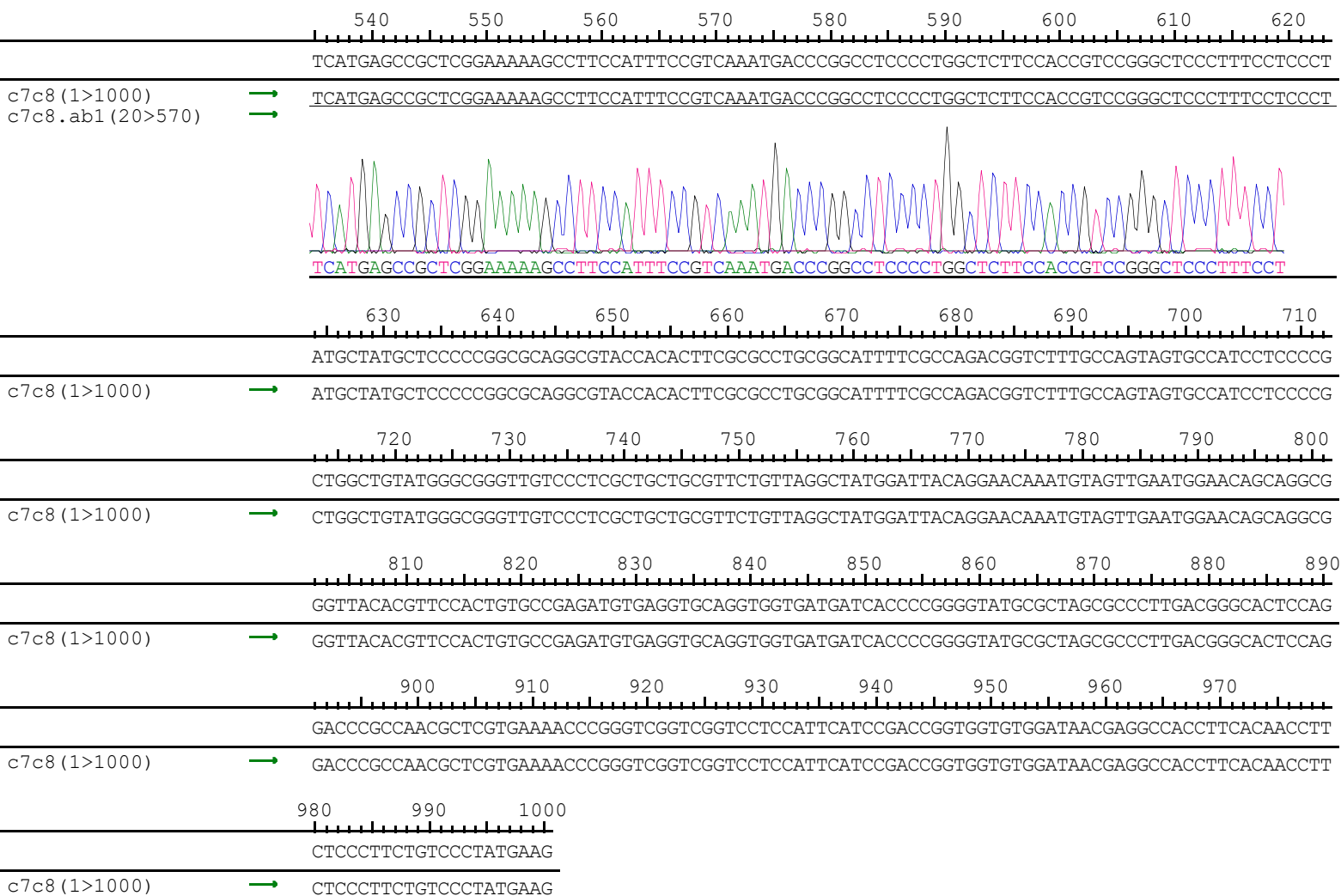

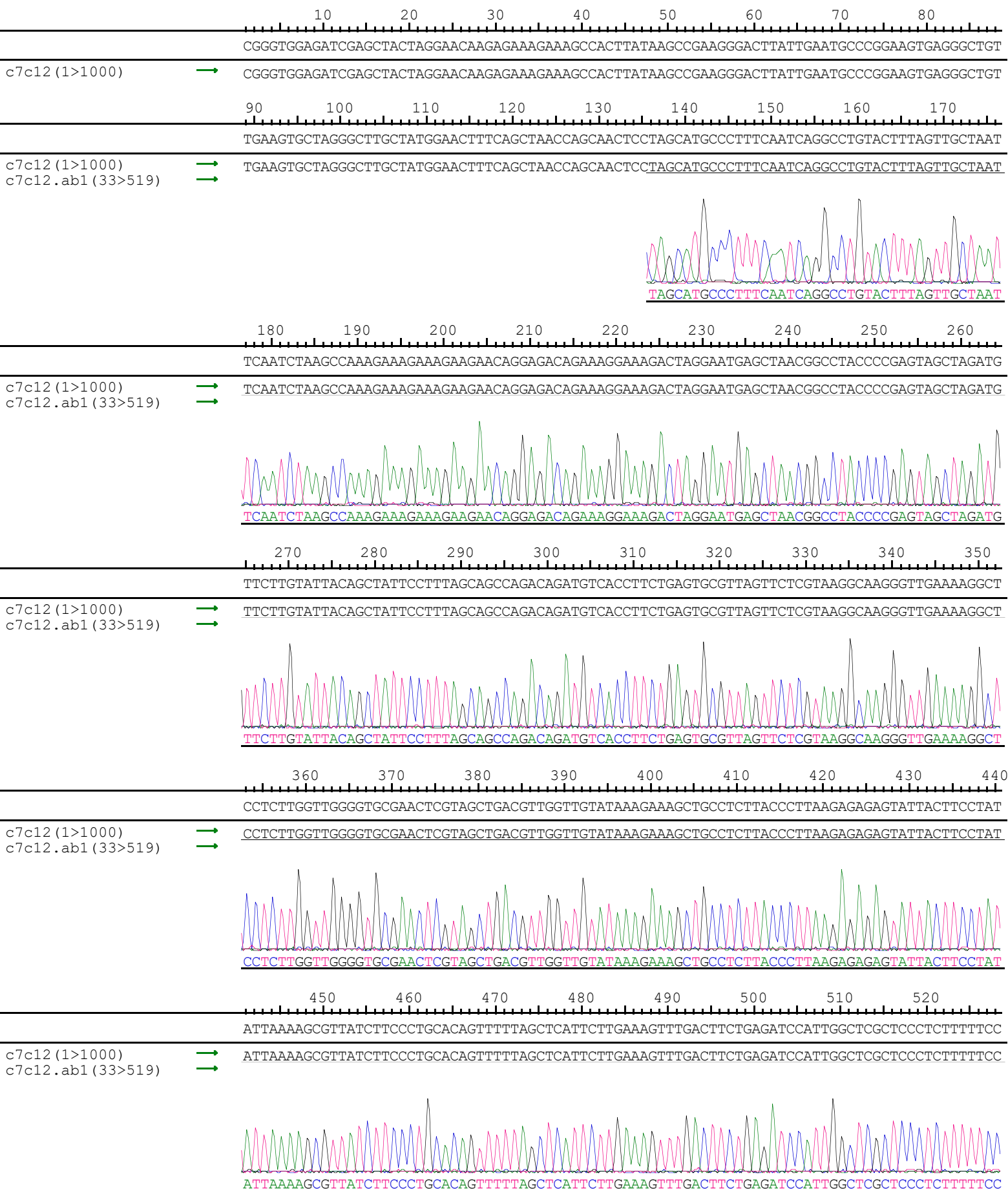

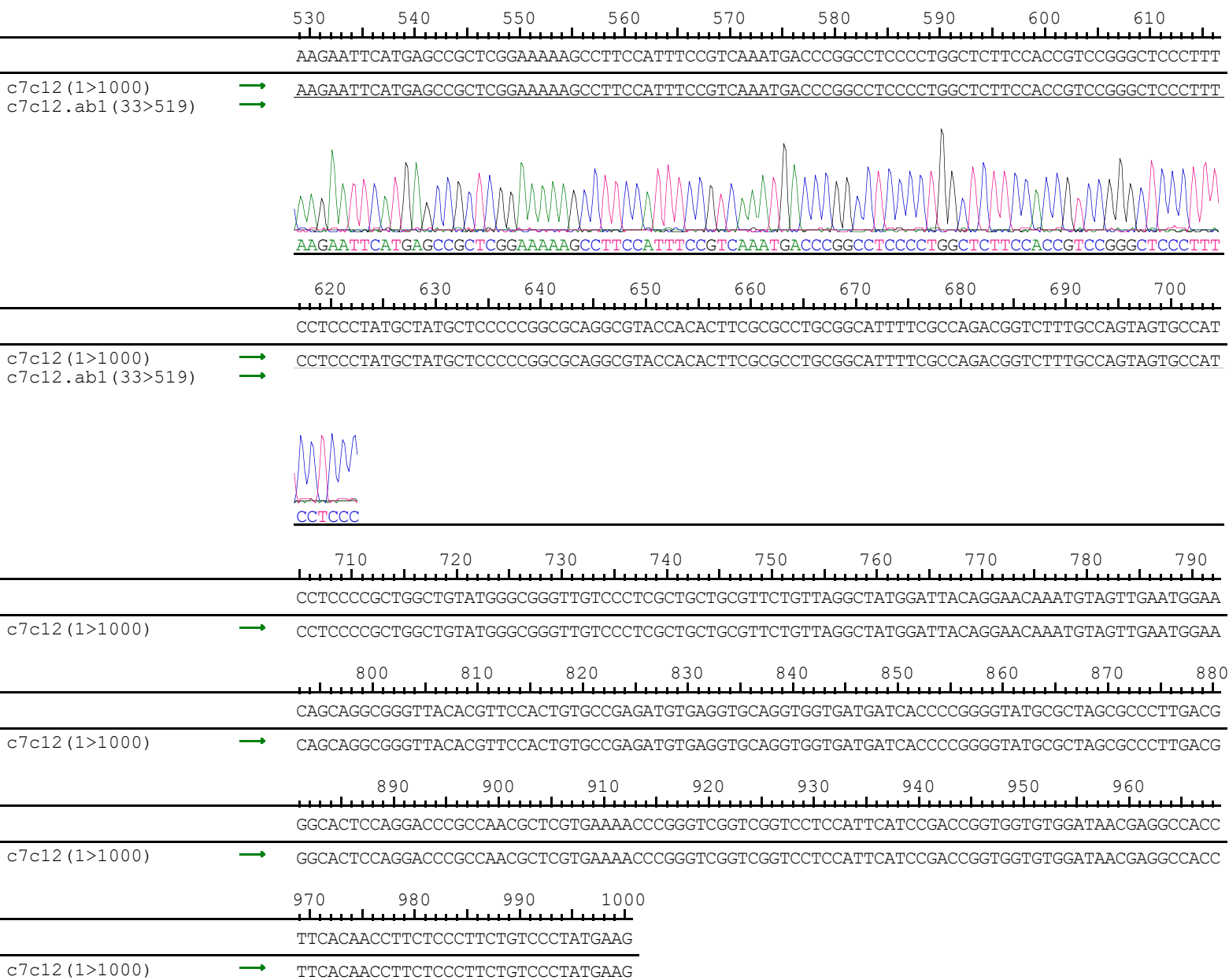

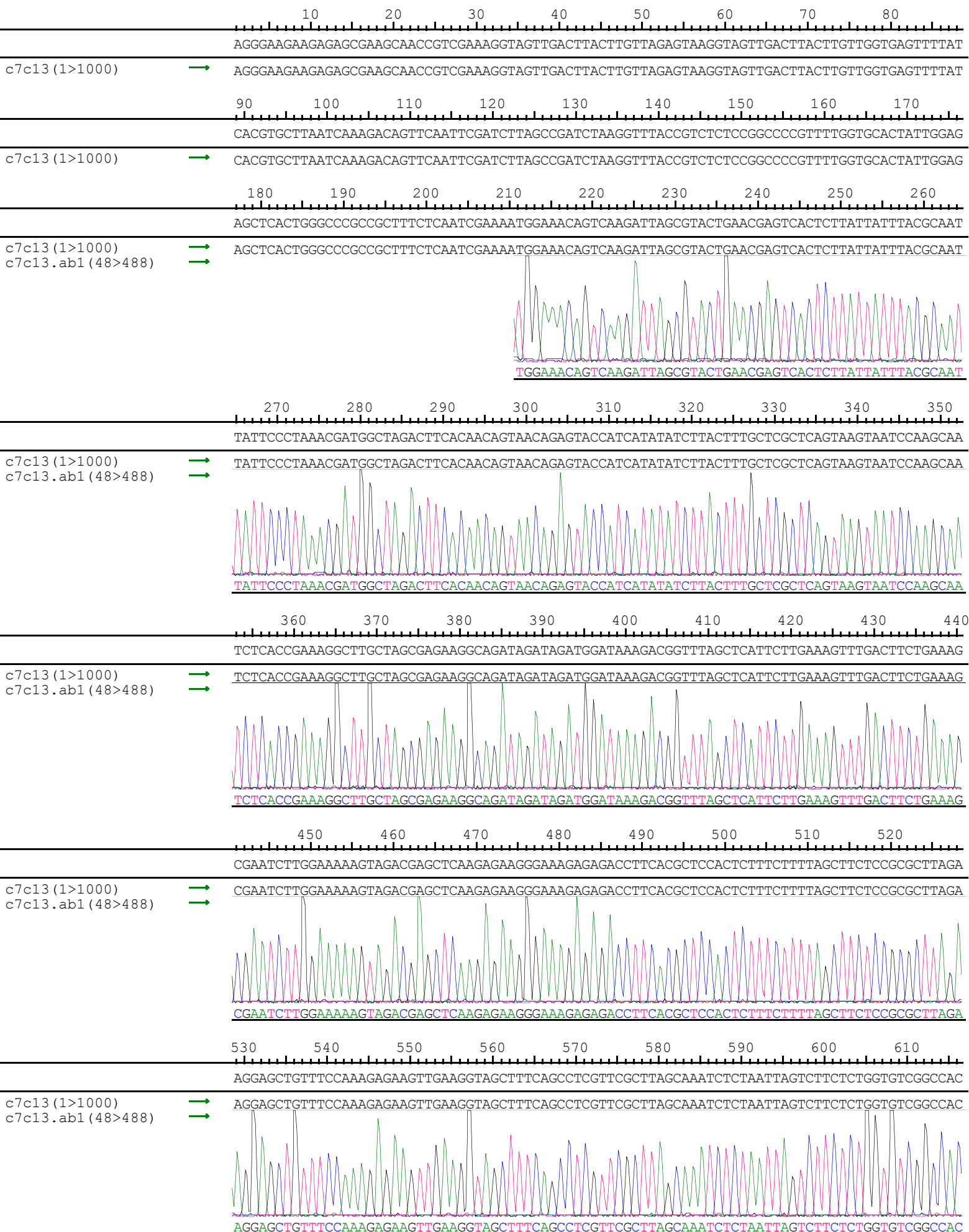

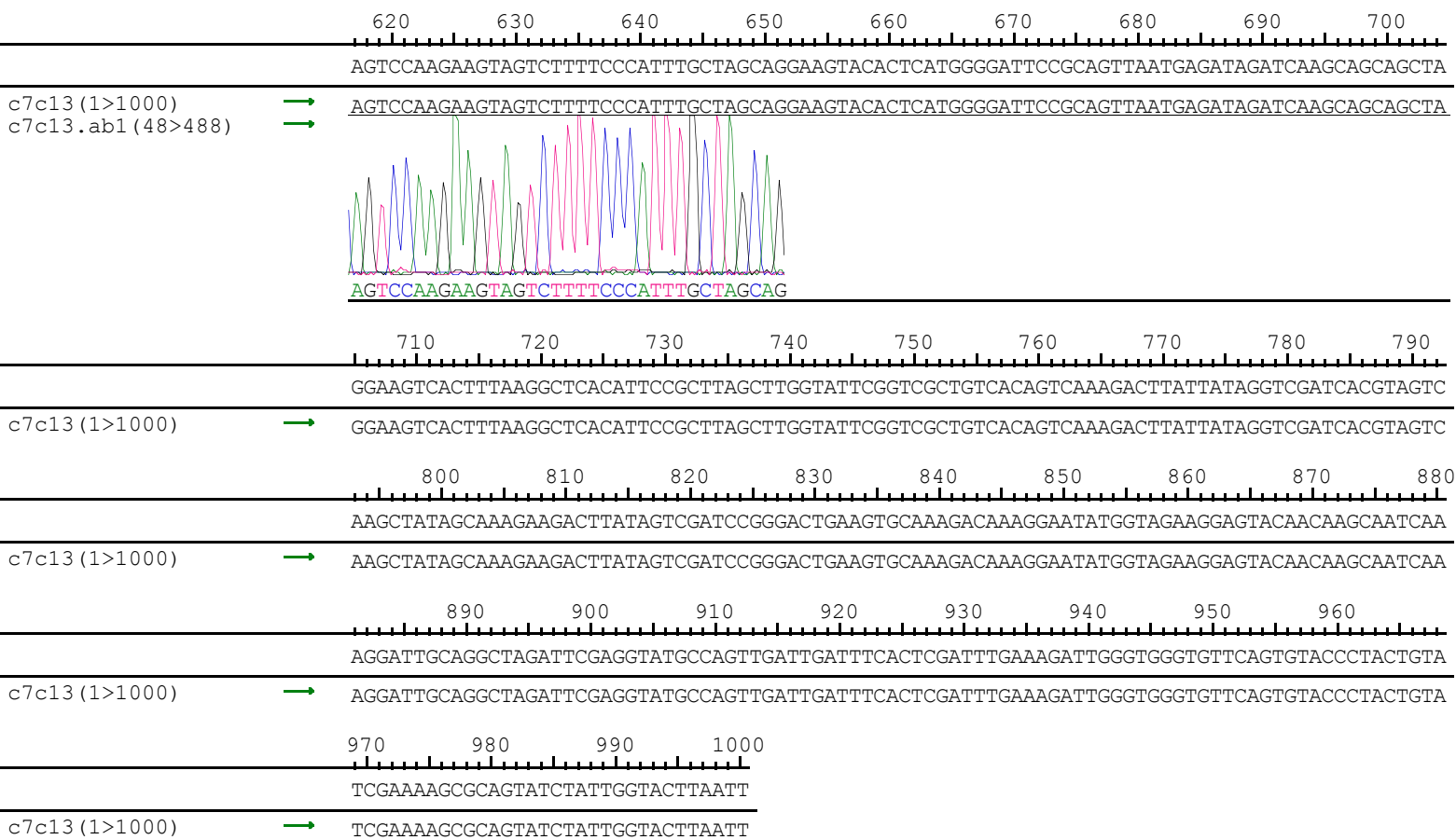

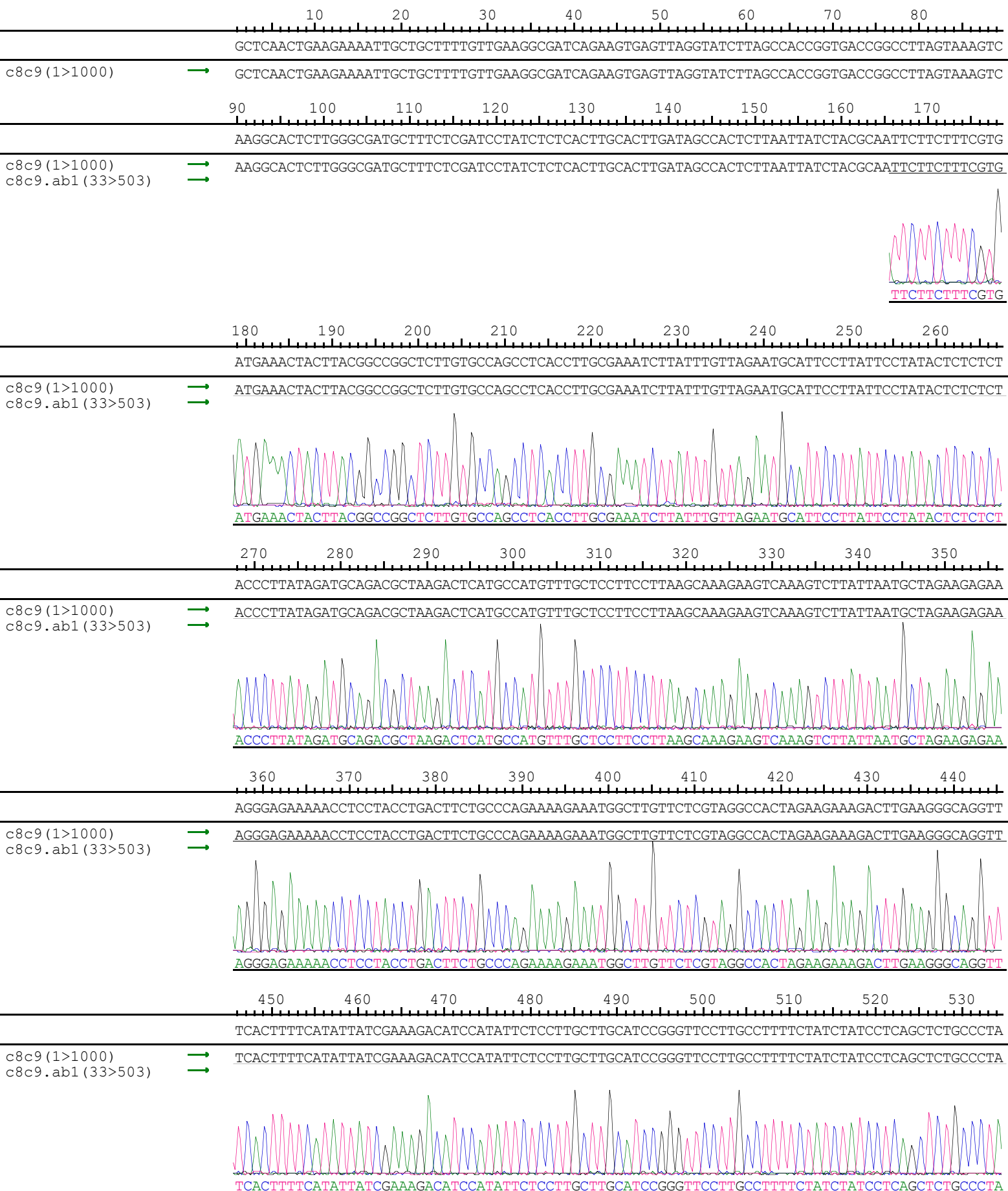

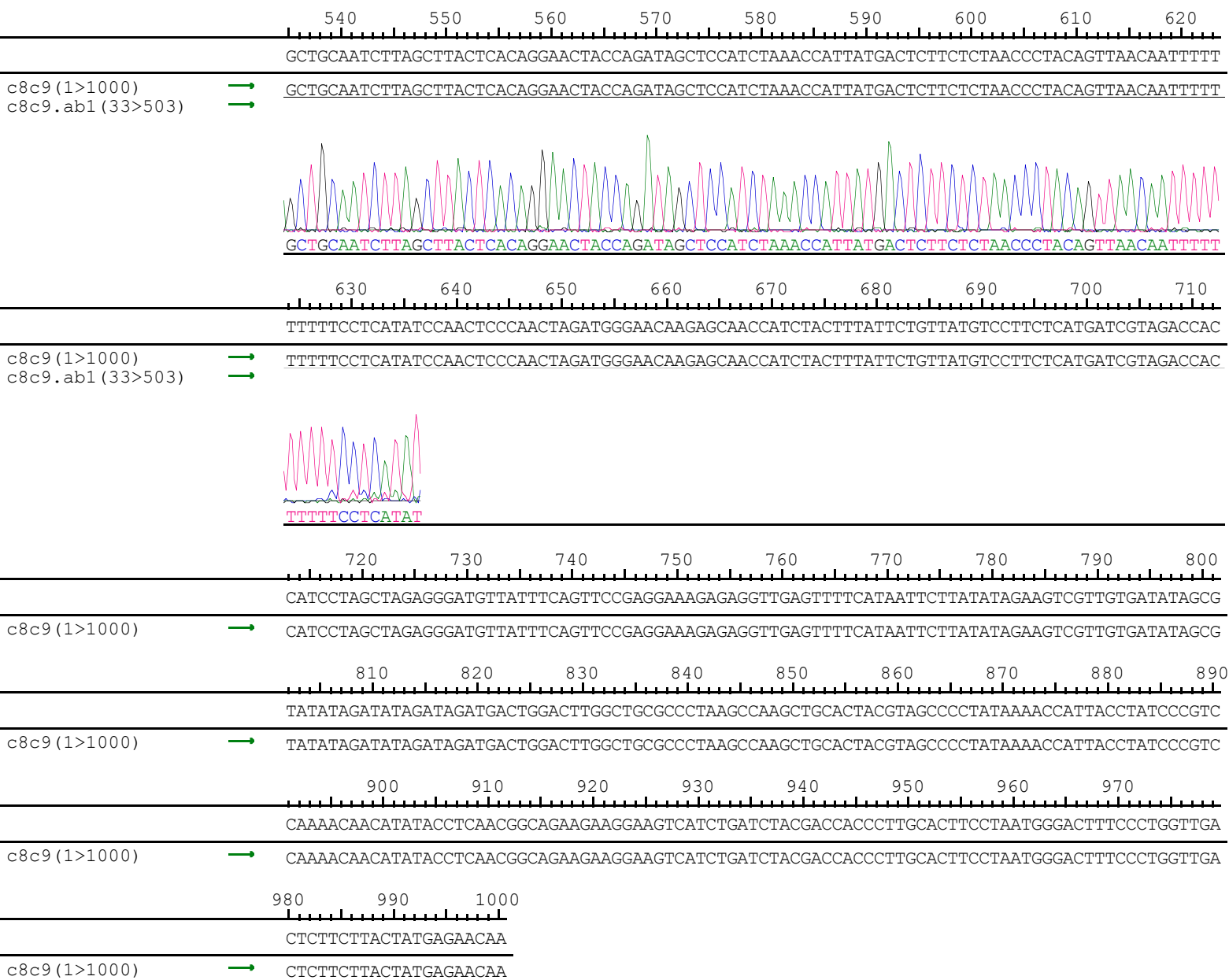

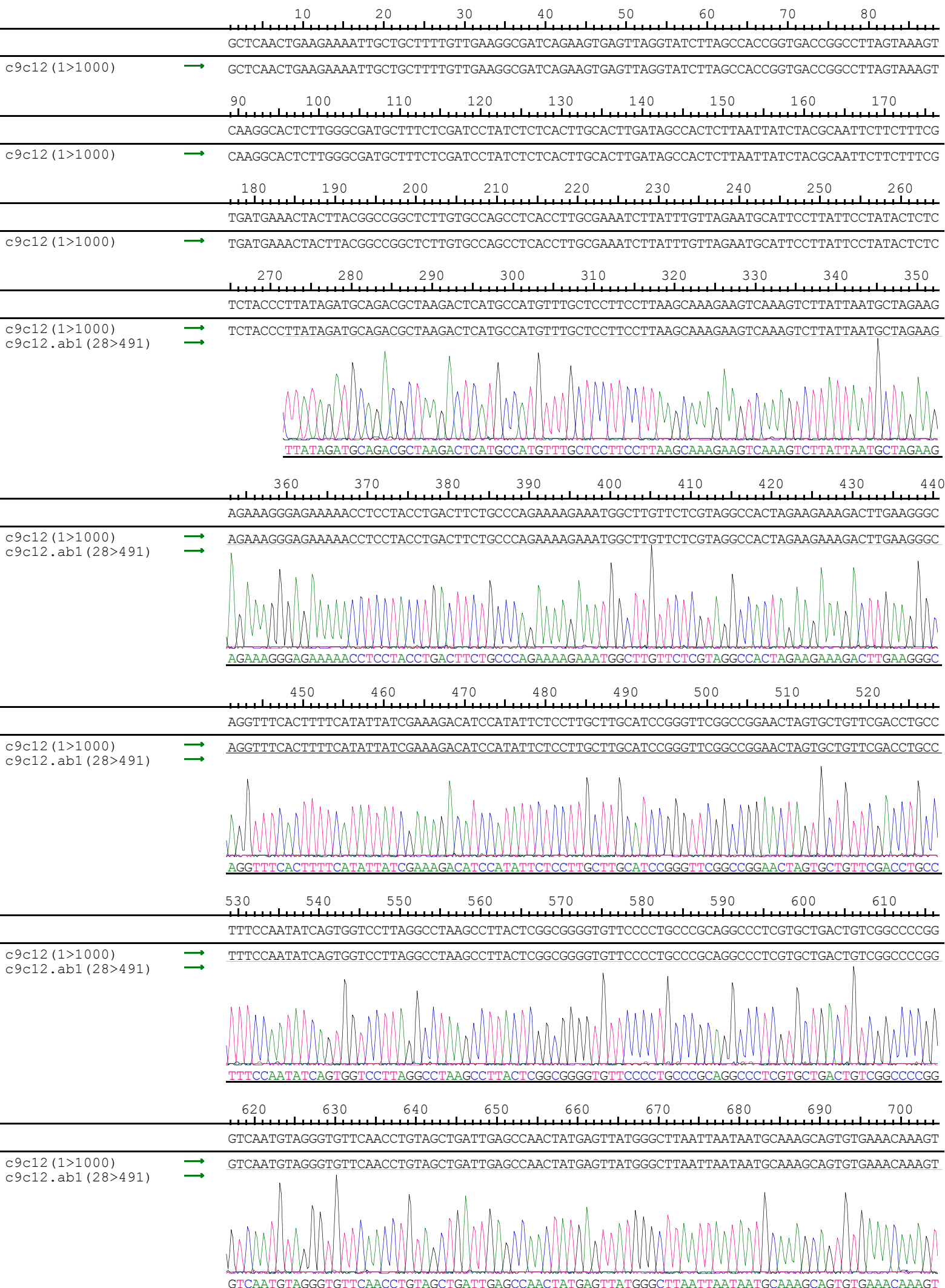

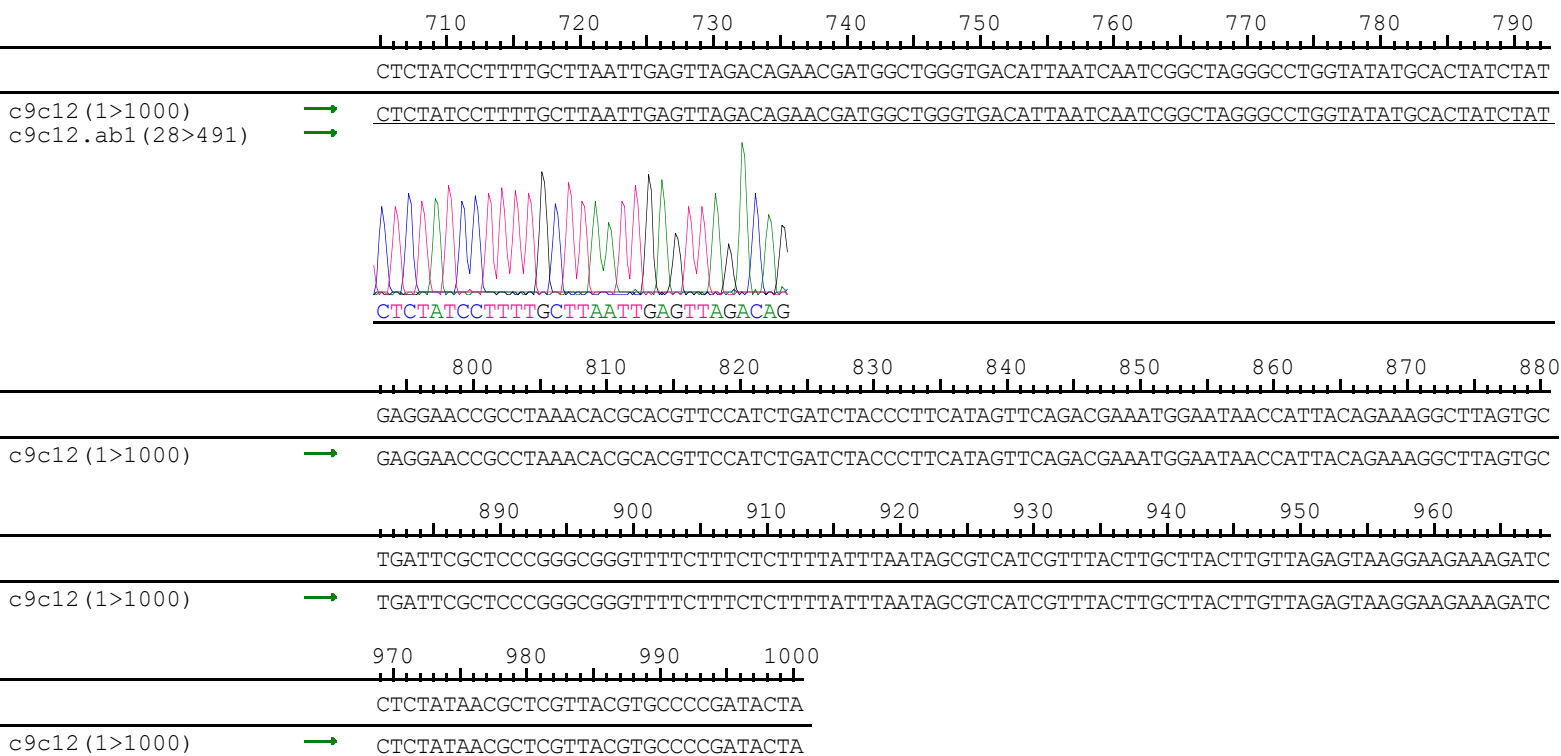

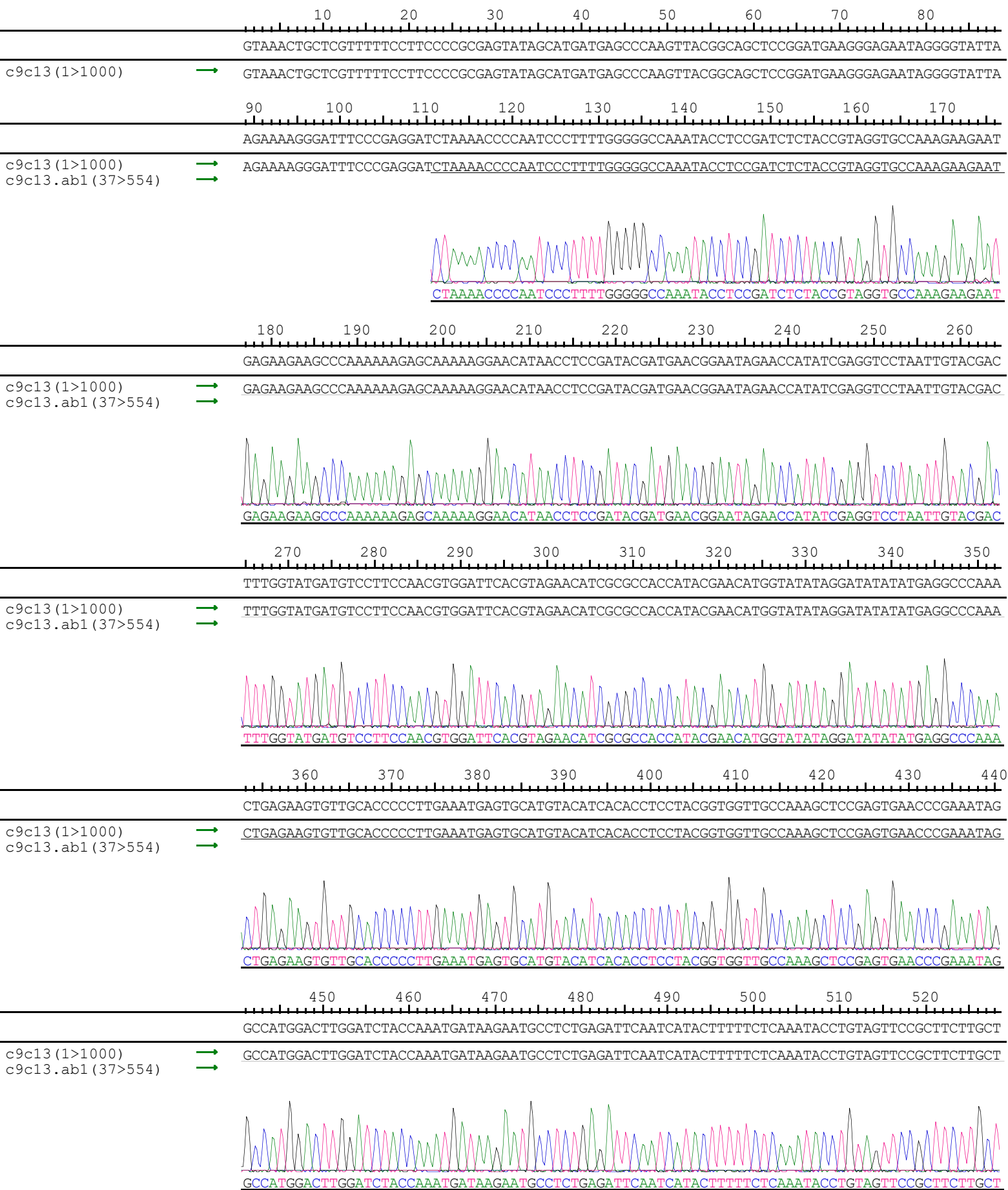

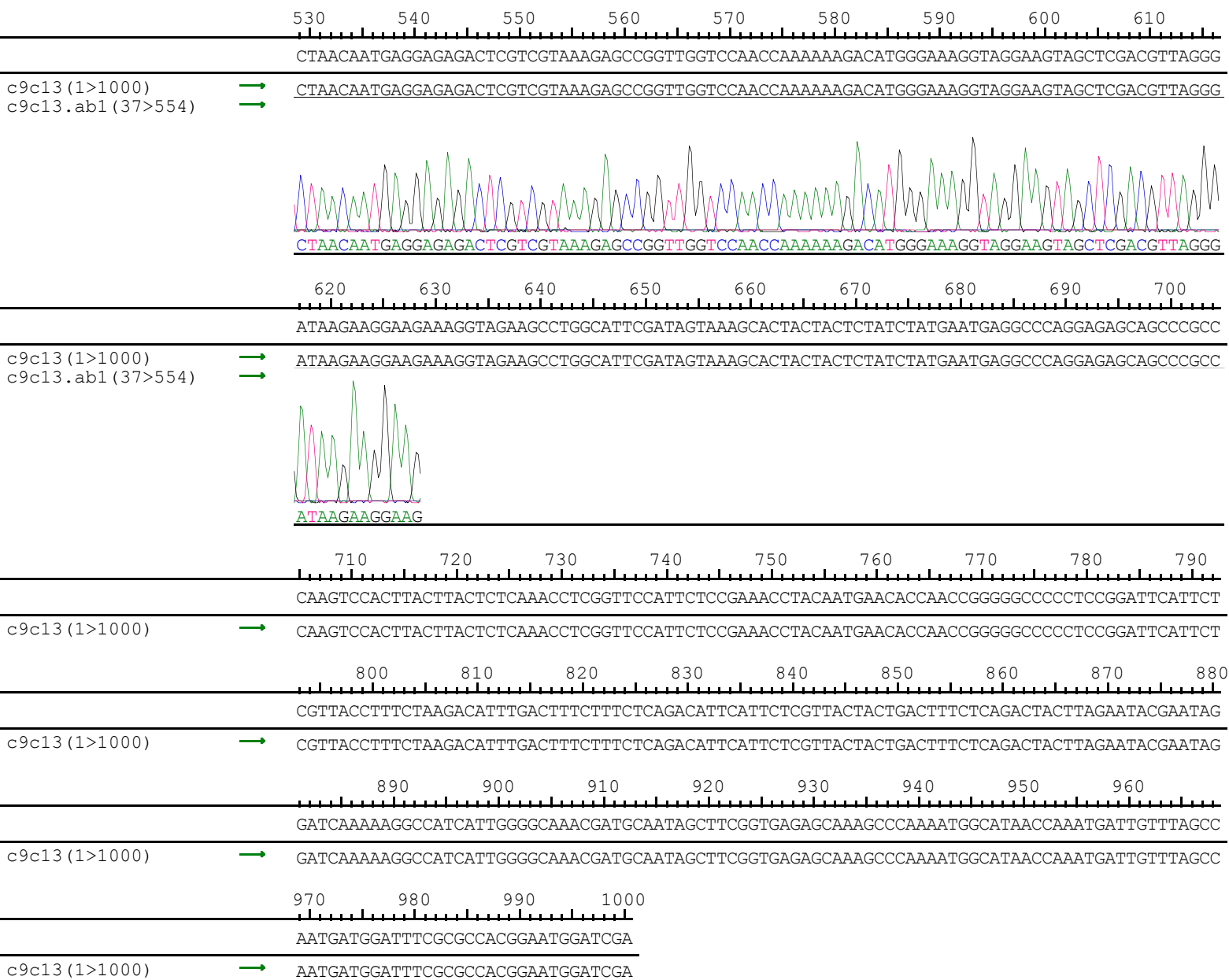

Supplement: Supplementary file 1 [file DataSheet1.pdf]
